# The Skilled, the Knowledgeable, and the Motivated: Investigating the Strategic Allocation of Time on Task in a Computer-Based Assessment

**DOI:** 10.3389/fpsyg.2019.01429

**Published:** 2019-06-27

**Authors:** Johannes Naumann

**Affiliations:** Institute of Educational Research, University of Wuppertal, Wuppertal, Germany

**Keywords:** time on task, PISA, educational assessment, test taking motivation, reading skill, reading strategies, validity

## Abstract

In large scale low stakes assessments, students usually choose their own speed at which to work on tasks. At the same time, previous research has shown that in hard tasks, the time students invest is a positive predictor of task performance. From this perspective, a relevant question is whether student dispositions other than the targeted skill might affect students’ time on task behavior, thus potentially affecting their task performance and in turn their estimated skill in the target domain. Using PISA 2009 computer based assessment data, the present research investigated for the domain of reading digital text whether three variables that can be assumed to predict performance in digital reading tasks, comprehension skill, enjoyment of reading, and knowledge of reading strategies would also predict how much time students would devote to digital reading tasks, and in particular, whether they would adapt time on task to task difficulty. To address this question, two linear mixed models were estimated that predicted the time students spent on a task, and the average time students spent on relevant pages within each task, by the interaction of task difficulty with comprehension skill, enjoyment of reading, and knowledge of reading strategies. To account for time on task being nested in students and tasks, random effects for persons and tasks were included. The interaction of task difficulty with gender and Socio-Economic Status (SES) was included for control purposes. Models were estimated individually for 19 countries, and results integrated meta-analytically. In line with predictions, for both time on task indicators, significant positive interactions were found with comprehension skill, enjoyment of reading, and knowledge of reading strategies. These interactions indicated that in students with high comprehension skill, enjoyment of reading, and knowledge of reading strategies there was a stronger association of task difficulty with time on task than in students low in either of these variables. Thus, skilled comprehenders, students enjoying reading, and students in command of reading strategies behaved more adaptively than lower skilled, motivated, or knowledgeable students. Implications of these findings for the validity of self-paced computer-based assessments are discussed.

## Introduction

In educational assessments, the goal is to infer a test-taker’s latent ability from their performance on a number of tasks. From a psychological perspective however, it is never the latent ability *per se* that determines a test-taker’s performance. For the notion of a latent variable to be meaningful, and for the latent variable to be of explanatory value, there has to be some notion of which psychological (and/or neural) mechanisms account for the latent variable taking on a specific value within a specific individual (e.g., Sternberg, 1986; Borsboom et al., 2003). This means that it is always specific cognitive and metacognitive, as well as motivational, processes that are executed during the test takers’ engagement with the assessment tasks, which determine the test takers’ responses, and thus their estimated abilities. One fundamental process test-takers need to engage in is the allocation of time to individual tasks. This is for two reasons: Firstly, even assessments that are not supposed to be “speeded”, i.e., where test-takers are assumed to have ample time to complete all tasks, in fact do have a time limit. Thus, even in these assessments test-takers need to employ some sort of metacognitive strategy to allocate time to individual tasks. Secondly, the time test-takers spend on assessment tasks is a fairly strong predictor of their task performance, where the strength and direction of the association is dependent on characteristics of both the test-taker and the tasks. Apparently, it is especially hard tasks, that cannot be solved by routine cognitive processing, but instead require deliberate, controlled cognitive processing (see Schneider and Shiffrin, 1977; Shiffrin and Schneider, 1977), or metacognitive processing (see Pressley et al., 1989; Winne and Hadwin, 1998) where positive associations between time on task and task performance (“time on task effects”) arise. This is e.g., true for tasks from domains such as problem solving in technology-based environments (Goldhammer et al., 2014) or reading digital text (Naumann and Goldhammer, 2017). Against this background, it appears beneficial for a test-taker to invest their time especially in hard tasks. Thus, a natural question seems to be which characteristics of a test taker, either cognitive or motivational, will put them in a position where they adequately allocate their cognitive resources, and thus their time on task, to a task’s difficulty. The present research addresses this question for the domain of reading digital text (see e.g., OECD, 2011; Naumann, 2015; Cho et al., 2018). In the following, I will address the ideas that especially students skilled in comprehension (“The skilled”), students knowledgeable of reading strategies (“The knowledgeable”), and students who enjoy reading as such (“The motivated”) are successful in adapting the time they invest in a digital reading task to the tasks’ difficulty, both overall and regarding the processing of relevant parts of the text materials. These ideas will be derived from describing digital reading as task-oriented reading from the perspective of Rouet et al.’s (2017; see also Britt et al., 2018) RESOLV (REading as problem SOLVing)-model, from Pressley et al.’s (1989) model of the Good Information Processor, as well as the literature on item position effects in assessments (e.g., Debeer et al., 2014), and their moderation through motivation (e.g., Nagy et al., 2018a) and self-control (Lindner et al., 2017).

### Comprehension Skill and Task Representation

**FIGURE 1:**
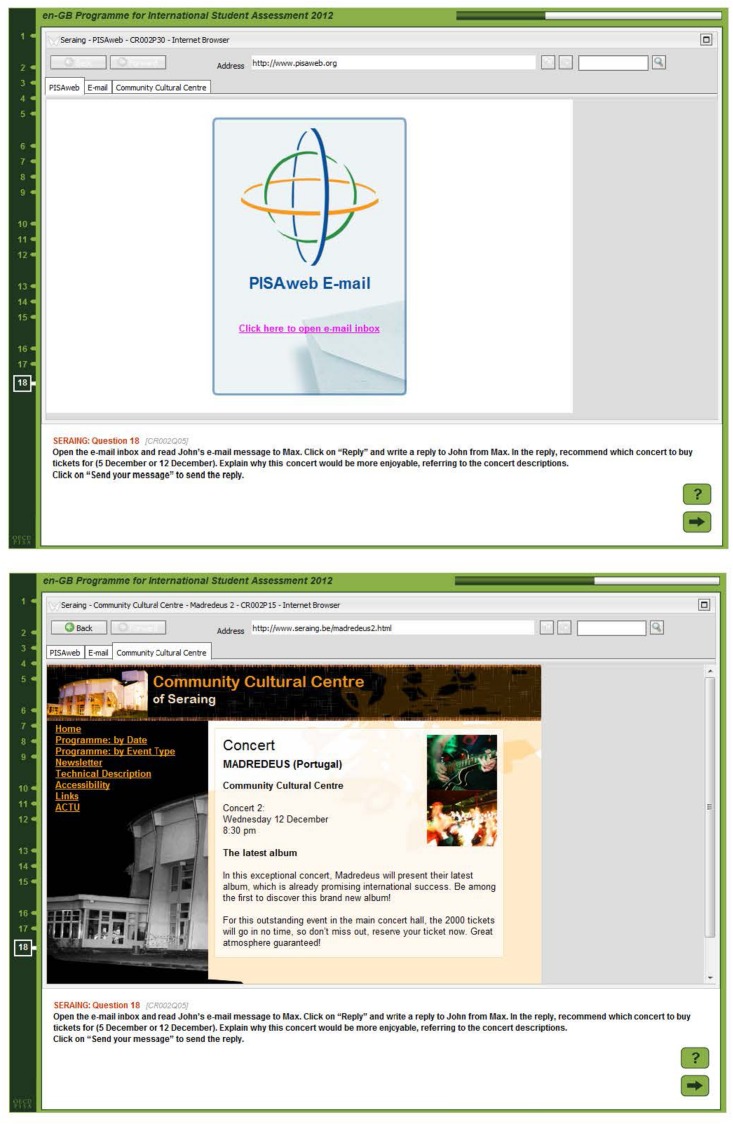
Two screenshots from a digital reading task requiring complex navigation (see OECD, 2011, 2015).

Reading in an assessment situation is an instance of task-oriented reading (e.g., Vidal-Abarca et al., 2010; Salmerón et al., 2015b; Serrano et al., 2018). In many situations, reading as an activity also is not only the processing of textual information to the end that an adequate situation model of the text contents is being built, as described by cognitive models of text comprehension such as Kintsch’s (1998) theory. Rather, especially in opaque information environments such as on line, or when faced with multiple texts that might propose conflicting stances, accomplishing a reading task will entail elements of problem solving (Rouet et al., 2017). When a person reads to solve a task in a reading assessment, they first need to build a representation of the task’s requirements. This includes a judgement of whether the question might be answered by a mere memory search (which will not be the case in most reading assessments, which are designed to not rely on prior knowledge). Then, the person will have to judge which parts of the text, or in a multiple text or hypertext reading scenario, which texts are likely to provide the information needed to answer the question. In addition, the task model might include a judgement of the task’s difficulty, and thus the required degree of scrutiny in processing the textual information. Consider e.g., the task in Figure 1. In this task, students need to compose an e-mail, containing a recommendation to a friend concerning visiting a concert. To accomplish this, students have first to realize that they will need to consult the text. Then, they need to figure out where to find information on the two concerts mentioned in the task instructions, and to match these with the information in the e-mail. As there is no obvious (literal) match between the e-mail and the text on the menu labels in the Seraing Cultural Center’s website, they need to figure out a navigation route, finding the Center’s program, either by “Date” or by “Event type” to get by the required information. To adequately process this information, they need to figure out they have to evaluate it on a semantic level to judge the concert descriptions against the preferences mentioned in the e-mail. In short, students will have to develop a notion that the task displayed in Figure 1 is a fairly complex one which requires a good deal of cognitive effort to be solved.

**FIGURE 2:**
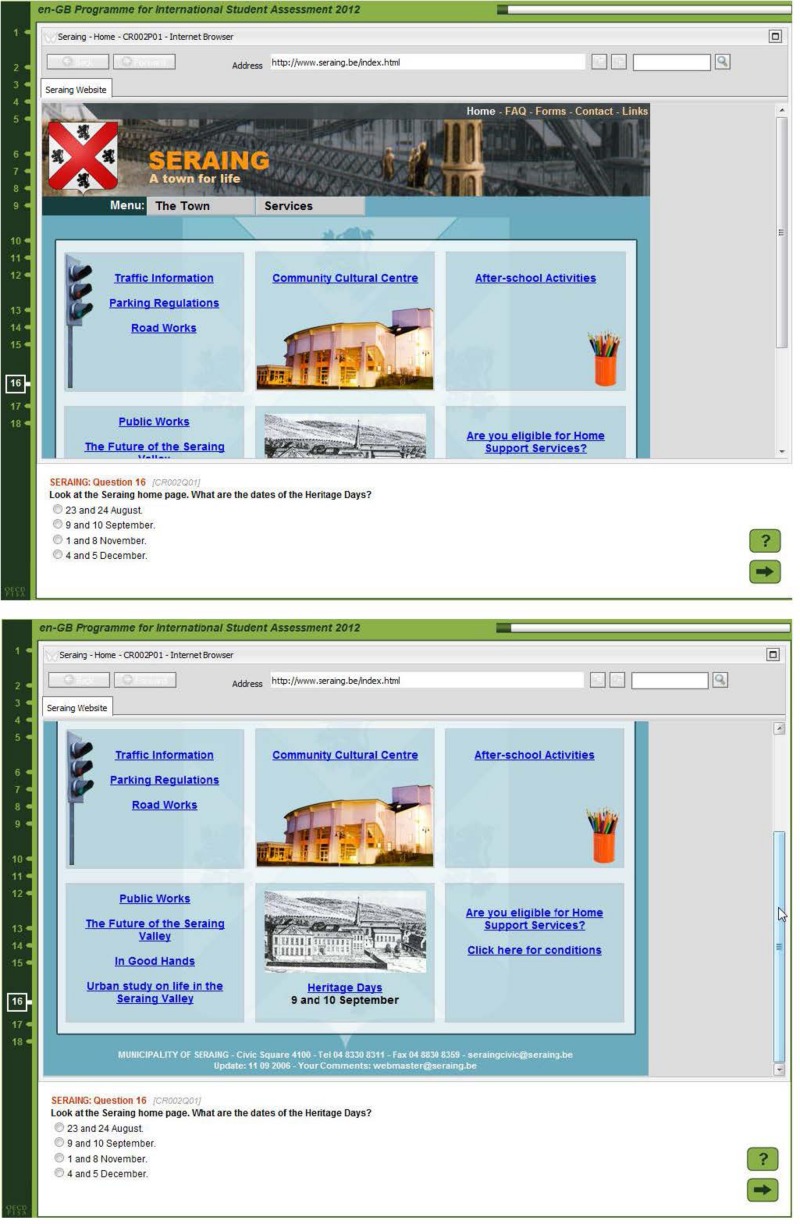
Two screenshots from a simple digital reading task requiring minimal navigation (scrolling) only (see OECD, 2011, 2015).

Consider, in contrast, the task displayed in Figure 2. Solving this task is possible on the basis of comparatively shallow processing that on a mere lexical level matches the name “Heritage Days” appearing in the question to the same name appearing on the page. The only inferencing needed was due to restrictions on screen resolutions in the assessment, students needed to scroll down to find the relevant information. An appropriate task model in this instance will include the fact that only limited cognitive resources, and time, will be needed to solve it (see also OECD, 2015; Naumann and Goldhammer, 2017).

It is likely that skilled comprehenders will be in a better position to arrive at the judgement that the task displayed in Figure 1 needs ample time to be invested in it, while the task displayed in Figure 2 might be solved relatively quickly. Similar to the earlier MD-Trace-Model (“Multiple-Document Task-based Relevance Assessment and Content Extraction”, see Rouet and Britt, 2011), the RESOLV model postulates a process whereby initially only very coarse reading goals are being set. These reading goals are then constantly updated, and the information acquired is judged against some standard specifying whether enough, and correct, information was acquired to meet the reading goal. According to the standards involved in this process, readers may e.g., judge that they need to re-read a passage, that a passage might be skipped, that it might be sufficient to just skim the passage (e.g., the website in Figure 2 for the phrase “heritage days”), or that it might be necessary to carefully read a passage, such as the concert descriptions in the task displayed in Figure 1.

Previous research has indeed found that skilled comprehenders are better in making decision such as these, compared to lesser skilled comprehenders. One central ingredient of building an adequate task model is to note when, and what, information to search for. In line with the notion that an adequate task model is built more easily by better comprehenders, Mañá et al. (2017) found that decisions to search a text for information was predicted by comprehension skills. Moreover, these authors found that only students with average to good comprehension skills had their search decision, and subsequently task performance, boosted in a condition with a delay between reading the text and reading the questions. In line with these results, Hahnel et al. (2018) found that skilled comprehenders were more likely to seek out additional information when necessary in a task that required the evaluation of on line information provided in Search Engine Results Pages (SERPs).

Again in line with the idea that comprehension skills are a condition for building adequate task models, both Cerdán et al. (2011) and Salmerón et al. (2015a) found that students with higher comprehension skills when studying a text comprising multiple documents were much better in selecting relevant materials, and discarding irrelevant materials. This difference was especially pronounced when there were surface cues present, such as a literal match between a phrase in a passage and in the question, but (other than in the task in Figure 2) the passage was in fact irrelevant. Thus, in this scenario, it apparently was good comprehenders who built a task model that (correctly) contained the notion that the surface cue was misleading, and a deeper semantic analysis of the relation between question and text was needed. Similar results were reported by Rouet et al. (2011). These authors found that students in higher grades were less likely to be distracted by semantically irrelevant cues, such as capitalizing, when they had to select hyperlinks from a SERP, than were students in lower grades. A second study showed that indeed parts of this effect could be attributed to students in higher grades having better comprehension skills.

Thus, all in all, if the construction of an adequate task model, that correctly specifies the amount of cognitive effort that has to be invested into a task, is driven by good comprehension skills, we might expect good comprehenders to be better at adapting their time on task to task difficulty in a digital reading situation.

### Reading Strategies and Monitoring

As already mentioned in the introductory section of this article, readers in an assessment need to regulate their allocation of time to tasks. Allocating time on task, and monitoring this allocation through the course of completing a reading task can be seen as an instance of the application of cognitive (e.g., planning) and metacognitive (e.g., monitoring) strategies (see Weinstein and Mayer, 1986). As Pressley et al. (1989, p. 858) put it: “Good strategy users employ efficient procedures to accomplish complex, novel tasks… They possess essential metacognitive knowledge for implementing strategies, *including knowing when and where each strategy might be useful, as well as the costs associated with the strategy, such as the amount of cognitive effort it requires*” [emphasis added]. In line with this notion, a number of studies have found that in basic cognitive tasks, subjects tended to align their allocation of time to task difficulty. For instance, Dufresne and Kobasigawa (1989) found that when children in grades 1, 3, 5, and 7 were given a paired association task, where items in one condition were hard (unrelated) and in one condition were easy (related), 5^th^ and 7^th^ graders spent more time on studying the hard, as compared to the easy items, while 1^st^ and 3^rd^ graders showed no such adaptation of study time (see Lockl and Schneider, 2002, for a replication). Consistent with the idea that these differences in study time reflect metacognitive regulation, Lockl and Schneider (2003) demonstrated that indeed judgements of learning ease (estimated effort to learn the items) were higher for hard than for easy items. Consistent with the idea that subjects differ in their ability to effectively regulate their actual study behavior, they also found that 3rd graders showed higher associations between judgements of learning ease, and actual study time than 1st graders.

Such negative associations between judgement of learning ease (the task being perceived as easy) and time on task are however, not uniformly found. For example, Son and Metcalfe (2000, experiment 1), had undergraduate students’ study eight biographies of famous people, and answer questions about them. Using these rather complex materials (compared to those used by Dufresne and Kobasigawa, 1989; Lockl and Schneider, 2002), Son and Metcalfe found that students indeed spent *less* time studying the biographies they then judged to be harder. One caveat in this case is however, that judgements of effort were confounded with judgements of interest: Not only were the biographies studied longer that were judged to be easier, but also those that were perceived as more interesting. Thus, it might have been the case that the judgement of effort at least amongst other reflected a lack of interest: The subjectively less interesting biographies were studied quicker, and at the same time judged harder *just because* they were less interesting and thus more effort would have to be put in, to compensate for the lacking interest.

All in all, there appears to be ample, though not unanimous, evidence that students who are able to metacognitively regulate their learning activities spend more time on harder, and less time on easier tasks. There is however, only little direct evidence how knowledge of reading strategies – apart and on top of comprehension skill – would shape the time on task behavior of adolescent students in task-oriented digital reading scenarios, that is, in tasks that are way more complex than even the biographies studied by Son and Metcalfe (2000). Once again from the perspective of the RESOLV-Model, we might expect students knowledgeable of metacognitive reading strategies to be especially apt to align their time on task with task difficulty. This is because during the reading or (in the case of an assessment) task solution process, the task model, i.e., a representation of the reading goal and the resources required and available to achieve it, needs to be constantly updated, and this updating metacognitively regulated (Rouet et al., 2017, see last section, see also Winne and Hadwin, 1998).

### Reading Enjoyment and Test-Taking Motivation

Even students who are in good command of comprehension skills, and possess the reading strategy knowledge to successfully build, and through the course of task completion maintain, an adequate task model, might not all alike be motivated to put in the cognitive effort that is required to solve especially hard digital reading tasks. Amongst other lines of research, this is evidenced by studies investigating position effects in low stakes assessments such as PISA. Usually, students’ performance declines over the course of an assessment in the sense that the same task will have a lower probability of being answered correctly when it is presented later in the assessment, conditional on a student’s skill (Debeer and Janssen, 2013; Debeer et al., 2014; Borgonovi and Biecek, 2016; Weirich et al., 2017; Nagy et al., 2018a). Not all groups of students however are prone to show position effects to equal degrees. For example, Borgonovi and Biecek (2016) analyzed position effects using data from the PISA major domains in 2006, 2009, and 2012, i.e., mathematics, reading, and science, respectively. They found that performance declines due to item positions in each domain to be strong especially in boys, and in students coming from lower socio economic status (SES) backgrounds. Similarly, Nagy et al. (2018b) found that especially for the domain of reading, position effects were strong in boys, and lower SES students.

What mechanisms might account for item position effects in general, and for inter-individual variance in the strength of these effects? The decline in performance in general has been attributed to students, over the course of the assessment, being less willing and/or able to put effort into solving the assessment tasks. For example, Weirich et al. (2017) measured test taking effort at two points in time during 9410 ninth-graders’ completion of a science assessment in Germany. They found not only position effects, but these effects, on an individual level, were predicted by the change in test-taking effort that occurred between the two points in time. Lindner and colleagues (Lindner et al., 2017, 2018) discuss position effects in the context of exercising self-control. They define self-control in accordance with Inzlicht and Schmeichel’s (2012) process model of self-control. According to this model, exercising self-control at one point in time will decrease especially the motivation to attend to aversive tasks, and increase the likelihood of attendance to pleasing stimuli at a later point in time. Consistent with this idea, Lindner et al. (2018) found that the decline of performance over the course of a 140 min assessment of mathematics and science was predicted by waning state self-control, measured at seven points in time. Also consistent with this idea, Lindner et al. (2017) found that participants who had been forced to exercise self-control in a later mathematics assessment task exhibited a steeper decline in performance (i.e., stronger position effects) than participants who had not had to exercise self-control. However, contrary to their expectations Lindner et al. (2017) did not find any effects of self-control expenditure on time on task as an indicator of task engagement.

According to Inzlicht and Schmeichel (2012), it is especially effort-requiring and for this reason aversive tasks that are affected by previous expenditure of self-control. In the context of cognitive assessments, this assumption implies that waning self-control (and thus a decline in performance) should be strong especially in those students who perceive the assessment tasks as aversive. A reading task, for instance, might be especially aversive for a person who struggles already with basic reading processes, such as decoding, and in general does not enjoy reading. A fluent reader, in contrast, who also enjoys reading as an activity, from this perspective should be much less prone to exhibit position effects. In line with these ideas, Nagy et al. (2018a) indeed found position effects in a reading assessment to decrease with increasing decoding skill and reading enjoyment on the student level.

Taken altogether, we might expect, both from previous research, and from the perspective of theoretical models such as Inzlicht and Schmeichel’s (2012) model of self-control, that the adaptation of time on task to task difficulty is dependent not only on cognitive variables such as comprehension skill and knowledge of reading strategies, but also on motivational variables. In particular, we might expect that especially students who perceive reading as an enjoyable activity might be willing to invest extra time when encountering a hard task. Students for whom reading is aversive, in contrast, might refrain from this investment, so that the adaptation of time on task to task difficulty should be especially pronounced in motivated readers, who report a high level of reading enjoyment.

### The Present Research

To the best of the author’s knowledge, there is yet no study that investigates how in reading digital text, students’ adaptation of total time on task to task difficulty is conjointly predicted by comprehension skill, knowledge of reading strategies and reading enjoyment. In task-oriented reading of multiple texts in general, and in task-oriented reading situations using digital text in particular, readers need to select which texts, or parts of the text available, to access and to use, in which order to accomplish their goals, and which to discard (“navigation”, see Lawless and Schrader, 2008; Naumann, 2015; Salmerón et al., 2018). Then they have to decide for each text or part of a text selected, how much cognitive effort they want to invest into processing. Naturally, especially in hard tasks, it seems beneficial to devote time to processing task-relevant parts of the available materials (see e.g., Rouet and Le Bigot, 2007). Thus, besides investigating the differential adaptation of total time on task to task difficulty, the present research specifically examined how the time students spend on relevant parts of the text stimulus is adapted to task difficulty by students varying in comprehension skill, knowledge of reading strategies, and reading enjoyment. These questions are addressed using data from one of the first computer-based large-scale assessments, the PISA 2009 Digital Reading Assessment.

The Digital Reading Assessment was an International Option in PISA 2009, which was chosen by 19 countries and economies. It was targeted specifically at students’ skill in engaging with, comprehending, and using digital texts that were prevalent at the time the assessment was conceived (early 2007 to early 2008), such as websites (personal, educational or corporate), blogs, e-mails, or forums. It was comprised of a total of 29 tasks, which were distributed across nine units. Each unit consisted of a text stimulus and between one and four tasks. Each text stimulus was made up of several pages, which in most cases belonged to different texts, such as an e-mail and a website (see Figure 1). Tasks differed in how many pages students needed to access to complete the task, with some tasks requiring to read only the task’s prompting page (see Figure 2), and some tasks requiring the student to perform as many as 13 steps of navigation. Besides pages necessary to complete the task, tasks also varied in their number of relevant pages. Relevant pages were defined as those pages that either contained information that needed, or could be used to solve each task, or that needed to be visited in order to arrive at this information. In addition, pages were considered relevant that, from their labels, could be assumed to hold information instrumental either to solve the task, or to complete navigation, such as a “site map”. The mean number of relevant pages was 3.61 (*SD* = 3.42, *Md* = 2, *Min* = 1, *Max* = 14). However, in each task, all pages of the unit’s text stimulus were available to students, making it possible to visit not only pages that were relevant to the task, but also non-relevant pages.

The PISA 2009 Digital Reading data set lends itself to address the issues raised in a couple of ways. First, computer-based assessments allow for the measurement of time on task, and more so, for a detailed investigation of what parts of a task stimulus (in this case: the text[s]) students encountered for how long, and in which sequence. This makes it possible to derive measures of task engagement, such as the average time spent on relevant pages, which are not routinely available from paper and pencil tests (see Greiff et al., 2015). Second, a number of tasks large enough to model a random effect for tasks is available. Thus, other than in fixed effects models such as ANOVA or OLS regression, which allow generalization only to other persons, but not to other situations, conditions, or tasks than those specifically employed in the respective design, here the obtained results can in principle be generalized to other tasks that were constructed according to the same framework through modeling task as a random effect (De Boeck, 2008). Third, since reading was a major domain in PISA 2009, rather detailed student-level measures are available, not only as to their comprehension skill, but also as to their knowledge of reading strategies, and their enjoyment of reading. Finally, large scale databases provide not only good variation in terms of students’ backgrounds, but also good opportunities to control for background variables such as SES and gender. In the present case, this seems especially crucial, as on the grounds of the results reported by Nagy et al. (2018b) and Borgonovi and Biecek (2016, see section “Reading Enjoyment and Test-Taking Motivation” above), it might well be expected that higher SES students and girls are more likely to adapt their time on task behavior to task difficulty than are their lower SES peers or boys: As it seems, higher SES students, as well as girls, are more prepared than their lower SES or male peers to maintain cognitive effort in an assessment. This means that these background variables also are likely to affect students’ preparedness to adapt their time on task behavior to task difficulty. Thus, any analysis targeting time on task behavior conditional on task difficulty should control for the interaction of SES and gender with task difficulty.

## Materials and Methods

### Subjects

Subjects were those students that participated in the PISA 2009 Digital Reading Assessment and for whom time on task for at least two tasks, comprehension skill, knowledge of reading strategies, and enjoyment of reading were available (*N* = 32,669, country-wise 930 ≤ *N* ≤ 2800, see Supplementary Material 1 for country-wise *N*’s). Overall, there were 50% boys. There were between 46 and 53% boys in each sample. Due to PISA’s sampling scheme, which samples students at the end of compulsory education, students were between 15.17 and 16.33 years old (*M* = 15.78, *SD* = 0.29; country-wise *M* between 15.67 and 15.87).

### Measures

#### Total Time on Task

Time on task was read from log files. It was defined as the time that elapsed between the onset of the task, and the time the student gave a response. It thus comprised the time a student spent reading the task instruction, reading potentially both relevant and irrelevant parts of the text, and deciding on a response. To account for the skew of the time on task distribution, the natural logarithm of the total time on task was used.

#### Time on Relevant Pages

To compute time on relevant pages, each navigation sequence was segmented by page transitions. Then the time elapsed between each transition to, and from, a page classified as task-relevant was summed up across each task-completion sequence. Since in each task the prompting page was defined as relevant, time on relevant pages also comprised the time spent reading the task instruction. It did however not comprise time a student might have spent reading task-irrelevant parts of the stimulus. Because tasks varied considerably in the number of relevant pages they comprised, time on relevant pages was standardized at the number of relevant pages available in each task. To account for the skewness of the distribution, the natural logarithm of time on relevant pages was used.

#### Comprehension Skill

Comprehension skill was measured through the PISA 2009 print reading assessment. Being a major domain in 2009, print reading skill was measured with a total of 131 items in 37 units (a unit consists of a text stimulus accompanied by either a single, or multiple items). These 131 items were allocated to 13 clusters worth of approximately 30 min of testing time each. The clusters were assigned to 13 different booklets together with items from the PISA mathematics and science assessments. Each booklet contained four clusters. Of these 13 booklets, one contained four clusters of reading items, three contained three clusters of reading items, seven contained two clusters of reading items, and two contained one cluster of reading items. Thus, each student completed at least 30 min of print reading, with 12 out of 13 students completing at least 60 min (see OECD, 2012, p. 29–30 for details). Items had been constructed according to an assessment framework (OECD, 2009) specifying three different reading aspects, or cognitive operations: (1) Accessing and retrieving, (2) integrating and interpreting, and (3) reflecting and evaluating textual information, as well as two different text formats, continuous and non-continuous texts (see OECD, 2009). It is important to note that in both continuous and non-continuous texts in the print reading assessment students were prompted with the complete text, thus, no navigation in the sense of physical access to text through hyperlinks was required. Comprehension skill was scaled according to the Rasch Model. Weighted Maximum Likelihood Estimates (WLEs) were used in the present analysis. The WLE reliability was 0.84 (see OECD, 2012, p. 194, Table 12.3).

#### Knowledge of Reading Strategies

Knowledge of reading strategies was measured with two reading scenarios. In each scenario students were prompted with a specific reading situation. These reading situations were the following: (a) “You have just read a long and rather difficult two-page text about fluctuations in the water level of a lake in Africa. You have to write a summary”, and (b) “You have to understand and remember the information in a text”. Each of these reading scenarios were accompanied by either 5 (summary scenario) or 6 (understanding and remembering scenario) possible strategies such as “I try to copy out accurately as many sentences as possible” (summary) or “I quickly read through the text twice” (understanding and remembering). In each scenario, each strategy had to be rated by students on a 6-point rating scale from “not useful at all” to “very useful”. It is important to note that the students did not rank-order the strategies themselves, but rated them for their usefulness independently from each other”, and these ratings were then in a second step ranked-ordered within each scenario and student. At the same time, the strategies had been rated, and rank-ordered, by reading experts. The scoring then was accomplished on the basis of the agreement between the rank-order of each student’s ratings with the experts’ ratings’ rank-order. Specifically, 1 point was awarded for each pairwise comparison in students’ ratings that agreed with the respective pairwise comparison in the experts’ rating for those 9 (understanding and remembering) and 8 (summarizing) pairs of strategies where there was consensus amongst the experts which strategy was more useful. A point was only awarded when students, in agreement with experts, ranked a strategy to be *more* useful than another. Thus, when two strategies that entered the score were ranked as *equally* useful by a student, no point was awarded (see OECD, 2012, p. 282). The possible score thus ranged between 0 (no agreement) and 17 (agreement in all 17 pairwise comparisons considered).^1^ The reliability (Cronbach’s α) for the 17 pairwise comparisons entering the score was 0.84 in the present sample, the EAP reliability was 0.86.

#### Reading Enjoyment

Enjoyment of reading was measured through 11 items such as “Reading is one of my favorite hobbies” or “For me, reading is a waste of time”, which were to be answered on a 4-point Likert scale ranging from “Strongly disagree” to “Strongly agree”. Item wordings and item parameters can be found in OECD (2012, p. 290). For the present research, the enjoyment of reading index provided in the OECD PISA 2009 data base was used. Reading enjoyment was scaled according to the partial credit model, providing a Weighted Maximum Likelihood Estimate (WLE) for each student. The reliability (Cronbach’s α) for the present sample was 0.89.

#### Task Difficulty

Task difficulty was defined using the item difficulties of the PISA 2009 digital reading items. In PISA, items are scaled according to the Rasch model. The simple logistic model is applied to dichotomous items, while partial credit items are scaled according to the partial credit model (Masters, 1982). Of the 29 reading tasks in the digital reading assessment, eight had partial credit. Item difficulties (delta) were taken from the international calibration of the PISA 2009 digital reading items, which are provided in OECD (2012, Table A4, p. 343). For partial credit items, this parameter marks the location of the latent ability continuum where the likelihoods of a responses in the highest and the lowest response category are equal (see e.g., Adams et al., 2012).

#### Socio-Economic Status (SES)

To measure students’ SES, the PISA ESCS index was used, which is composed of students’ parents’ occupational status, students’ parents’ education, and wealth, as well as cultural and educational resources in students’ homes (including, but not limited to, the number of books at home). Technically, the ESCS is a factor score from a principal component’s analysis of the HISEI (highest parental occupation amongst a student’s parents), and the PISA home possessions index (HOMEPOS). Details on how the ESCS was computed in PISA 2009 can be found in OECD (2012, p. 312–313).

### Procedure

Students were tested in schools during school hours. First, students completed the paper-based cognitive assessment (reading, mathematics and science), which lasted for two hours. Students could take a break after one hour. Afterwards, the student questionnaire was administered. Last, students completed the computer-based reading assessment. In PISA 2009 digital reading skill was the only domain in the computer-based assessment. Digital reading items were presented in a secure test environment where a browser was simulated that had all typical features of commercial web browsers at the time the assessment was conceived. Items were presented unit by unit, and in each item, the unit’s text(s) were accessible, regardless of whether they were relevant to the item at hand or not. After giving a response, students could not go back to correct their response. Testing time in the Digital Reading Assessment was 40 min. Students knew in advance how much time in total there was to complete the assessment. In addition, students first completed a 10-min tutorial where they could make themselves familiar with the testing environment and simulated web browser. The assessment was not speeded, as indicated by a small number of not-reached items (0.4 on average, see OECD, 2012, chapter 12).

All testing and other data collection instruments and procedures were approved by the PISA governing board, composed of country representatives of all countries that participated in the assessment, as well as by the PISA consortium, led by the Australian Council for Educational Research. Implementation of data collection and management was overseen by national centers, led by national project managers, in each country (see OECD, 2012, p. 24–25 for details). The data that are used for the present research are either in the public domain, and can be found at http://www.oecd.org/pisa/data/ (accessed March 01, 2019), or, where this was not the case, the author had received written consent from OECD to use the Digital Reading Assessment log file data for scientific purposes to be published in scholarly journals. An ethics approval was thus not required for this study as it presents a secondary analysis of OECD data. The author of the present article at no point had access to information identifying individual subjects.

### Statistical Modeling Approach

#### Linear Mixed Model and Estimation

To account for item-specific response times being nested both in items and students, a linear mixed model (LMM) framework was employed that specified crossed random effects for student and item intercepts, and an additional random effect for schools to account for the fact of students being nested in schools due to the PISA sampling procedure. The central research questions were addressed by regressing time on task on the student level variables comprehension skill, knowledge of reading strategies, and reading enjoyment, and the task-level variable task difficulty, as well as, most importantly, the interaction of each student level variable with task difficulty. On top of the main effects and the three two-way interactions of comprehension skill, knowledge of reading strategies, and reading enjoyment with task difficulty, the model contained all other possible two, three and four-way interactions between the four theoretically relevant variables. Gender and SES were entered as control variables. Since the theoretically relevant effects were two-way interactions involving task difficulty, the two-way interaction of each gender and SES with task difficulty was entered into the model as well. No other or higher-order interaction terms involving gender and SES were specified.

All models were estimated in the R environment (R Development Core Team, 2008) using the function lmer from the package lme4 (Bates et al., 2015), version 1.1-15. For better interpretability of regression coefficients, all metric variables were centered and standardized within each country or economy. This means that regression coefficients represent expected changes in the criterion variable in terms of its within-country standard deviation, per within-country standard deviation of each predictor. Standard deviations of all variables in the analyses did not vary much across countries (see Supplementary Material 1). Gender was entered dummy-coded with girls as the reference group.

#### Integration of Country-Specific Results

Country-specific results (fixed effects) were integrated using a random-effects meta-analytic model (Hedges and Vevea, 1998), using the R-package metafor (Viechtbauer, 2010). Meta-analysis lends itself for the analysis of data such as the present for multiple reasons. In educational assessments such as PISA, sampling occurs at the level of countries, so that an analysis pooling data from all countries would not be appropriate. However, besides effects for individual countries, it is of interest how an effect turns out in general, i.e., across countries. A random-effects meta-analytic model that discriminates a fixed (total) effect from a random, study-specific effect seems especially suitable in this situation: The fixed effect may be interpreted as a general effect, which is the same across countries. The variance of the study (i.e., country) specific effect gives an estimate, and allows a significance test, for the variance of county specifics adding to the total effect size, over and above sampling variance.^2^ To conduct the meta-analysis for each effect, one vector was created for each effect containing the country-specific estimates of each effect through reading the respective effect from the respective lmer object using the function fixef from the lme4 package. A second vector containing each effect’s standard error for each country was created using the se.fixef function from the package arm (Gelman and Su, 2016). These two vectors (after taking the square of each effect’s standard error to arrive at the variance) were given to the rma function from the metafor package. An alpha level of 0.05 was set for all significance tests.

#### Illustration of Interaction Effects Through Simple Slopes

To illustrate the interaction effects between task difficulty and comprehension skill, knowledge of reading strategies, and reading enjoyment, respectively, simple slopes were computed and tested for significance at the upper and lower boundaries of the respective distributions (2.5th and 97.5th percentiles, or ±1.96 standard deviations). For comprehension skill (and task difficulty) these percentiles represent the boundaries between the highest and second to highest competency level (levels 5 and 6), and the lowest and second to lowest competency level (levels 1a and 1b) respectively (see OECD, 2010, for the interpretation and description of reading competency levels). The values at which to compute simple slopes were chosen for knowledge of reading strategies and reading enjoyment in accordance. It is important to note that irrespective of the values chosen for the computation of simple slopes, the interaction effect as such relates to the whole sample, and simple slopes could, in principle be computed for *any* value of each predictor in the model (see Aiken et al., 2003).

## Results

Means, standard deviations and correlations for all variables in the analyses pooled across countries and economies are provided in Table 1. Country-specific statistics are provided in Supplementary Material 1.

**TABLE 1:** Means, standard deviations, and correlations for all variables in the study in their original metric and coding.

	**Min**	**Max**	***M***	**SD**	**Correlations**						
(1) Total time on task^a,b^	1.17	1753.42	104.21	86.23	(1)	(2)	(3)	(4)	(5)	(6)	(7)
(2) Time on relevant pages^a,b,c^	0.07	1753.42	42.57	46.59	0.42						
(3) Task difficulty^a^	–2.72	2.33	–0.01	1.04	0.38	0.21					
(4) Comprehension skill^d^	0.00	884.66	501.28	100.70	0.08	0.03	0.00				
(5) Strategy knowledge^d^	0.00	17.00	9.86	4.39	0.06	0.03	0.00	0.43			
(6) Reading enjoyment^d^	–3.23	3.49	0.02	0.98	0.06	0.04	0.00	0.39	0.27		
(7) Gender^d,e^	1.00	2.00	1.49	0.50	–0.04	–0.03	0.00	–0.17	–0.16	–0.28	
(8) SES^d^	–6.04	3.03	–0.06	0.99	–0.01	–0.03	0.00	0.33	0.22	0.13	0.01

*${}^{a}$*k* = 640,482 task responses. ${}^{b}$Seconds. ${}^{c}$Averaged across the number of relevant pages available. ${}^{d}$*n* = 32,699 students. ${}^{e}$1 = female, 2 = male.*

### Random Effects

There was significant variation of time on task, as well as time on relevant pages, between tasks, subjects, and schools in each country and economy. The corresponding variance components can be seen in detail in the model summaries that are provided as Supplementary Material 2. Supplementary Material 3 provides the respective significance tests. In the following, all estimates are meta-analytic fixed effects across countries and economies. Country-specific effects can be found in Supplementary Material 2. Most of the fixed effects of theoretical interest showed significant variability across countries and economies, over and above sampling variance. Since, however, this variability in the present research was not of theoretical interest, the estimates and the significance of between-country variance is presented as Supplementary Material 4. In the following, if a fixed effect showed *no* variance across countries over and above sampling variance (the exception from the rule), this is explicitly mentioned.

### Fixed Effects

#### Main Effects of Task Difficulty Comprehension Skill, Strategy Knowledge and Reading Enjoyment

As expected, there was a significant main effect of task difficulty, meaning that students on average took more time in harder tasks (meta-analytic effect: *b* = 0.39, *SE* = 0.02, 95%-CI: [0.35; 0.43]), and on average spent more time on task-relevant pages (meta-analytic effect: *b* = 0.18, *SE* = 0.03, 95%-CI: [0.13; 0.24]). Neither main effect of task difficulty varied across countries over and above sampling variance. Also, both time on task indicators were positively predicted by comprehension skill. More skilled comprehenders spent more time on the tasks in general, and they spent more time on relevant pages (meta-analytic effect for both time on task indicators: *b* = 0.09, *SE* = 0.01, 95%-CI: [0.08; 0.10]).

On top of the main effect for comprehension skill, there was a positive main effect of strategy knowledge on both time on task indicators. For both time on task indicators this effect was *b* = 0.03 (*SE* < 0.01), 95%-CI: [0.02; 0.03]. In addition to the main effects of comprehension skill and strategy knowledge, reading enjoyment had a positive main effect, meaning that students enjoying reading both spent more time on the tasks in total (meta-analytic effect: *b* = 0.02, *SE* < 0.01, 95%-CI: [0.01; 0.03]), and on relevant pages (meta-analytic effect: *b* = 0.02, *SE* < 0.01, 95%-CI: [0.01; 0.02]).

### Interactions of Task Difficulty With Comprehension Skill, Strategy Knowledge and Reading Enjoyment

#### Comprehension Skill

**FIGURE 3:**
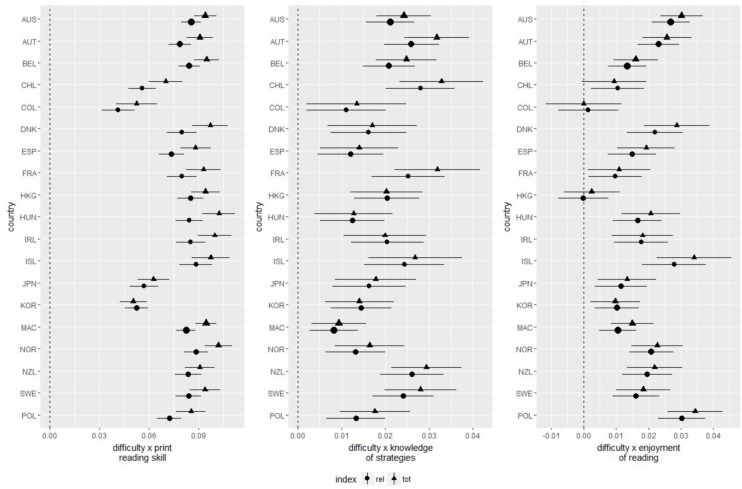
Interaction of task difficulty with comprehension skill, knowledge of reading strategies, and reading motivation as predictor of total time on task (tot) and average time on relevant hypertext pages (rel). Error bars indicate 95% confidence intervals. Symbol sizes are proportional to precision of each estimate.

**FIGURE 4:**
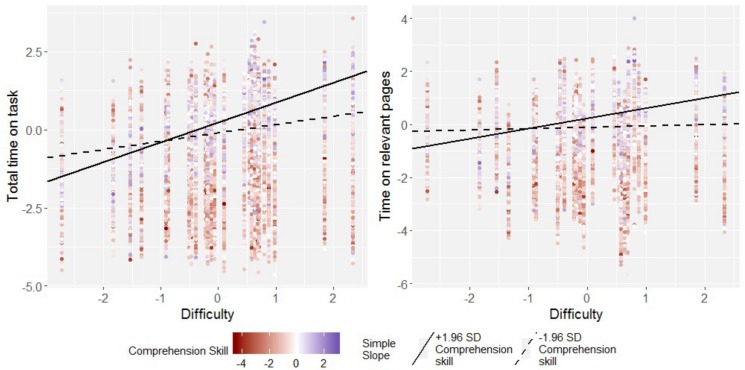
Simple slopes for the regression of total time on task and time on relevant pages on task difficulty in students high (97.5th perc.) and low (2.5th perc.) in comprehension skill for one sample country (Australia). Data points are raw data. Regression intercepts and slopes are model-based estimates.

The main effects of task difficulty and comprehension skill were qualified by a significant positive two-way interaction (see Figure 3, left panel, and Figure 4 for an illustration). Meta-analytically, this interaction amounted to *b* = 0.09 (*SE* < 0.01), 95%-CI: [0.08; 0.09] for total time on task, and *b* = 0.08 (*SE* < 0.01), 95%-CI: [0.07; 0.08] for time on relevant pages (see the left hand panel in Figure 3), representing a medium-sized effect each.

To further interpret these interactions, simple slopes were computed depicting the effect of task difficulty in very strong comprehenders and very weak comprehenders (*z*_comprehension_ = ±1.96, see Figure 4 for an illustration). Likewise, the effect of comprehension skills was estimated in very hard and very easy items (*z*_difficulty_ = ± 1.96). These analyses revealed the following: There was a strong effect of task difficulty on both time on task indicators in strong readers, amounting meta-analytically to *b* = 0.60 (*SE* = 0.02), 95%-CI: [0.52; 0.61] for total time on task, and to *b* = 0.33 (*SE* = 0.03), 95%-CI: [0.28; 0.39] for time on relevant pages. Both these slopes had no significant variance across countries. For poor comprehenders at the lower end of the comprehension skill distribution the effect of task difficulty on total time on task was much reduced, though still significant, the meta-analytical effect was *b* = 0.22 (*SE* = 0.02), 95%-CI: [0.18; 0.26]. No significant effect of task difficulty on time on relevant pages was found in poor comprehenders, *b* = 0.03 (*SE* = 0.03), 95%-CI: [−0.02; 0.09]. Once again, these two simple slopes displayed no variance over and above sampling variance.

Correspondingly, in hard tasks, there was a strong positive association of comprehension skill with both total time on task, *b* = 0.26 (*SE* = 0.01), 95%-CI: [0.23; 0.28], and time on relevant pages, *b* = 0.23 (*SE* = 0.01), 95%-CI: [0.22; 0.26]. In easy tasks, in contrast, this association was negative for both total time on task, *b* = −0.08 (*SE* = 0.01), 95%-CI: [−0.10; −0.07], and time on relevant pages, *b* = −0.06 (*SE* = 0.01), 95%-CI: [−0.07; −0.05].

Taken altogether, these results suggest the following: Skilled comprehenders align their total time on task, as well as the time they spend on task-relevant hypertext pages, closely to the tasks’ difficulties. In contrast, much less of such an adaptive behavior occurs in poor comprehenders. These readers show some alignment of their total time on task with task difficulty, but none of the time they spend on relevant parts of the text. Correspondingly, when tasks were hard, skilled comprehenders appeared to invest more time in these tasks than poor comprehenders. Easy tasks in contrast were more quickly solved by skilled, as opposed to poor comprehenders.

#### Knowledge of Reading Strategies

**FIGURE 5:**
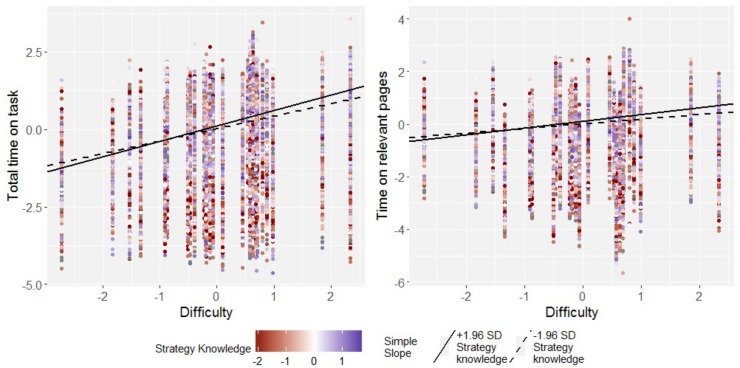
Simple slopes for the regression of total time on task and time on relevant pages on task difficulty in students high (97.5th perc.) and low (2.5th perc.) in knowledge of reading strategies for one sample country (Australia). Data points are raw data. Regression intercepts and slopes are model-based estimates.

The positive main effect of strategy knowledge on both total time on task and time on relevant pages was in each case qualified by a significant positive interaction with task difficulty, amounting to *b* = 0.02 (*SE* < 0.01), 95%-CI: [0.02; 0.02] both for total time on task and time on relevant pages (see the middle panel in Figure 3, and Figure 5 for an illustration), which represented a small effect each. To interpret theses interactions, simple slopes were computed to estimate the effect of task difficulty for students at the upper and lower ends of the strategy knowledge distribution (*z*_strategy knowledge_ = ± 1.96), and, correspondingly, the effect of strategy knowledge in easy and hard items. For students high in knowledge of reading strategies, the effect of task difficulty on total time on task was estimated as *b* = 0.44 (*SE* = 0.02), 95%-CI: [0.40; 0.47], and on time on relevant pages as *b* = 0.22 (*SE* = 0.03), 95%-CI: [0.16; 0.29]. Both these effects were homogeneous across countries and economies. For students low in knowledge of reading strategies, the effects of task difficulty on time on task were still significant, but reduced in magnitude. They amounted to *b* = 0.35 (*SE* = 0.02), 95%-CI: [0.31; 0.39] for total time on task and *b* = 0.15 (*SE* = 0.03), 95%-CI: [0.09; 0.21] for time on relevant pages. Once again, these two effects showed no variability over and above sampling variance across countries and economies.

In hard tasks, the effect of strategy knowledge on total time on task was estimated as *b* = 0.07 (*SE* = 0.01), 95%-CI: [0.06; 0.08], and the effect on time on relevant pages as *b* = 0.06 (*SE* < 0.01), 95%-CI: [0.05; 0.07]. These positive associations were reversed to negative in easy tasks, where the effect of strategy knowledge on total time on task was *b* = −0.02 (*SE* < 0.01), 95%-CI: [−0.01; −0.02], and on time on relevant pages *b* = −0.01 (*SE* < 0.01), 95%-CI: [−0.01; 0.00].

Taken together these results suggest that over and above the effect of comprehension skill, students with better knowledge of reading strategies do a better job in aligning their time on task behavior with task difficulty. Students with better knowledge of reading strategies at the same time invest more time in hard tasks, and are quicker in solving easy tasks, than their less knowledgeable peers.

#### Reading Enjoyment

**FIGURE 6:**
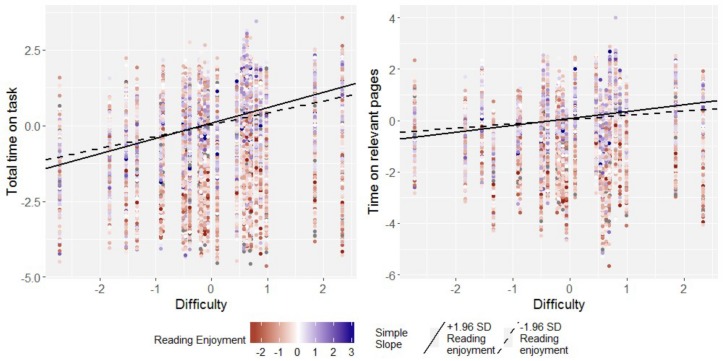
Simple slopes for the regression of total time on task and time on relevant pages on task difficulty in students high (97.5th perc.) and low (2.5th perc.) in enjoyment of reading for one sample country (Australia). Data points are raw data. Regression intercepts and slopes are model-based estimates.

As were the main effects of comprehension skill and knowledge of reading strategies, the main effect of reading enjoyment was moderated by task difficulty though a significant positive interaction, *b* = 0.02 (*SE* < 0.01), 95%-CI: [0.01; 0.02] for both total time on task and time on relevant pages (see the right hand panel in Figure 3, and Figure 6 for an illustration), which represented a small effect each. Simple slopes analyses (see Figure 6 for an illustration) revealed that in students high in reading enjoyment, there were strong or medium sized effects of task difficulty on both total time on task, *b* = 0.43 (*SE* = 0.02), 95%-CI: [0.39; 0.47], and time on relevant pages, *b* = 0.22 (*SE* = 0.03), 95%-CI: [0.16; 0.27]. These effects were reduced, but remained positive and significant in students low in reading enjoyment, where they amounted to *b* = 0.36 (*SE* = 0.02), 95%-CI: [0.32; 0.40] for total time on task, and *b* = 0.16 (*SE* = 0.03), 95%-CI: [0.09; 0.21]. All simple slopes for task difficulty in students low and high in reading enjoyment did not display variance over and above sampling variance.

As for comprehension skill and knowledge of reading strategies, a positive effect of enjoyment of reading was found in hard tasks, which amounted to *b* = 0.05 (SE = 0.01), 95%-CI: [0.04; 0.06] for both total time on task and time on relevant pages. In easy tasks, this effect was once again reversed to negative, and amounted to *b* = −0.02 (*SE* < 0.01), 95%-CI: [−0.03; 0.00] for total time on task, and *b* = −0.01 (*SE* < 0.01), 95%-CI: [−0.02; 0.00]. Thus, on top of the corresponding effects for comprehension skill and knowledge of reading strategies, students who enjoy reading more appear to invest more time in difficult tasks, but are quicker when they work on easy tasks than their peers who report less enjoyment in reading. It should be noted though that the negative effect of enjoyment was small, and that simple slopes were computed for tasks at the lower and upper end of the task difficulty distribution. Thus, for easy to moderately difficult tasks, the effect for enjoyment in reading on time on task would be zero, or slightly positive.

## Discussion

The present article examined the task-adaptive allocation of time, and time spent on relevant pages, while reading digital text, dependent on students’ comprehension skills, knowledge of reading strategies, and enjoyment of reading. Although these three student characteristics are positively correlated (see Table 1 and Supplementary Material 1), independent effects (that is, while controlling for each other) could be secured, indicating that students high in each of these variables showed a more pronounced adaptation, both of total time on task and of time on relevant pages, to the tasks’ difficulties. This was evidenced by significant positive interactions of each these student characteristics with task difficulty in predicting time on task and time on relevant pages, which were found consistently across 19 countries and economies (the only exception being Colombia and Hungary, where no interaction of task difficulty with reading enjoyment was found, see Figure 3).

### The Present Results Viewed From Previous Theory and Findings

These results are much in line with research from cognitive, educational, and social psychology that describes how students build models of the task when reading, how they monitor the reading process, and how they maintain effort when encountering a lengthy assessment comprising of multiple tasks, such as PISA. Specifically, the finding that time on task and task relevant pages are more positively predicted by task difficulty in strong comprehenders is much in line with the RESOLV model (Rouet et al., 2017), as strong comprehenders can be expected to be better in creating adequate task models. It is also in line with previous research pointing to better comprehenders behaving more task-adequate when it comes to selecting relevant, and discarding non-relevant text materials (Cerdán et al., 2011; Salmerón et al., 2015a). The result that knowledge of reading strategies is predictive of the adaptivity of time on task behavior also is in line with the RESLOV model, as well with earlier models of metacognitive engagement while learning, such as Winne and Hadwin’s (1998) COPES (Conditions, Operations, Procedures, Evaluations, Standards) model. Finally, the interaction of task difficulty with reading enjoyment is consistent with research describing position effects, or performance declines, in low-stakes assessments as a result of failing self-control and, as a result, motivation to mobilize mental resources (e.g., Lindner et al., 2017; Nagy et al., 2018a). These effects are moderated by students’ enjoyment of reading, presumably because these students view the assessment task as less aversive, and thus suffer less from failing self-control (Inzlicht and Schmeichel, 2012). From this perspective, it was to be expected, that students enjoying reading as an activity would also be more likely to invest time especially in hard tasks. This latter result is also nicely aligned with recent descriptions of “engaged” reading as proposed by Guthrie and colleagues (Guthrie et al., 2012). In their model, a direct predictor of reading achievement is behavioral engagement, which they also coin “dedication” (p. 604). Behavioral engagement in itself is dependent on motivations to read. In the present context, we might well assume behaviorally dedicated students especially those who allocate their time especially in hard tasks, and devote extra time especially to reading relevant parts of the text when the task is hard.

### Implications of the Present Results for Assessment and Education

As mentioned in the introductory part of this article, completion of an assessment task, and, in turn, the estimated ability of a student is not merely the reflection of a latent variable. Rather, it is always the result of intertwined cognitive and motivational processes carried out at time of task completion. One of these processes is the task-adequate mobilization of cognitive resources, and thus the expenditure of time. From the perspective of the present results thus the question arises whether time on task, or time spent on relevant pages, is governed by variables that can be regarded as part of the to-be-measured construct “digital reading skill”. In other words: If a crucial process of task engagement, that is predictive of task performance, is functionally dependent on processes and dispositions that are clearly outside the definition of the targeted construct, this would pose a threat to validity arguments made on the basis of the respective test scores (AERA et al., 2014). The largest interaction effects found in the present research were those of task difficulty with comprehension skill. Comprehension however clearly is part of the construct “digital reading”, as digital reading is reading in the first place. Thus, if a student is in a better position to solve a digital reading task due to better comprehension skill in part because these superior comprehension skills enable them to better align their effort with the task’s requirements, this does not necessarily pose a threat to the assessment’s validity. Rather, one might argue, it describes an additional pathway whereby good comprehension skills predict good performance in digital reading, and thus explain the positive correlation that is usually found between offline and online measures of reading skill and performance (e.g., Coiro, 2011; OECD, 2011; Naumann and Salmerón, 2016).

A similar argument might be made for knowledge of reading strategies. A long tradition of previous research has pointed to the necessity of strategic control especially in reading situations encountering digital text, web-based text, hypertext, or multiple texts (e.g., Bannert, 2003; Azevedo and Cromley, 2004; Naumann et al., 2008; see Cho and Afflerbarch, 2017 for an overview). If, however from a construct perspective metacognitive regulation is one central aspect of reading digital text, it would be counterintuitive to view it as a threat to validity when knowledge of reading strategies governs the adaptive allocation of time on task, and possibly thereby performance on tasks. Rather, as for comprehension skill, the present results evidence one particular mechanism by which knowledge of reading strategies might translate itself into successful reading of digital text.

This notion does not necessarily hold for enjoyment of reading. According to the reasoning put forward in the present research, students high in reading enjoyment do a better job in aligning their time on task behavior with task difficulty because they see reading as less an aversive task. For this reason, it is easier for them than for their peers lower in reading enjoyment to maintain effort and invest time in difficult tasks. Thus, according to the present reasoning, the positive association of reading enjoyment, or reading motivation in general, and reading skill, does not only arise because students higher in reading enjoyment, or motivation, come from higher SES backgrounds, from where they also can acquire better skill (e.g., Artelt et al., 2010). Also, it is not (only) that higher enjoyment or motivation longitudinally bring about better skills, or the reverse (e.g., Becker et al., 2010; Retelsdorf et al., 2011). Rather, just like comprehension skill and knowledge of reading strategies, reading enjoyment seems to be among the variables that govern the process of task engagement in the assessment situation itself and thereby may bring about better task performance and thus a higher level of estimated skill.

Other than comprehension skill and knowledge of reading strategies however, reading enjoyment is not necessarily to be seen as a part of the construct “skill in reading digital text”. In other words: A skilled digital reader, who is not in command of comprehension skills is as self-contradictory an idea as a skilled digital reader, who is not in possession of knowledge of reading strategies. In contrast to this, a skilled digital reader who simply does not enjoy reading might be a rare observation, as reading skill and enjoyment are usually positively correlated. The notion of such a reader, however, is not at all a contradictory idea.

From these perspectives, practical implications for the design of assessments, and practical implications for reading in other task-oriented reading situations such as learning are not quite aligned with one another: The finding that reading enjoyment, even if to only a small extent, enhances the adaptive allocation of time might pose a threat to valid interpretations of test scores. On the other hand, it once again highlights the crucial role of motivation in bringing about dedicated and engaged reading behavior, which in turn has been found to be a crucial determinant of learning from text (Guthrie et al., 2012, 2013). This, in turn, once again highlights the need for students to develop motivational traits and attitudes that help them to put in the effort required to cope with difficult and demanding digital texts. Obviously, this notion holds also for knowledge of reading strategies, and, last not least, comprehension skills. Putting students in a position to adequately mobilize cognitive resources when dealing with digital text seems especially important, as digital text to an increased degree requires students not only to “navigate” (see section “The present research” above), but also to evaluate text (Salmerón et al., 2018), a process which is cognitively demanding (Richter and Maier, 2017), and which many students find difficult to perform (e.g., Brante and Strømsø, 2018).

### Limitations and Directions

Obviously, the interpretations of the present results put forward here are not without alternative. This is a result of the correlational nature of many large-scale assessment data sets, the present amongst them. This means that there is a host of person-related variables that might, in theory, account for the present results but were unaccounted for in the present research. One candidate here is for example dispositional, or trait self-control, a variable that was found to be related to test-taking effort (Lindner et al., 2017), and thus may very well predict how well students are prepared to align their time on task-behavior to task difficulties. Another variable not taken into account here are specifics of students’ preparedness to cope with digital text, such as their navigation skills. Against the background of navigation being a central requirement of reading digital text (Salmerón et al., 2018), students’ preparedness to cope with navigation demands might also govern how much time they are prepared to invest in hard, and how little time they might need to complete easy digital reading tasks. Future research thus should seek out additional variables that might affect students’ preparedness to adapt their time on task behavior. Analyses such as these might also explain why some lesser skilled readers in fact did align their time on task behavior with task difficulties, while others did not (see Figure 4): Perhaps some poorer comprehenders are in possession of other skills than comprehension, which compensate for their lesser comprehension skill, allowing them to nevertheless building an adequate task model. For instance, recent research has shown that problem solving skills interact with comprehension skills in predicting digital reading in such a compensatory fashion (Naumann et al., 2018).

A second limitation comes from the fact that the three predictors used in the present analyses were measured with largely varying numbers of items (although the reliabilities were comparable). Thus, in an assessment using a more comprehensive measurement of reading enjoyment, or knowledge of reading strategies, the interactions of these variables with task difficulty might have been even stronger, maybe at the expense of the interaction between comprehension skill and task difficulty. Future research will have to seek out whether the small effect size for the interaction between reading enjoyment and task difficulty is indeed a function of the comparatively small number of items, or if – to the contrary – after controlling for comprehension skill, there is little variance left to be explained for reading enjoyment due to these variables being positively correlated (see Table 1 and Supplementary Material 1).

In a similar vein, future research should overcome not only the limited number of items, but also the limited operationalization of reading motivation used in the present research. For example, in real-life task oriented reading it might well be the case that topic interest is even more important than reading enjoyment in shaping the interaction between task difficulty and time on task: It might well be that a person who only moderately enjoys reading (and might even be a modest comprehender) will invest time even in a hard task if they have a very high interest in the topic. Research such as this however must be left to future experiments, as large-scale reading assessments usually cannot provide data on topic interest due to the variety of topics addressed by the texts in the assessment. Finally, future research into the role of motivational variables might consider not only linear (as in the present research), but also more complex non-linear effects. A motivated reader for example, who however is in possession of only moderate comprehension skills, might adapt time on task behavior to task difficulty in a non-linear fashion. Such a reader might invest time especially in moderately difficult tasks, while realizing that very hard tasks are beyond their skill level.

A third limitation, and possible avenue for future research, comes from the fact that only one domain was investigated in this research. Future studies might look at how e.g., the time on task behavior in mathematics might be shaped by students’ mathematical skills. For example, the ability to “formulate” a mathematical problem, i.e., to “translate from a real-world setting to the domain of mathematics and provide the real-world problem with mathematical structure, representations, and specificity” (OECD, 2013, p. 28) might be conceptually related to building an adequate task model in a reading task. Also, subjective interest in mathematics might moderate the task difficulty-time on task relationship in a fashion similar to the respective effects of reading enjoyment that were found in the present research. With reference to tasks, requirements in the present research were operationalized as the tasks’ overall difficulties, as estimated by the international calibration of the Digital Reading Assessment items (OECD, 2012). Building on the present results, future research might seek out which specific features of a digital reading task that might make it “hard” (on the word, sentence, text, or intertextual level) in particular drives time on task behavior in conjunction with person level variables such as the ones addressed here. From an analysis such as this, the question might also be addressed how digital reading assessment tasks might be constructed in a way that variables such as reading enjoyment, or other person level variables that are not part of the targeted construct, do not interact with task features in bringing about task engagement processes that presumably impact task performance and thus estimated abilities. With large scale assessments such as TIMSS or PISA moving toward being computer-based in general (Mullis, 2017; OECD, 2017), analyses such as these could be carried out routinely as part of field trials, and thereby potentially increase the validity of the assessments and in turn the veridicality of conclusions drawn for educational policy and practice.

## Ethics Statement

This study is based on a secondary analysis of OECD PISA 2009 data. Data collection was in accordance with APA ethical standards. The author had OECD’s permission to utilize the PISA 2009 Digital Reading Data log files, where not publicly available, for scholarly research and thanks the OECD for this permission.

## Author Contributions

JN helped in conceiving the assessment materials, conceived and conducted the analyses, and wrote the manuscript.

## Conflict of Interest Statement

The author declares that the research was conducted in the absence of any commercial or financial relationships that could be construed as a potential conflict of interest.

## Footnotes

1 Note that in PISA 2009, two separate indices were built on the basis of the two scenarios. In the present research, the two scenarios were combined into one score in accordance with the intentions of the authors of the original instrument from where idea of measuring strategy knowledge employed in PISA 2009, as well as the scenarios and to-be-rated strategies were derived (see Artelt et al., 2009).

2 Theoretically, an alternative to the meta-analytic approach used here would have been a model where country is treated as a random effect, and a random slope is estimated for each effect across countries. However, apart from the fact that given the number of fixed effects in the present analysis, such a model would probably would have been computationally intractable, it would only tell us *if* an effect varies across countries, but not in which way. Including country as another fixed effect, and estimating its interaction with each of the other fixed effects in the analysis would have added at least another 20 fixed effects to an already complex model. Thus, in the present case, the meta-analytic approach appeared to be the best compromise between comprehensiveness and parsimony that could be found.
