# Supplementary material for: The Skilled, the Knowledgeable, and the Motivated: Investigating the Strategic Allocation of Time on Task in a Computer-Based Assessment
Source: Front Psychol. 2019 Jun 27;10:1429. doi: 10.3389/fpsyg.2019.01429 (PMC6660318; doi:10.3389/fpsyg.2019.01429)
Supplement: Supplementary file 1 [file Data_Sheet_1.pdf]

Supplement 1 to 'The Skilled, the Knowledgeabeable, and the Motivated: Investigating the strategic allocation of time on task in a computer-based assessment'

Johannes Naumann

June 2019

Country-specific descriptives

This supplement presents country-specific descriptive statistics (see manuscript for pooled statistics). All variables are in their original metric. Note that for the analysis, all metric variables were z-standardized per country, and gender was dummy-coded with girls as the reference group. The last eight columns in each table represent correlations.

Total time on task, time on relevant pages and task difficulty were measured on the task level. All other variables were measured on the student level. Please refer to the manuscript as to how each of these variables was measured. Please note that means, standard deviations and correlations are not directly comparable to those reported by OECD (e.g. 2010; 2011), as Weighted Maximum Likelihood estimates (WLEs) were employed for Comprehension Skill rather than Plausible Values (PVs), and no student weights were employed. Number of students (N) and numberof item responses (k) are given for each country in the table notes.

Variable names are to be read as follows:

Dependent variables:

Tot. time: Total time on task
Rel. time: Average time on relevant pages

Predictors:

Difficulty: Task difficulty
Compr. skill: Comprehension skill
Strat. know.: Knowledge of reading strategies
Enjoyment: Reading enjoyment

Control variables:

Gender: Gender, Boys = 2, Girls = 1
SES: Socio-economic status (ESCS)

Descriptive statistics per country

AUSTRALIA

	Min	Max	M	SD	1.	2.	3.	4.	5.	6.	7.	8.
1. Tot. time	1.30	1510.30	96.20	78.65	1.00							
2. Rel. time	0.09	1361.15	38.71	39.48	0.37	1.00						
3. Difficulty	-2.72	2.33	0.00	1.04	0.42	0.24	1.00					
4. Compr. skill	19.57	866.12	517.07	106.63	0.08	0.05	0.00	1.00				
5. Strat. know.	0.00	17.00	9.78	4.43	0.06	0.04	0.00	0.46	1.00			
6. Enjoyment	-3.23	3.49	0.01	1.12	0.05	0.03	0.00	0.46	0.36	1.00		
7. Gender	1.00	2.00	1.48	0.50	-0.04	-0.03	0.00	-0.17	-0.18	-0.27	1.00	
8. SES	-2.75	2.51	0.37	0.74	0.02	0.01	0.00	0.35	0.25	0.24	0.00	1.00

Note: N = 2800, k = 53607

AUSTRIA

	Min	Max	M	SD	1.	2.	3.	4.	5.	6.	7.	8.
1. Tot. time	1.24	1496.77	90.50	74.85	1.00							
2. Rel. time	0.09	515.58	37.50	40.11	0.42	1.00						
3. Difficulty	-2.72	2.33	0.00	1.04	0.32	0.15	1.00					
4. Compr. skill	14.18	815.02	471.06	102.99	0.16	0.11	0.00	1.00				
5. Strat. know.	0.00	17.00	10.33	4.45	0.12	0.08	0.00	0.49	1.00			
6. Enjoyment	-3.23	3.49	-0.17	1.19	0.10	0.07	0.00	0.43	0.30	1.00		
7. Gender	1.00	2.00	1.49	0.50	-0.04	-0.03	0.00	-0.21	-0.22	-0.34	1.00	
8. SES	-3.11	2.73	0.07	0.80	0.07	0.04	0.00	0.32	0.19	0.21	0.04	1.00

Note: N = 2483, k = 47326

BELGIUM

	Min	Max	M	SD	1.	2.	3.	4.	5.	6.	7.	8.
1. Tot. time	1.17	1234.38	101.91	78.48	1.00							
2. Rel. time	0.07	830.12	41.45	40.86	0.35	1.00						
3. Difficulty	-2.72	2.33	0.00	1.04	0.41	0.22	1.00					
4. Compr. skill	115.56	869.40	521.55	102.49	0.10	0.06	0.00	1.00				
5. Strat. know.	0.00	17.00	11.06	4.37	0.08	0.05	0.00	0.55	1.00			
6. Enjoyment	-3.23	3.49	-0.14	1.02	0.07	0.05	0.00	0.43	0.36	1.00		
7. Gender	1.00	2.00	1.50	0.50	-0.01	-0.01	0.00	-0.12	-0.14	-0.29	1.00	
8. SES	-5.71	2.57	0.24	0.92	0.06	0.04	0.00	0.38	0.28	0.23	0.05	1.00

Note: N = 2681, k = 51096

CHILE

	Min	Max	M	SD	1.	2.	3.	4.	5.	6.	7.	8.
1. Tot. time	1.47	1217.51	123.11	94.87	1.00							
2. Rel. time	0.10	965.99	53.45	57.48	0.47	1.00						
3. Difficulty	-2.72	2.33	-0.01	1.04	0.33	0.17	1.00					
4. Compr. skill	146.90	793.84	457.89	88.49	0.06	0.00	0.00	1.00				
5. Strat. know.	0.00	17.00	9.44	4.28	0.05	0.01	0.00	0.45	1.00			
6. Enjoyment	-3.23	3.49	-0.05	0.81	0.03	0.01	0.00	0.24	0.17	1.00		
7. Gender	1.00	2.00	1.50	0.50	-0.02	-0.02	0.01	-0.12	-0.10	-0.31	1.00	
8. SES	-3.83	2.58	-0.46	1.20	-0.02	-0.05	0.01	0.43	0.31	0.11	-0.01	1.00

Note: N = 1638, k = 28924

COLOMBIA

	Min	Max	M	SD	1.	2.	3.	4.	5.	6.	7.	8.
1. Tot. time	2.29	1753.42	130.47	109.20	1.00							
2. Rel. time	0.19	1753.42	58.79	68.81	0.52	1.00						
3. Difficulty	-2.72	2.33	-0.01	1.03	0.26	0.13	1.00					
4. Compr. skill	107.76	789.87	434.45	93.98	0.03	-0.03	0.00	1.00				
5. Strat. know.	0.00	17.00	8.42	4.50	0.04	0.00	0.00	0.46	1.00			
6. Enjoyment	-3.23	3.49	0.16	0.69	0.05	0.04	0.00	0.17	0.17	1.00		
7. Gender	1.00	2.00	1.49	0.50	-0.03	-0.03	0.00	-0.02	-0.02	-0.20	1.00	
8. SES	-4.65	1.92	-1.07	1.28	-0.02	-0.05	0.01	0.39	0.29	0.01	0.06	1.00

Note: N = 1345, k = 23017

DENMARK

	Min	Max	M	SD	1.	2.	3.	4.	5.	6.	7.	8.
1. Tot. time	1.49	1573.96	94.07	74.62	1.00							
2. Rel. time	0.12	406.37	37.75	37.04	0.35	1.00						
3. Difficulty	-2.72	2.33	0.00	1.04	0.35	0.18	1.00					
4. Compr. skill	19.57	790.16	484.72	93.27	0.12	0.07	0.00	1.00				
5. Strat. know.	0.00	17.00	10.56	4.33	0.09	0.05	0.00	0.45	1.00			
6. Enjoyment	-3.23	3.49	-0.04	0.86	0.08	0.05	0.01	0.37	0.33	1.00		
7. Gender	1.00	2.00	1.49	0.50	-0.05	-0.03	0.00	-0.14	-0.21	-0.28	1.00	
8. SES	-2.90	2.78	0.15	0.92	0.03	0.01	0.00	0.37	0.22	0.22	0.08	1.00

Note: N = 1204, k = 23011

SPAIN

	Min	Max	M	SD	1.	2.	3.	4.	5.	6.	7.	8.
1. Tot. time	1.70	1100.36	101.79	77.44	1.00							
2. Rel. time	0.13	440.54	41.41	39.79	0.35	1.00						
3. Difficulty	-2.72	2.33	-0.01	1.04	0.35	0.18	1.00					
4. Compr. skill	162.60	811.21	488.23	93.25	0.11	0.06	0.00	1.00				
5. Strat. know.	0.00	17.00	10.57	4.05	0.09	0.05	0.00	0.42	1.00			
6. Enjoyment	-3.23	3.49	-0.01	0.97	0.05	0.03	0.00	0.38	0.29	1.00		
7. Gender	1.00	2.00	1.50	0.50	-0.03	-0.03	0.00	-0.15	-0.20	-0.30	1.00	
8. SES	-3.08	2.69	-0.26	1.07	0.04	0.02	0.00	0.33	0.19	0.20	-0.01	1.00

Note: N = 1649, k = 31294

FRANCE

	Min	Max	M	SD	1.	2.	3.	4.	5.	6.	7.	8.
1. Tot. time	1.37	949.46	110.41	84.43	1.00							
2. Rel. time	0.13	699.96	46.04	46.78	0.38	1.00						
3. Difficulty	-2.72	2.33	-0.01	1.04	0.40	0.22	1.00					
4. Compr. skill	94.00	866.12	509.44	104.29	0.06	0.03	0.00	1.00				
5. Strat. know.	0.00	17.00	11.01	4.10	0.04	0.02	0.00	0.43	1.00			
6. Enjoyment	-3.23	3.49	0.05	1.02	0.05	0.03	0.00	0.43	0.30	1.00		
7. Gender	1.00	2.00	1.48	0.50	-0.02	-0.01	0.00	-0.16	-0.14	-0.25	1.00	
8. SES	-3.18	2.83	-0.09	0.82	0.02	0.00	0.00	0.36	0.19	0.20	0.04	1.00

Note: N = 1229, k = 23164

HONG KONG-CHINA

	Min	Max	M	SD	1.	2.	3.	4.	5.	6.	7.	8.
1. Tot. time	1.34	1322.16	109.18	108.64	1.00							
2. Rel. time	0.15	1294.65	45.03	65.60	0.54	1.00						
3. Difficulty	-2.72	2.33	-0.01	1.04	0.44	0.27	1.00					
4. Compr. skill	97.60	840.04	534.92	91.67	0.09	0.06	0.00	1.00				
5. Strat. know.	0.00	17.00	8.23	4.45	0.05	0.03	0.00	0.32	1.00			
6. Enjoyment	-3.23	3.49	0.33	0.72	0.03	0.03	-0.01	0.36	0.25	1.00		
7. Gender	1.00	2.00	1.53	0.50	-0.03	-0.03	0.00	-0.19	-0.11	-0.28	1.00	
8. SES	-3.93	1.92	-0.80	1.03	0.01	0.00	0.00	0.22	0.12	0.19	-0.03	1.00

Note: N = 1414, k = 26363

HUNGARY

	Min	Max	M	SD	1.	2.	3.	4.	5.	6.	7.	8.
1. Tot. time	1.64	1191.89	95.56	73.55	1.00							
2. Rel. time	0.14	443.22	39.82	39.85	0.37	1.00						
3. Difficulty	-2.72	2.33	-0.01	1.04	0.32	0.15	1.00					
4. Compr. skill	127.42	836.44	491.98	95.70	0.17	0.08	0.01	1.00				
5. Strat. know.	0.00	17.00	10.01	4.21	0.11	0.06	0.01	0.47	1.00			
6. Enjoyment	-3.23	3.49	0.11	0.88	0.09	0.05	0.00	0.41	0.34	1.00		
7. Gender	1.00	2.00	1.50	0.50	-0.05	-0.04	0.00	-0.22	-0.20	-0.33	1.00	
8. SES	-3.53	2.93	-0.23	0.96	0.07	0.02	0.00	0.47	0.22	0.22	0.05	1.00

Note: N = 1697, k = 32296

IRELAND

	Min	Max	M	SD	1.	2.	3.	4.	5.	6.	7.	8.
1. Tot. time	1.67	1341.25	104.54	88.54	1.00							
2. Rel. time	0.18	884.33	41.91	43.32	0.37	1.00						
3. Difficulty	-2.72	2.33	-0.01	1.04	0.40	0.24	1.00					
4. Compr. skill	32.71	847.83	499.99	99.61	0.07	0.04	0.00	1.00				
5. Strat. know.	0.00	17.00	10.59	4.07	0.04	0.03	-0.01	0.41	1.00			
6. Enjoyment	-3.23	3.49	-0.11	1.01	0.02	0.00	0.00	0.42	0.28	1.00		
7. Gender	1.00	2.00	1.50	0.50	-0.04	-0.03	0.00	-0.18	-0.15	-0.21	1.00	
8. SES	-3.22	2.86	0.04	0.84	0.01	0.00	0.00	0.27	0.15	0.24	0.03	1.00

Note: N = 1336, k = 25383

ICELAND

	Min	Max	M	SD	1.	2.	3.	4.	5.	6.	7.	8.
1. Tot. time	1.44	886.42	97.32	76.44	1.00							
2. Rel. time	0.15	501.09	38.36	38.39	0.38	1.00						
3. Difficulty	-2.72	2.33	-0.01	1.04	0.35	0.21	1.00					
4. Compr. skill	120.71	884.66	510.65	95.63	0.12	0.08	0.00	1.00				
5. Strat. know.	0.00	17.00	9.15	4.31	0.10	0.08	0.00	0.40	1.00			
6. Enjoyment	-3.23	3.49	-0.02	1.05	0.08	0.06	0.01	0.41	0.31	1.00		
7. Gender	1.00	2.00	1.45	0.50	-0.08	-0.06	0.00	-0.24	-0.25	-0.35	1.00	
8. SES	-3.09	3.03	0.58	0.88	0.03	0.02	0.00	0.26	0.16	0.16	0.01	1.00

Note: N = 930, k = 17706

JAPAN

	Min	Max	M	SD	1.	2.	3.	4.	5.	6.	7.	8.
1. Tot. time	1.82	1032.25	114.56	101.28	1.00							
2. Rel. time	0.18	638.69	44.82	51.89	0.44	1.00						
3. Difficulty	-2.72	2.33	-0.01	1.04	0.47	0.31	1.00					
4. Compr. skill	0.00	790.16	536.18	93.82	0.07	0.04	0.00	1.00				
5. Strat. know.	0.00	17.00	10.99	3.84	0.05	0.03	0.00	0.42	1.00			
6. Enjoyment	-3.23	3.49	0.34	1.03	0.03	0.02	0.00	0.32	0.23	1.00		
7. Gender	1.00	2.00	1.50	0.50	-0.01	-0.01	0.00	-0.11	-0.11	-0.17	1.00	
8. SES	-2.25	2.36	-0.01	0.74	0.01	0.00	0.00	0.21	0.12	0.15	0.04	1.00

Note: N = 1155, k = 21591

KOREA

	Min	Max	M	SD	1.	2.	3.	4.	5.	6.	7.	8.
1. Tot. time	1.59	1089.70	105.47	86.26	1.00							
2. Rel. time	0.17	535.28	41.02	42.52	0.36	1.00						
3. Difficulty	-2.72	2.33	0.00	1.04	0.46	0.29	1.00					
4. Compr. skill	260.00	833.08	542.83	82.83	0.06	0.03	0.01	1.00				
5. Strat. know.	0.00	17.00	10.25	4.39	0.03	0.01	0.00	0.47	1.00			
6. Enjoyment	-3.23	3.49	0.14	0.81	0.02	0.01	0.00	0.36	0.22	1.00		
7. Gender	1.00	2.00	1.51	0.50	-0.02	-0.01	0.00	-0.15	-0.16	-0.18	1.00	
8. SES	-3.41	2.38	-0.13	0.81	0.01	0.00	0.01	0.29	0.22	0.18	0.00	1.00

Note: N = 1452, k = 27747

MACAO-CHINA

	Min	Max	M	SD	1.	2.	3.	4.	5.	6.	7.	8.
1. Tot. time	1.40	1309.48	120.98	103.35	1.00							
2. Rel. time	0.15	866.28	48.91	59.99	0.48	1.00						
3. Difficulty	-2.72	2.33	-0.01	1.03	0.45	0.28	1.00					
4. Compr. skill	81.48	830.39	480.64	89.14	0.05	0.04	0.00	1.00				
5. Strat. know.	0.00	17.00	9.03	4.20	0.04	0.04	0.00	0.30	1.00			
6. Enjoyment	-3.23	3.49	0.09	0.70	0.04	0.03	0.00	0.32	0.25	1.00		
7. Gender	1.00	2.00	1.48	0.50	-0.05	-0.05	0.00	-0.24	-0.20	-0.28	1.00	
8. SES	-3.13	2.15	-0.61	0.90	0.02	0.02	0.00	0.13	0.13	0.18	-0.07	1.00

Note: N = 2484, k = 45159

NORWAY

	Min	Max	M	SD	1.	2.	3.	4.	5.	6.	7.	8.
1. Tot. time	1.39	959.13	96.97	77.65	1.00							
2. Rel. time	0.17	564.04	38.48	38.57	0.38	1.00						
3. Difficulty	-2.72	2.33	0.00	1.04	0.35	0.21	1.00					
4. Compr. skill	80.88	830.45	507.83	96.93	0.14	0.09	-0.01	1.00				
5. Strat. know.	0.00	17.00	9.52	4.35	0.09	0.07	-0.01	0.45	1.00			
6. Enjoyment	-3.23	3.49	-0.14	1.02	0.10	0.07	0.00	0.43	0.28	1.00		
7. Gender	1.00	2.00	1.51	0.50	-0.06	-0.04	0.00	-0.24	-0.21	-0.31	1.00	
8. SES	-2.83	2.54	0.47	0.74	0.03	0.01	0.00	0.26	0.16	0.16	0.02	1.00

Note: N = 1902, k = 36225

NEW ZEALND

	Min	Max	M	SD	1.	2.	3.	4.	5.	6.	7.	8.
1. Tot. time	1.17	1076.31	105.90	86.41	1.00							
2. Rel. time	0.07	573.16	42.17	42.36	0.37	1.00						
3. Difficulty	-2.72	2.33	-0.01	1.04	0.43	0.26	1.00					
4. Compr. skill	98.51	869.40	532.01	103.62	0.04	0.01	0.00	1.00				
5. Strat. know.	0.00	17.00	9.56	4.41	0.05	0.03	0.00	0.46	1.00			
6. Enjoyment	-3.23	3.49	0.19	1.01	0.05	0.03	0.00	0.49	0.31	1.00		
7. Gender	1.00	2.00	1.49	0.50	-0.04	-0.03	0.00	-0.19	-0.18	-0.33	1.00	
8. SES	-2.80	2.71	0.11	0.78	0.01	0.00	-0.01	0.37	0.23	0.23	0.03	1.00

Note: N = 1687, k = 32018

SWEDEN

	Min	Max	M	SD	1.	2.	3.	4.	5.	6.	7.	8.
1. Tot. time	1.54	1058.26	102.76	80.37	1.00							
2. Rel. time	0.16	859.62	41.03	40.65	0.35	1.00						
3. Difficulty	-2.72	2.33	-0.01	1.04	0.37	0.22	1.00					
4. Compr. skill	94.00	834.05	506.18	102.77	0.11	0.06	0.00	1.00				
5. Strat. know.	0.00	17.00	9.24	4.63	0.09	0.06	0.00	0.49	1.00			
6. Enjoyment	-3.23	3.49	-0.07	0.99	0.08	0.06	0.00	0.45	0.34	1.00		
7. Gender	1.00	2.00	1.48	0.50	-0.05	-0.03	0.00	-0.22	-0.19	-0.39	1.00	
8. SES	-6.04	2.59	0.36	0.81	0.05	0.03	0.00	0.37	0.22	0.20	-0.01	1.00

Note: N = 1760, k = 33405

POLAND

	Min	Max	M	SD	1.	2.	3.	4.	5.	6.	7.	8.
1. Tot. time	1.39	1655.90	96.35	76.76	1.00							
2. Rel. time	0.09	513.78	40.24	41.69	0.39	1.00						
3. Difficulty	-2.72	2.33	0.00	1.04	0.36	0.18	1.00					
4. Compr. skill	129.88	836.50	505.61	94.21	0.11	0.06	0.00	1.00				
5. Strat. know.	0.00	17.00	9.37	4.28	0.07	0.04	0.00	0.41	1.00			
6. Enjoyment	-3.23	3.49	0.03	1.08	0.06	0.04	0.00	0.38	0.27	1.00		
7. Gender	1.00	2.00	1.50	0.50	-0.03	-0.03	0.00	-0.25	-0.15	-0.34	1.00	
8. SES	-3.20	2.31	-0.21	0.91	0.04	0.00	0.00	0.37	0.21	0.22	0.05	1.00

Note: N = 1823, k = 34848
