# Supplementary material for: The Skilled, the Knowledgeable, and the Motivated: Investigating the Strategic Allocation of Time on Task in a Computer-Based Assessment
Source: Front Psychol. 2019 Jun 27;10:1429. doi: 10.3389/fpsyg.2019.01429 (PMC6660318; doi:10.3389/fpsyg.2019.01429)
Supplement: Supplementary file 2 [file Data_Sheet_2.pdf]

Supplement 2 to 'The Skilled, the Knowledgeabeable, and the Motivated: Investigating the strategic allocation of time on task in a computer-based assessment'

Johannes Naumann

June 2019

Country-specific estimates of the LMMs

This supplement presents country-specific estimates of the fixed and random effects of the LMMs. Variable names are to be read as follows:

Dependent variables:

z_tottime: Total time on task, logarithmised and standardized
z_reltime: Average time on relevant pages, logarithmised and standardized

Variables with estimated fixed effects

Predictors:

z_wler: Comprehension skill (WLEs), standardized
z_joy: Enjoyment of reading, standardized
z_meta: Knowledge of reading strategies, standardized
logits: Task difficulty, standardized

Control variables:

gender: Gender, Boys = 1, Girls = 0
z_escs: Socio-economic status (ESCS), standardized

Variables with estimated random intercepts

user: Identification variable for students
task: Identification variable for tasks (items)
schoolID: Identification variable for schools

Correlations between fixed effects are not displayed for reasons of space, they are available from the author at j.naumann@uni-wuppertal.de upon request.

Dependent variable total time on task

AUSTRALIA

```r
summary(time.dep.r[[1]])
```

```
## Linear mixed model fit by REML ['lmerMod']
## Formula: z_tottime ~ logits * z_wler * z_joy * z_meta + logits * z_escs +
##     logits * gender + (1 | user) + (1 | task) + (1 | schoolID)
##    Data: totdata
##
## REML criterion at convergence: 107971.8
##
## Scaled residuals:
##     Min      1Q  Median      3Q     Max
## -7.1168 -0.4711  0.0400  0.5527  7.8092
##
## Random effects:
##  Groups   Name        Variance Std.Dev.
##  user     (Intercept) 0.11544  0.3398
##  schoolID (Intercept) 0.03264  0.1807
##  task     (Intercept) 0.21667  0.4655
##  Residual             0.39238  0.6264
## Number of obs: 53607, groups:  user, 2800; schoolID, 334; task, 29
##
## Fixed effects:
##                              Estimate Std. Error t value
## (Intercept)                  0.069148   0.087741   0.788
## logits                       0.449945   0.082842   5.431
## z_wler                       0.084800   0.009621   8.814
## z_joy                        0.013713   0.009254   1.482
## z_meta                       0.022817   0.008910   2.561
## z_escs                      -0.009241   0.008335  -1.109
## gender                      -0.049299   0.016000  -3.081
## logits:z_wler                0.094078   0.003387  27.780
## logits:z_joy                 0.030132   0.003331   9.045
## z_wler:z_joy                -0.041197   0.008605  -4.788
## logits:z_meta                0.024260   0.003175   7.640
## z_wler:z_meta               -0.038372   0.008735  -4.393
## z_joy:z_meta                 0.003502   0.008933   0.392
## logits:z_escs                0.006762   0.002856   2.367
## logits:gender               -0.036527   0.005499  -6.643
## logits:z_wler:z_joy         -0.007493   0.003089  -2.426
## logits:z_wler:z_meta        -0.003014   0.003141  -0.959
## logits:z_joy:z_meta         -0.002438   0.003230  -0.755
## z_wler:z_joy:z_meta          0.006833   0.007463   0.916
## logits:z_wler:z_joy:z_meta -0.010717   0.002663  -4.024
##
## Correlation matrix not shown by default, as p = 20 > 12.
## Use print(x, correlation=TRUE)  or
##   vcov(x)     if you need it
```

AUSTRIA

```r
summary(time.dep.r[[2]])
```

```
## Linear mixed model fit by REML ['lmerMod']
## Formula: z_tottime ~ logits * z_wler * z_joy * z_meta + logits * z_escs +
##     logits * gender + (1 | user) + (1 | task) + (1 | schoolID)
##    Data: totdata
##
## REML criterion at convergence: 104658.1
##
## Scaled residuals:
##     Min      1Q  Median      3Q     Max
## -5.7594 -0.4517  0.0556  0.5566  5.8852
##
## Random effects:
##  Groups   Name        Variance Std.Dev.
##  user     (Intercept) 0.09073  0.3012
##  schoolID (Intercept) 0.13924  0.3732
##  task     (Intercept) 0.13958  0.3736
##  Residual             0.48397  0.6957
## Number of obs: 47326, groups:  user, 2483; schoolID, 253; task, 29
##
## Fixed effects:
##                             Estimate Std. Error t value
## (Intercept)                0.0364699  0.0743570   0.490
## logits                     0.2754965  0.0665924   4.137
## z_wler                     0.1088045  0.0105535  10.310
## z_joy                      0.0382556  0.0092490   4.136
## z_meta                     0.0420400  0.0090564   4.642
## z_escs                    -0.0003600  0.0082439  -0.044
## gender                     0.0099502  0.0172756   0.576
## logits:z_wler              0.0908708  0.0040041  22.694
## logits:z_joy               0.0256267  0.0038640   6.632
## z_wler:z_joy              -0.0277759  0.0082395  -3.371
## logits:z_meta              0.0317356  0.0037575   8.446
## z_wler:z_meta             -0.0341360  0.0084981  -4.017
## z_joy:z_meta               0.0100988  0.0085926   1.175
## logits:z_escs              0.0106516  0.0033096   3.218
## logits:gender             -0.0009279  0.0067032  -0.138
## logits:z_wler:z_joy       -0.0037725  0.0035023  -1.077
## logits:z_wler:z_meta       0.0068128  0.0035509   1.919
## logits:z_joy:z_meta       -0.0000327  0.0036708  -0.009
## z_wler:z_joy:z_meta        0.0027625  0.0074770   0.369
## logits:z_wler:z_joy:z_meta -0.0143719  0.0031987  -4.493

##
## Correlation matrix not shown by default, as p = 20 > 12.
## Use print(x, correlation=TRUE)  or
##   vcov(x)     if you need it
```

BELGUIM

```r
summary(time.dep.r[[3]])
```

```
## Linear mixed model fit by REML ['lmerMod']
## Formula: z_tottime ~ logits * z_wler * z_joy * z_meta + logits * z_escs +
##     logits * gender + (1 | user) + (1 | task) + (1 | schoolID)
##    Data: totdata
##
## REML criterion at convergence: 104492.5
##
## Scaled residuals:
##     Min      1Q  Median      3Q     Max
## -7.5944 -0.4736  0.0332  0.5506  6.5863
##
## Random effects:
##  Groups   Name        Variance Std.Dev.
##  user     (Intercept) 0.07895  0.2810
##  schoolID (Intercept) 0.03795  0.1948
##  task     (Intercept) 0.21167  0.4601
##  Residual             0.41154  0.6415
## Number of obs: 51096, groups:  user, 2681; schoolID, 248; task, 29
##
## Fixed effects:
##                               Estimate Std. Error t value
## (Intercept)                  0.0564304  0.0869718   0.649
## logits                       0.4492741  0.0819010   5.486
## z_wler                       0.0800592  0.0093006   8.608
## z_joy                        0.0258789  0.0082923   3.121
## z_meta                       0.0065936  0.0085270   0.773
## z_escs                       0.0295950  0.0073618   4.020
## gender                      -0.0250020  0.0140688  -1.777
## logits:z_wler                0.0948430  0.0037615  25.214
## logits:z_joy                 0.0159530  0.0035153   4.538
## z_wler:z_joy                -0.0612278  0.0084200  -7.272
## logits:z_meta                0.0248136  0.0035550   6.980
## z_wler:z_meta               -0.0213850  0.0076370  -2.800
## z_joy:z_meta                 0.0017960  0.0084802   0.212
## logits:z_escs                0.0210328  0.0030023   7.006
## logits:gender               -0.0101736  0.0057979  -1.755
## logits:z_wler:z_joy         -0.0063324  0.0035775  -1.770
## logits:z_wler:z_meta        -0.0109119  0.0031874  -3.423
## logits:z_joy:z_meta          0.0004217  0.0036355   0.116
## z_wler:z_joy:z_meta          0.0056863  0.0074865   0.760
## logits:z_wler:z_joy:z_meta -0.0040159  0.0032065  -1.252

##
## Correlation matrix not shown by default, as p = 20 > 12.
## Use print(x, correlation=TRUE)  or
##   vcov(x)     if you need it
```

CHILE

```r
summary(time.dep.r[[4]])
```

```
## Linear mixed model fit by REML ['lmerMod']
## Formula: z_tottime ~ logits * z_wler * z_joy * z_meta + logits * z_escs +
##     logits * gender + (1 | user) + (1 | task) + (1 | schoolID)
##    Data: totdata
##
## REML criterion at convergence: 67692.7
##
## Scaled residuals:
##     Min      1Q  Median      3Q     Max
## -6.2321 -0.4675  0.0601  0.5796  4.6570
##
## Random effects:
##  Groups   Name        Variance Std.Dev.
##  user     (Intercept) 0.11925  0.3453
##  schoolID (Intercept) 0.07199  0.2683
##  task     (Intercept) 0.14353  0.3788
##  Residual             0.54503  0.7383
## Number of obs: 28924, groups:  user, 1638; schoolID, 198; task, 29
##
## Fixed effects:
##                             Estimate Std. Error t value
## (Intercept)                 0.055837   0.074834   0.746
## logits                      0.332437   0.067657   4.914
## z_wler                      0.075564   0.013134   5.753
## z_joy                       0.034129   0.012059   2.830
## z_meta                      0.038321   0.011859   3.231
## z_escs                     -0.036866   0.013557  -2.719
## gender                     -0.021977   0.022694  -0.968
## logits:z_wler               0.070230   0.005193  13.524
## logits:z_joy                0.009335   0.005045   1.850
## z_wler:z_joy               -0.015131   0.011230  -1.347
## logits:z_meta               0.032812   0.004898   6.699
## z_wler:z_meta              -0.039605   0.011128  -3.559
## z_joy:z_meta               -0.011180   0.011784  -0.949
## logits:z_escs               0.016536   0.004782   3.458
## logits:gender               0.005497   0.008977   0.612
## logits:z_wler:z_joy        -0.005066   0.004647  -1.090
## logits:z_wler:z_meta        0.007100   0.004513   1.573
## logits:z_joy:z_meta         0.014191   0.004892   2.901
## z_wler:z_joy:z_meta        -0.006263   0.011008  -0.569
## logits:z_wler:z_joy:z_meta -0.008803   0.004678  -1.882

##
## Correlation matrix not shown by default, as p = 20 > 12.
## Use print(x, correlation=TRUE)  or
##   vcov(x)     if you need it
```

COLOMBIA

```
summary(time.dep.r[[5]])
```

```
## Linear mixed model fit by REML ['lmerMod']
## Formula: z_tottime ~ logits * z_wler * z_joy * z_meta + logits * z_escs +
##     logits * gender + (1 | user) + (1 | task) + (1 | schoolID)
##    Data: totdata
##
## REML criterion at convergence: 56176.8
##
## Scaled residuals:
##     Min      1Q  Median      3Q     Max
## -5.2177 -0.5229  0.0703  0.6195  4.8749
##
## Random effects:
##  Groups   Name        Variance Std.Dev.
##  user     (Intercept) 0.12062  0.3473
##  schoolID (Intercept) 0.06134  0.2477
##  task     (Intercept) 0.12305  0.3508
##  Residual             0.60404  0.7772
## Number of obs: 23017, groups:  user, 1345; schoolID, 136; task, 29
##
## Fixed effects:
##                               Estimate Std. Error t value
## (Intercept)                  8.130e-02  7.064e-02   1.151
## logits                       2.772e-01  6.282e-02   4.412
## z_wler                       6.286e-02  1.502e-02   4.184
## z_joy                        3.103e-02  1.375e-02   2.256
## z_meta                       3.235e-02  1.301e-02   2.486
## z_escs                      -4.179e-02  1.416e-02  -2.952
## gender                      -8.290e-02  2.336e-02  -3.549
## logits:z_wler                5.252e-02  6.255e-03   8.398
## logits:z_joy                -3.244e-05  5.963e-03  -0.005
## z_wler:z_joy                -4.001e-04  1.335e-02  -0.030
## logits:z_meta                1.342e-02  5.808e-03   2.310
## z_wler:z_meta                2.479e-03  1.222e-02   0.203
## z_joy:z_meta                -6.868e-03  1.272e-02  -0.540
## logits:z_escs                2.350e-02  5.538e-03   4.243
## logits:gender               -1.797e-02  1.032e-02  -1.741
## logits:z_wler:z_joy         -3.621e-03  5.813e-03  -0.623
## logits:z_wler:z_meta         1.702e-02  5.442e-03   3.128
## logits:z_joy:z_meta          3.033e-04  5.643e-03   0.054
## z_wler:z_joy:z_meta         -1.579e-02  1.166e-02  -1.355
## logits:z_wler:z_joy:z_meta -4.345e-04  5.240e-03  -0.083

##
## Correlation matrix not shown by default, as p = 20 > 12.
## Use print(x, correlation=TRUE)  or
##   vcov(x)     if you need it
```

DENMARK

```r
summary(time.dep.r[[6]])
```

```
## Linear mixed model fit by REML ['lmerMod']
## Formula: z_tottime ~ logits * z_wler * z_joy * z_meta + logits * z_escs +
##     logits * gender + (1 | user) + (1 | task) + (1 | schoolID)
##    Data: totdata
##
## REML criterion at convergence: 50300.6
##
## Scaled residuals:
##     Min      1Q  Median      3Q     Max
## -6.6088 -0.4660  0.0465  0.5547  6.4438
##
## Random effects:
##  Groups   Name        Variance Std.Dev.
##  user     (Intercept) 0.1111   0.3333
##  schoolID (Intercept) 0.0732   0.2705
##  task     (Intercept) 0.1892   0.4350
##  Residual             0.4643   0.6814
## Number of obs: 23011, groups:  user, 1204; schoolID, 220; task, 29
##
## Fixed effects:
##                              Estimate Std. Error t value
## (Intercept)                 0.0487991  0.0845997   0.577
## logits                      0.3463516  0.0776223   4.462
## z_wler                      0.1046258  0.0148023   7.068
## z_joy                       0.0355568  0.0139356   2.552
## z_meta                      0.0396245  0.0140098   2.828
## z_escs                     -0.0226094  0.0129553  -1.745
## gender                     -0.0456740  0.0238297  -1.917
## logits:z_wler               0.0970868  0.0054685  17.754
## logits:z_joy                0.0287113  0.0051897   5.532
## z_wler:z_joy               -0.0495660  0.0134286  -3.691
## logits:z_meta               0.0170310  0.0052095   3.269
## z_wler:z_meta              -0.0096722  0.0129181  -0.749
## z_joy:z_meta               -0.0018404  0.0135298  -0.136
## logits:z_escs               0.0110382  0.0047677   2.315
## logits:gender              -0.0107908  0.0093239  -1.157
## logits:z_wler:z_joy        -0.0042996  0.0050735  -0.847
## logits:z_wler:z_meta        0.0111533  0.0049033   2.275
## logits:z_joy:z_meta         0.0017718  0.0051134   0.346
## z_wler:z_joy:z_meta         0.0004221  0.0117531   0.036
## logits:z_wler:z_joy:z_meta -0.0114926  0.0044312  -2.594

##
## Correlation matrix not shown by default, as p = 20 > 12.
## Use print(x, correlation=TRUE)  or
##   vcov(x)     if you need it
```

SPAIN

```r
summary(time.dep.r[[7]])
```

```
## Linear mixed model fit by REML ['lmerMod']
## Formula: z_tottime ~ logits * z_wler * z_joy * z_meta + logits * z_escs +
##     logits * gender + (1 | user) + (1 | task) + (1 | schoolID)
##    Data: totdata
##
## REML criterion at convergence: 68861.5
##
## Scaled residuals:
##     Min      1Q  Median      3Q     Max
## -6.4175 -0.4752  0.0476  0.5577  5.2214
##
## Random effects:
##  Groups   Name        Variance Std.Dev.
##  user     (Intercept) 0.09178  0.3029
##  schoolID (Intercept) 0.07871  0.2806
##  task     (Intercept) 0.17462  0.4179
##  Residual             0.47782  0.6912
## Number of obs: 31294, groups:  user, 1649; schoolID, 164; task, 29
##
## Fixed effects:
##                                Estimate Std. Error t value
## (Intercept)                   0.0387489  0.0818019   0.474
## logits                        0.3690479  0.0745134   4.953
## z_wler                        0.0906213  0.0113144   8.009
## z_joy                         0.0170440  0.0107282   1.589
## z_meta                        0.0508662  0.0108648   4.682
## z_escs                        0.0120850  0.0105844   1.142
## gender                       -0.0166491  0.0187446  -0.888
## logits:z_wler                 0.0882782  0.0046978  18.792
## logits:z_joy                  0.0192469  0.0045446   4.235
## z_wler:z_joy                 -0.0287576  0.0102929  -2.794
## logits:z_meta                 0.0140745  0.0045495   3.094
## z_wler:z_meta                -0.0321625  0.0099080  -3.246
## z_joy:z_meta                  0.0029021  0.0100616   0.288
## logits:z_escs                 0.0154339  0.0040516   3.809
## logits:gender                -0.0148782  0.0079994  -1.860
## logits:z_wler:z_joy          -0.0099187  0.0043663  -2.272
## logits:z_wler:z_meta          0.0004029  0.0042423   0.095
## logits:z_joy:z_meta          -0.0007936  0.0042472  -0.187
## z_wler:z_joy:z_meta          -0.0128782  0.0100828  -1.277
## logits:z_wler:z_joy:z_meta  0.0030086  0.0042618   0.706

##
## Correlation matrix not shown by default, as p = 20 > 12.
## Use print(x, correlation=TRUE)  or
##   vcov(x)     if you need it
```

FRANCE

```r
summary(time.dep.r[[8]])
```

```
## Linear mixed model fit by REML ['lmerMod']
## Formula: z_tottime ~ logits * z_wler * z_joy * z_meta + logits * z_escs +
##     logits * gender + (1 | user) + (1 | task) + (1 | schoolID)
##    Data: totdata
##
## REML criterion at convergence: 48389.2
##
## Scaled residuals:
##     Min      1Q  Median      3Q     Max
## -7.7149 -0.4592  0.0422  0.5557  5.5028
##
## Random effects:
##  Groups   Name        Variance Std.Dev.
##  user     (Intercept) 0.06658  0.2580
##  schoolID (Intercept) 0.05974  0.2444
##  task     (Intercept) 0.21718  0.4660
##  Residual             0.42925  0.6552
## Number of obs: 23164, groups:  user, 1229; schoolID, 139; task, 29
##
## Fixed effects:
##                             Estimate Std. Error t value
## (Intercept)                0.0375404  0.0900397   0.417
## logits                     0.4295563  0.0830889   5.170
## z_wler                     0.0503412  0.0130358   3.862
## z_joy                      0.0180231  0.0108290   1.664
## z_meta                     0.0122412  0.0109665   1.116
## z_escs                     0.0016088  0.0105863   0.152
## gender                    -0.0146841  0.0187129  -0.785
## logits:z_wler              0.0929740  0.0054226  17.146
## logits:z_joy               0.0109134  0.0049511   2.204
## z_wler:z_joy              -0.0521404  0.0107836  -4.835
## logits:z_meta              0.0318781  0.0050020   6.373
## z_wler:z_meta             -0.0257437  0.0104967  -2.453
## z_joy:z_meta              -0.0002877  0.0110830  -0.026
## logits:z_escs              0.0183177  0.0045531   4.023
## logits:gender             -0.0169586  0.0086953  -1.950
## logits:z_wler:z_joy       -0.0001967  0.0048781  -0.040
## logits:z_wler:z_meta      -0.0139672  0.0048096  -2.904
## logits:z_joy:z_meta        0.0091757  0.0051002   1.799
## z_wler:z_joy:z_meta       -0.0105169  0.0094164  -1.117
## logits:z_wler:z_joy:z_meta -0.0097894  0.0044054  -2.222
##
## Correlation matrix not shown by default, as p = 20 > 12.
## Use print(x, correlation=TRUE)  or
##   vcov(x)     if you need it
```

HONG KONG-CHINA

```
summary(time.dep.r[[9]])
```

```
## Linear mixed model fit by REML ['lmerMod']
## Formula: z_tottime ~ logits * z_wler * z_joy * z_meta + logits * z_escs +
##     logits * gender + (1 | user) + (1 | task) + (1 | schoolID)
##    Data: totdata
##
## REML criterion at convergence: 52767.6
##
## Scaled residuals:
##     Min      1Q  Median      3Q     Max
## -7.2694 -0.4849  0.0538  0.5828  5.2927
##
## Random effects:
##  Groups   Name        Variance Std.Dev.
##  user     (Intercept) 0.09861  0.3140
##  schoolID (Intercept) 0.02382  0.1543
##  task     (Intercept) 0.22814  0.4776
##  Residual             0.38767  0.6226
## Number of obs: 26363, groups:  user, 1414; schoolID, 149; task, 29
##
## Fixed effects:
##                              Estimate Std. Error t value
## (Intercept)                 0.0416655  0.0908558   0.459
## logits                      0.4718833  0.0851121   5.544
## z_wler                      0.1122260  0.0120909   9.282
## z_joy                       0.0076189  0.0112028   0.680
## z_meta                      0.0140220  0.0108926   1.287
## z_escs                     -0.0194088  0.0109475  -1.773
## gender                      0.0046333  0.0206980   0.224
## logits:z_wler               0.0942007  0.0043543  21.634
## logits:z_joy                0.0023798  0.0043880   0.542
## z_wler:z_joy               -0.0349233  0.0105174  -3.321
## logits:z_meta               0.0202247  0.0041805   4.838
## z_wler:z_meta              -0.0247742  0.0111148  -2.229
## z_joy:z_meta                0.0029540  0.0105317   0.280
## logits:z_escs              -0.0027303  0.0038948  -0.701
## logits:gender              -0.0040711  0.0078532  -0.518
## logits:z_wler:z_joy        -0.0062213  0.0041206  -1.510
## logits:z_wler:z_meta       -0.0130100  0.0043152  -3.015
## logits:z_joy:z_meta         0.0130500  0.0041135   3.173
## z_wler:z_joy:z_meta        -0.0002741  0.0094058  -0.029
## logits:z_wler:z_joy:z_meta -0.0072613  0.0036390  -1.995

##
## Correlation matrix not shown by default, as p = 20 > 12.
## Use print(x, correlation=TRUE)  or
##   vcov(x)     if you need it
```

HUNGARY

```r
summary(time.dep.r[[10]])
```

```
## Linear mixed model fit by REML ['lmerMod']
## Formula: z_tottime ~ logits * z_wler * z_joy * z_meta + logits * z_escs +
##     logits * gender + (1 | user) + (1 | task) + (1 | schoolID)
##    Data: totdata
##
## REML criterion at convergence: 71116.3
##
## Scaled residuals:
##     Min      1Q  Median      3Q     Max
## -6.4194 -0.4828  0.0527  0.5751  5.5692
##
## Random effects:
##  Groups   Name        Variance Std.Dev.
##  user     (Intercept) 0.1071   0.3272
##  schoolID (Intercept) 0.1137   0.3372
##  task     (Intercept) 0.1704   0.4128
##  Residual             0.4750   0.6892
## Number of obs: 32296, groups:  user, 1697; schoolID, 183; task, 29
##
## Fixed effects:
##                             Estimate Std. Error t value
## (Intercept)                 0.042210   0.082240   0.513
## logits                      0.301071   0.073619   4.090
## z_wler                      0.122504   0.014281   8.578
## z_joy                       0.031966   0.011842   2.699
## z_meta                      0.027414   0.011613   2.361
## z_escs                     -0.007162   0.012072  -0.593
## gender                     -0.039025   0.021174  -1.843
## logits:z_wler               0.102326   0.005061  20.219
## logits:z_joy                0.020685   0.004668   4.431
## z_wler:z_joy               -0.037535   0.011401  -3.292
## logits:z_meta               0.012700   0.004557   2.787
## z_wler:z_meta              -0.046405   0.011002  -4.218
## z_joy:z_meta                0.003480   0.011029   0.316
## logits:z_escs               0.013208   0.004294   3.076
## logits:gender               0.012987   0.008090   1.605
## logits:z_wler:z_joy        -0.007873   0.004443  -1.772
## logits:z_wler:z_meta        0.005156   0.004255   1.212
## logits:z_joy:z_meta        -0.002271   0.004403  -0.516
## z_wler:z_joy:z_meta        -0.003486   0.010082  -0.346
## logits:z_wler:z_joy:z_meta  0.002445   0.003974   0.615
##
## Correlation matrix not shown by default, as p = 20 > 12.
## Use print(x, correlation=TRUE)  or
##   vcov(x)     if you need it
```

IRELAND

```r
summary(time.dep.r[[11]])
```

```
## Linear mixed model fit by REML ['lmerMod']
## Formula: z_tottime ~ logits * z_wler * z_joy * z_meta + logits * z_escs +
##     logits * gender + (1 | user) + (1 | task) + (1 | schoolID)
##    Data: totdata
##
## REML criterion at convergence: 53763.3
##
## Scaled residuals:
##     Min      1Q  Median      3Q     Max
## -7.4984 -0.4775  0.0374  0.5583  6.8914
##
## Random effects:
##  Groups   Name        Variance Std.Dev.
##  user     (Intercept) 0.08834  0.2972
##  schoolID (Intercept) 0.02073  0.1440
##  task     (Intercept) 0.22374  0.4730
##  Residual             0.44047  0.6637
## Number of obs: 25383, groups:  user, 1336; schoolID, 138; task, 29
##
## Fixed effects:
##                              Estimate Std. Error t value
## (Intercept)                  0.077918   0.090029   0.865
## logits                       0.463263   0.084308   5.495
## z_wler                       0.110008   0.012333   8.920
## z_joy                       -0.022812   0.011505  -1.983
## z_meta                       0.003644   0.011398   0.320
## z_escs                      -0.008602   0.010559  -0.815
## gender                      -0.073853   0.022736  -3.248
## logits:z_wler                0.099764   0.005137  19.422
## logits:z_joy                 0.018134   0.004838   3.749
## z_wler:z_joy                -0.043071   0.011113  -3.876
## logits:z_meta                0.019914   0.004837   4.117
## z_wler:z_meta               -0.052364   0.010626  -4.928
## z_joy:z_meta                 0.022843   0.011042   2.069
## logits:z_escs                0.013557   0.004330   3.131
## logits:gender               -0.056250   0.008393  -6.702
## logits:z_wler:z_joy         -0.017111   0.004694  -3.645
## logits:z_wler:z_meta         0.003232   0.004460   0.725
## logits:z_joy:z_meta          0.009246   0.004702   1.966
## z_wler:z_joy:z_meta          0.002156   0.010097   0.214
## logits:z_wler:z_joy:z_meta -0.013165   0.004296  -3.065

##
## Correlation matrix not shown by default, as p = 20 > 12.
## Use print(x, correlation=TRUE)  or
##   vcov(x)     if you need it
```

ICELAND

```r
summary(time.dep.r[[12]])
```

```
## Linear mixed model fit by REML ['lmerMod']
## Formula: z_tottime ~ logits * z_wler * z_joy * z_meta + logits * z_escs +
##     logits * gender + (1 | user) + (1 | task) + (1 | schoolID)
##    Data: totdata
##
## REML criterion at convergence: 36811.2
##
## Scaled residuals:
##     Min      1Q  Median      3Q     Max
## -6.4375 -0.5042  0.0363  0.5633  5.0433
##
## Random effects:
##  Groups   Name        Variance Std.Dev.
##  user     (Intercept) 0.11638  0.3412
##  schoolID (Intercept) 0.05191  0.2278
##  task     (Intercept) 0.20674  0.4547
##  Residual             0.41449  0.6438
## Number of obs: 17706, groups:  user, 930; schoolID, 118; task, 29
##
## Fixed effects:
##                             Estimate Std. Error t value
## (Intercept)                 0.089198   0.089143   1.001
## logits                      0.381135   0.081131   4.698
## z_wler                      0.090488   0.016535   5.473
## z_joy                       0.016163   0.015956   1.013
## z_meta                      0.076438   0.015071   5.072
## z_escs                      0.002471   0.014564   0.170
## gender                     -0.082012   0.028177  -2.911
## logits:z_wler               0.097344   0.005915  16.456
## logits:z_joy                0.034065   0.005870   5.803
## z_wler:z_joy               -0.015930   0.015085  -1.056
## logits:z_meta               0.026835   0.005462   4.913
## z_wler:z_meta              -0.054207   0.015794  -3.432
## z_joy:z_meta                0.011705   0.015324   0.764
## logits:z_escs               0.013230   0.004898   2.701
## logits:gender              -0.037061   0.010303  -3.597
## logits:z_wler:z_joy        -0.007554   0.005531  -1.366
## logits:z_wler:z_meta        0.001565   0.005847   0.268
## logits:z_joy:z_meta        -0.017946   0.005657  -3.172
## z_wler:z_joy:z_meta        -0.016716   0.013968  -1.197
## logits:z_wler:z_joy:z_meta -0.016361   0.005143  -3.181

##
## Correlation matrix not shown by default, as p = 20 > 12.
## Use print(x, correlation=TRUE)  or
##   vcov(x)     if you need it
```

JAPAN

```r
summary(time.dep.r[[13]])
```

```
## Linear mixed model fit by REML ['lmerMod']
## Formula: z_tottime ~ logits * z_wler * z_joy * z_meta + logits * z_escs +
##     logits * gender + (1 | user) + (1 | task) + (1 | schoolID)
##    Data: totdata
##
## REML criterion at convergence: 41206.5
##
## Scaled residuals:
##     Min      1Q  Median      3Q     Max
## -6.7977 -0.4868  0.0359  0.5608  6.1033
##
## Random effects:
##  Groups   Name        Variance Std.Dev.
##  user     (Intercept) 0.063923 0.25283
##  schoolID (Intercept) 0.007502 0.08662
##  task     (Intercept) 0.273491 0.52296
##  Residual             0.358958 0.59913
## Number of obs: 21591, groups:  user, 1155; schoolID, 41; task, 29
##
## Fixed effects:
##                               Estimate Std. Error t value
## (Intercept)                  0.0661054  0.0989562   0.668
## logits                       0.5211900  0.0931561   5.595
## z_wler                       0.0635450  0.0111782   5.685
## z_joy                       -0.0075631  0.0099476  -0.760
## z_meta                       0.0259091  0.0103127   2.512
## z_escs                      -0.0030977  0.0093770  -0.330
## gender                      -0.0277232  0.0185716  -1.493
## logits:z_wler                0.0628117  0.0048756  12.883
## logits:z_joy                 0.0133554  0.0045534   2.933
## z_wler:z_joy                -0.0333912  0.0104321  -3.201
## logits:z_meta                0.0177818  0.0047044   3.780
## z_wler:z_meta               -0.0395571  0.0088274  -4.481
## z_joy:z_meta                -0.0022161  0.0103158  -0.215
## logits:z_escs                0.0007855  0.0041264   0.190
## logits:gender               -0.0027755  0.0081129  -0.342
## logits:z_wler:z_joy         -0.0169244  0.0048011  -3.525
## logits:z_wler:z_meta         0.0026116  0.0039150   0.667
## logits:z_joy:z_meta         -0.0112353  0.0047455  -2.368
## z_wler:z_joy:z_meta          0.0183072  0.0084480   2.167
## logits:z_wler:z_joy:z_meta  0.0094963  0.0039628   2.396

##
## Correlation matrix not shown by default, as p = 20 > 12.
## Use print(x, correlation=TRUE)  or
##   vcov(x)     if you need it
```

KOREA

```r
summary(time.dep.r[[14]])
```

```
## Linear mixed model fit by REML ['lmerMod']
## Formula: z_tottime ~ logits * z_wler * z_joy * z_meta + logits * z_escs +
##     logits * gender + (1 | user) + (1 | task) + (1 | schoolID)
##    Data: totdata
##
## REML criterion at convergence: 51377.2
##
## Scaled residuals:
##     Min      1Q  Median      3Q     Max
## -8.6581 -0.5532  0.0042  0.5809  5.1798
##
## Random effects:
##  Groups   Name        Variance Std.Dev.
##  user     (Intercept) 0.06179  0.2486
##  schoolID (Intercept) 0.01419  0.1191
##  task     (Intercept) 0.28330  0.5323
##  Residual             0.33891  0.5822
## Number of obs: 27747, groups:  user, 1452; schoolID, 156; task, 29
##
## Fixed effects:
##                             Estimate Std. Error t value
## (Intercept)                 0.008793   0.100096   0.088
## logits                      0.559587   0.094759   5.905
## z_wler                      0.059320   0.009872   6.009
## z_joy                      -0.013554   0.008980  -1.509
## z_meta                     -0.002625   0.009244  -0.284
## z_escs                     -0.001103   0.008635  -0.128
## gender                      0.040534   0.018430   2.199
## logits:z_wler               0.050554   0.004209  12.012
## logits:z_joy                0.009703   0.003951   2.456
## z_wler:z_joy               -0.026874   0.009079  -2.960
## logits:z_meta               0.014094   0.004040   3.489
## z_wler:z_meta              -0.029717   0.008405  -3.536
## z_joy:z_meta                0.011061   0.009651   1.146
## logits:z_escs               0.008290   0.003595   2.306
## logits:gender              -0.026360   0.006993  -3.769
## logits:z_wler:z_joy        -0.004874   0.004023  -1.212
## logits:z_wler:z_meta       -0.010308   0.003694  -2.790
## logits:z_joy:z_meta        -0.003099   0.004235  -0.732
## z_wler:z_joy:z_meta         0.011607   0.007557   1.536
## logits:z_wler:z_joy:z_meta  0.001534   0.003331   0.460

##
## Correlation matrix not shown by default, as p = 20 > 12.
## Use print(x, correlation=TRUE)  or
##   vcov(x)     if you need it
```

MACAO-CHINA

```r
summary(time.dep.r[[15]])
```

```
## Linear mixed model fit by REML ['lmerMod']
## Formula: z_tottime ~ logits * z_wler * z_joy * z_meta + logits * z_escs +
##     logits * gender + (1 | user) + (1 | task) + (1 | schoolID)
##    Data: totdata
##
## REML criterion at convergence: 90494.6
##
## Scaled residuals:
##     Min      1Q  Median      3Q     Max
## -7.2790 -0.5139  0.0510  0.5951  6.1843
##
## Random effects:
##  Groups   Name        Variance Std.Dev.
##  user     (Intercept) 0.07484  0.2736
##  schoolID (Intercept) 0.01179  0.1086
##  task     (Intercept) 0.25153  0.5015
##  Residual             0.39651  0.6297
## Number of obs: 45159, groups:  user, 2484; schoolID, 44; task, 29
##
## Fixed effects:
##                             Estimate Std. Error t value
## (Intercept)                 0.053592   0.095196   0.563
## logits                      0.531319   0.089259   5.953
## z_wler                      0.050365   0.007904   6.372
## z_joy                       0.003785   0.007324   0.517
## z_meta                      0.016412   0.007113   2.307
## z_escs                     -0.010355   0.007341  -1.411
## gender                     -0.054267   0.015009  -3.615
## logits:z_wler               0.094521   0.003291  28.720
## logits:z_joy                0.014969   0.003330   4.495
## z_wler:z_joy               -0.038714   0.006695  -5.783
## logits:z_meta               0.009376   0.003182   2.947
## z_wler:z_meta              -0.020788   0.006735  -3.087
## z_joy:z_meta                0.012569   0.006794   1.850
## logits:z_escs              -0.004382   0.002967  -1.477
## logits:gender              -0.037170   0.006171  -6.024
## logits:z_wler:z_joy        -0.006514   0.003034  -2.147
## logits:z_wler:z_meta       -0.004462   0.003051  -1.463
## logits:z_joy:z_meta         0.005695   0.003072   1.854
## z_wler:z_joy:z_meta        -0.003203   0.005401  -0.593
## logits:z_wler:z_joy:z_meta -0.001052   0.002430  -0.433

##
## Correlation matrix not shown by default, as p = 20 > 12.
## Use print(x, correlation=TRUE)  or
##   vcov(x)     if you need it
```

NORWAY

```r
summary(time.dep.r[[16]])
```

```
## Linear mixed model fit by REML ['lmerMod']
## Formula: z_tottime ~ logits * z_wler * z_joy * z_meta + logits * z_escs +
##     logits * gender + (1 | user) + (1 | task) + (1 | schoolID)
##    Data: totdata
##
## REML criterion at convergence: 76507.6
##
## Scaled residuals:
##     Min      1Q  Median      3Q     Max
## -7.0443 -0.4924  0.0419  0.5756  5.9013
##
## Random effects:
##  Groups   Name        Variance Std.Dev.
##  user     (Intercept) 0.12279  0.3504
##  schoolID (Intercept) 0.05744  0.2397
##  task     (Intercept) 0.20478  0.4525
##  Residual             0.43160  0.6570
## Number of obs: 36225, groups:  user, 1902; schoolID, 180; task, 29
##
## Fixed effects:
##                               Estimate Std. Error t value
## (Intercept)                   0.074360   0.087069   0.854
## logits                        0.358228   0.080614   4.444
## z_wler                        0.131159   0.011667  11.242
## z_joy                         0.031972   0.011121   2.875
## z_meta                        0.019317   0.011067   1.745
## z_escs                       -0.019205   0.009831  -1.954
## gender                       -0.044086   0.019187  -2.298
## logits:z_wler                 0.101996   0.004239  24.059
## logits:z_joy                  0.022661   0.004035   5.616
## z_wler:z_joy                 -0.046112   0.010535  -4.377
## logits:z_meta                 0.016443   0.004035   4.075
## z_wler:z_meta                -0.043273   0.010104  -4.283
## z_joy:z_meta                  0.008091   0.010591   0.764
## logits:z_escs                 0.005648   0.003486   1.620
## logits:gender                -0.010880   0.007173  -1.517
## logits:z_wler:z_joy           0.003068   0.003867   0.793
## logits:z_wler:z_meta         -0.009012   0.003693  -2.440
## logits:z_joy:z_meta           0.004721   0.003905   1.209
## z_wler:z_joy:z_meta           0.013150   0.008402   1.565
## logits:z_wler:z_joy:z_meta -0.006485   0.003064  -2.117

##
## Correlation matrix not shown by default, as p = 20 > 12.
## Use print(x, correlation=TRUE)  or
##   vcov(x)     if you need it
```

NEW ZEALAND

```
summary(time.dep.r[[17]])
```

```
## Linear mixed model fit by REML ['lmerMod']
## Formula: z_tottime ~ logits * z_wler * z_joy * z_meta + logits * z_escs +
##     logits * gender + (1 | user) + (1 | task) + (1 | schoolID)
##    Data: totdata
##
## REML criterion at convergence: 64321.2
##
## Scaled residuals:
##     Min      1Q  Median      3Q     Max
## -7.3803 -0.4763  0.0326  0.5484  6.6725
##
## Random effects:
##  Groups   Name        Variance Std.Dev.
##  user     (Intercept) 0.09108  0.3018
##  schoolID (Intercept) 0.02539  0.1593
##  task     (Intercept) 0.23433  0.4841
##  Residual             0.39321  0.6271
## Number of obs: 32018, groups:  user, 1687; schoolID, 145; task, 29
##
## Fixed effects:
##                              Estimate Std. Error t value
## (Intercept)                 0.0987008  0.0919232   1.074
## logits                      0.4787232  0.0862162   5.553
## z_wler                      0.0185470  0.0113929   1.628
## z_joy                       0.0295036  0.0107380   2.748
## z_meta                      0.0287287  0.0102225   2.810
## z_escs                     -0.0008652  0.0096376  -0.090
## gender                     -0.0801077  0.0204313  -3.921
## logits:z_wler               0.0907153  0.0046012  19.715
## logits:z_joy                0.0218164  0.0043461   5.020
## z_wler:z_joy               -0.0434919  0.0093626  -4.645
## logits:z_meta               0.0294064  0.0040989   7.174
## z_wler:z_meta              -0.0603485  0.0097186  -6.210
## z_joy:z_meta               -0.0114671  0.0097628  -1.175
## logits:z_escs               0.0143240  0.0037150   3.856
## logits:gender              -0.0273652  0.0073036  -3.747
## logits:z_wler:z_joy        -0.0050610  0.0037905  -1.335
## logits:z_wler:z_meta       -0.0139964  0.0039082  -3.581
## logits:z_joy:z_meta        -0.0057833  0.0039599  -1.460
## z_wler:z_joy:z_meta         0.0111536  0.0084679   1.317
## logits:z_wler:z_joy:z_meta -0.0076935  0.0034331  -2.241

##
## Correlation matrix not shown by default, as p = 20 > 12.
## Use print(x, correlation=TRUE)  or
##   vcov(x)     if you need it
```

SWEDEN

```r
summary(time.dep.r[[18]])
```

```
## Linear mixed model fit by REML ['lmerMod']
## Formula: z_tottime ~ logits * z_wler * z_joy * z_meta + logits * z_escs +
##     logits * gender + (1 | user) + (1 | task) + (1 | schoolID)
##    Data: totdata
##
## REML criterion at convergence: 70626
##
## Scaled residuals:
##     Min      1Q  Median      3Q     Max
## -7.6801 -0.4834  0.0324  0.5525  7.0580
##
## Random effects:
##  Groups   Name        Variance Std.Dev.
##  user     (Intercept) 0.12477  0.3532
##  schoolID (Intercept) 0.02241  0.1497
##  task     (Intercept) 0.20812  0.4562
##  Residual             0.43342  0.6583
## Number of obs: 33405, groups:  user, 1760; schoolID, 179; task, 29
##
## Fixed effects:
##                             Estimate Std. Error t value
## (Intercept)                0.0748750  0.0867085   0.864
## logits                     0.3867098  0.0812784   4.758
## z_wler                     0.1015969  0.0128173   7.927
## z_joy                      0.0375066  0.0116814   3.211
## z_meta                     0.0391137  0.0115996   3.372
## z_escs                     0.0185164  0.0105270   1.759
## gender                    -0.0429426  0.0205051  -2.094
## logits:z_wler              0.0939511  0.0046685  20.125
## logits:z_joy               0.0183929  0.0042877   4.290
## z_wler:z_joy              -0.0696576  0.0108298  -6.432
## logits:z_meta              0.0280632  0.0041674   6.734
## z_wler:z_meta             -0.0332060  0.0111321  -2.983
## z_joy:z_meta               0.0102281  0.0114208   0.896
## logits:z_escs              0.0129221  0.0038128   3.389
## logits:gender             -0.0117051  0.0076361  -1.533
## logits:z_wler:z_joy       -0.0060196  0.0039925  -1.508
## logits:z_wler:z_meta      -0.0101156  0.0040804  -2.479
## logits:z_joy:z_meta        0.0091135  0.0042011   2.169
## z_wler:z_joy:z_meta        0.0001551  0.0088717   0.017
## logits:z_wler:z_joy:z_meta -0.0082859  0.0033119  -2.502

##
## Correlation matrix not shown by default, as p = 20 > 12.
## Use print(x, correlation=TRUE)  or
##   vcov(x)     if you need it
```

POLAND

```
summary(time.dep.r[[19]])
```

```
## Linear mixed model fit by REML ['lmerMod']
## Formula: z_tottime ~ logits * z_wler * z_joy * z_meta + logits * z_escs +
##     logits * gender + (1 | user) + (1 | task) + (1 | schoolID)
##    Data: totdata
##
## REML criterion at convergence: 76249.1
##
## Scaled residuals:
##     Min      1Q  Median      3Q     Max
## -6.7555 -0.4756  0.0608  0.5823  6.2396
##
## Random effects:
##  Groups   Name        Variance Std.Dev.
##  user     (Intercept) 0.11564  0.3401
##  schoolID (Intercept) 0.05996  0.2449
##  task     (Intercept) 0.17142  0.4140
##  Residual             0.46845  0.6844
## Number of obs: 34848, groups:  user, 1823; schoolID, 179; task, 29
##
## Fixed effects:
##                              Estimate Std. Error t value
## (Intercept)                 4.898e-02  8.026e-02   0.610
## logits                      3.550e-01  7.381e-02   4.810
## z_wler                      1.374e-01  1.191e-02  11.537
## z_joy                       3.367e-02  1.135e-02   2.968
## z_meta                      2.327e-02  1.054e-02   2.207
## z_escs                     -1.119e-02  1.105e-02  -1.012
## gender                     -5.948e-03  1.978e-02  -0.301
## logits:z_wler               8.549e-02  4.490e-03  19.038
## logits:z_joy                3.435e-02  4.369e-03   7.861
## z_wler:z_joy               -5.110e-02  1.083e-02  -4.719
## logits:z_meta               1.760e-02  4.085e-03   4.309
## z_wler:z_meta              -4.701e-02  1.054e-02  -4.462
## z_joy:z_meta                1.056e-02  1.072e-02   0.985
## logits:z_escs               1.943e-02  3.903e-03   4.977
## logits:gender               1.522e-03  7.754e-03   0.196
## logits:z_wler:z_joy        -1.709e-02  4.192e-03  -4.076
## logits:z_wler:z_meta       -4.479e-03  4.050e-03  -1.106
## logits:z_joy:z_meta         5.493e-03  4.157e-03   1.321
## z_wler:z_joy:z_meta         4.005e-03  9.497e-03   0.422
## logits:z_wler:z_joy:z_meta -2.829e-05  3.635e-03  -0.008

##
## Correlation matrix not shown by default, as p = 20 > 12.
## Use print(x, correlation=TRUE)  or
##   vcov(x)     if you need it
```

Dependent variable average time on relevant pages

AUSTRALIA

```r
summary(reltime.dep.r[[1]])
```

```
## Linear mixed model fit by REML ['lmerMod']
## Formula: z_reltime ~ logits * z_wler * z_joy * z_meta + logits * z_escs +
##     logits * gender + (1 | user) + (1 | task) + (1 | schoolID)
##    Data: totdata
##
## REML criterion at convergence: 95046.3
##
## Scaled residuals:
##     Min      1Q  Median      3Q     Max
## -8.7500 -0.4508  0.0641  0.5654  7.2569
##
## Random effects:
##  Groups   Name        Variance Std.Dev.
##  user     (Intercept) 0.08869  0.2978
##  schoolID (Intercept) 0.02438  0.1561
##  task     (Intercept) 0.51436  0.7172
##  Residual             0.30846  0.5554
## Number of obs: 53607, groups:  user, 2800; schoolID, 334; task, 29
##
## Fixed effects:
##                             Estimate Std. Error t value
## (Intercept)                 0.056708   0.133826   0.424
## logits                      0.218424   0.127540   1.713
## z_wler                      0.091050   0.008442  10.785
## z_joy                       0.014770   0.008122   1.819
## z_meta                      0.023330   0.007820   2.984
## z_escs                     -0.009152   0.007311  -1.252
## gender                     -0.053156   0.014036  -3.787
## logits:z_wler               0.085453   0.003003  28.460
## logits:z_joy                0.026830   0.002954   9.084
## z_wler:z_joy               -0.034095   0.007552  -4.515
## logits:z_meta               0.021124   0.002815   7.503
## z_wler:z_meta              -0.033088   0.007667  -4.316
## z_joy:z_meta                0.002472   0.007840   0.315
## logits:z_escs               0.005668   0.002532   2.238
## logits:gender              -0.030892   0.004875  -6.337
## logits:z_wler:z_joy        -0.006796   0.002739  -2.481
## logits:z_wler:z_meta       -0.002606   0.002785  -0.936
## logits:z_joy:z_meta        -0.002021   0.002864  -0.706
## z_wler:z_joy:z_meta         0.002032   0.006549   0.310
## logits:z_wler:z_joy:z_meta -0.009671   0.002361  -4.095
##
## Correlation matrix not shown by default, as p = 20 > 12.
## Use print(x, correlation=TRUE)  or
##   vcov(x)     if you need it
```

AUSTRIA

```r
summary(reltime.dep.r[[2]])
```

```
## Linear mixed model fit by REML ['lmerMod']
## Formula: z_reltime ~ logits * z_wler * z_joy * z_meta + logits * z_escs +
##     logits * gender + (1 | user) + (1 | task) + (1 | schoolID)
##    Data: totdata
##
## REML criterion at convergence: 89824
##
## Scaled residuals:
##     Min      1Q  Median      3Q     Max
## -6.7628 -0.4440  0.0770  0.5687  5.5640
##
## Random effects:
##  Groups   Name        Variance Std.Dev.
##  user     (Intercept) 0.06410  0.2532
##  schoolID (Intercept) 0.09438  0.3072
##  task     (Intercept) 0.45366  0.6735
##  Residual             0.35398  0.5950
## Number of obs: 47326, groups:  user, 2483; schoolID, 253; task, 29
##
## Fixed effects:
##                              Estimate Std. Error t value
## (Intercept)                 0.0212825  0.1270196   0.168
## logits                      0.0803956  0.1198035   0.671
## z_wler                      0.1060124  0.0088954  11.918
## z_joy                       0.0316082  0.0078006   4.052
## z_meta                      0.0380129  0.0076380   4.977
## z_escs                      0.0006761  0.0069511   0.097
## gender                      0.0005003  0.0145656   0.034
## logits:z_wler               0.0786935  0.0034242  22.982
## logits:z_joy                0.0230464  0.0033044   6.974
## z_wler:z_joy               -0.0229337  0.0069498  -3.300
## logits:z_meta               0.0259865  0.0032133   8.087
## z_wler:z_meta              -0.0262662  0.0071677  -3.665
## z_joy:z_meta                0.0110485  0.0072484   1.524
## logits:z_escs               0.0092514  0.0028303   3.269
## logits:gender              -0.0011397  0.0057324  -0.199
## logits:z_wler:z_joy        -0.0045075  0.0029951  -1.505
## logits:z_wler:z_meta        0.0058567  0.0030366   1.929
## logits:z_joy:z_meta         0.0014500  0.0031392   0.462
## z_wler:z_joy:z_meta         0.0008051  0.0063072   0.128
## logits:z_wler:z_joy:z_meta -0.0124790  0.0027354  -4.562
##
## Correlation matrix not shown by default, as p = 20 > 12.
## Use print(x, correlation=TRUE)  or
##   vcov(x)     if you need it
```

BELGUIM

```r
summary(reltime.dep.r[[3]])
```

```
## Linear mixed model fit by REML ['lmerMod']
## Formula: z_reltime ~ logits * z_wler * z_joy * z_meta + logits * z_escs +
##     logits * gender + (1 | user) + (1 | task) + (1 | schoolID)
##    Data: totdata
##
## REML criterion at convergence: 88644
##
## Scaled residuals:
##     Min      1Q  Median      3Q     Max
## -8.6610 -0.4485  0.0599  0.5602  6.4409
##
## Random effects:
##  Groups   Name        Variance Std.Dev.
##  user     (Intercept) 0.0565   0.2377
##  schoolID (Intercept) 0.0253   0.1591
##  task     (Intercept) 0.5514   0.7425
##  Residual             0.3019   0.5495
## Number of obs: 51096, groups:  user, 2681; schoolID, 248; task, 29
##
## Fixed effects:
##                            Estimate Std. Error t value
## (Intercept)                0.039166   0.138546   0.283
## logits                     0.203039   0.132048   1.538
## z_wler                     0.080730   0.007871  10.256
## z_joy                      0.022179   0.007027   3.156
## z_meta                     0.010780   0.007223   1.492
## z_escs                     0.024972   0.006233   4.006
## gender                    -0.026646   0.011916  -2.236
## logits:z_wler              0.084282   0.003222  26.160
## logits:z_joy               0.013442   0.003011   4.464
## z_wler:z_joy              -0.050428   0.007136  -7.067
## logits:z_meta              0.020799   0.003045   6.831
## z_wler:z_meta             -0.017654   0.006469  -2.729
## z_joy:z_meta               0.002736   0.007188   0.381
## logits:z_escs              0.017388   0.002572   6.762
## logits:gender             -0.009335   0.004966  -1.880
## logits:z_wler:z_joy       -0.006469   0.003064  -2.111
## logits:z_wler:z_meta      -0.008358   0.002730  -3.061
## logits:z_joy:z_meta        0.002173   0.003114   0.698
## z_wler:z_joy:z_meta        0.004333   0.006346   0.683
## logits:z_wler:z_joy:z_meta -0.003594   0.002746  -1.309

##
## Correlation matrix not shown by default, as p = 20 > 12.
## Use print(x, correlation=TRUE)  or
##   vcov(x)     if you need it
```

CHILE

```r
summary(reltime.dep.r[[4]])
```

```
## Linear mixed model fit by REML ['lmerMod']
## Formula: z_reltime ~ logits * z_wler * z_joy * z_meta + logits * z_escs +
##     logits * gender + (1 | user) + (1 | task) + (1 | schoolID)
##    Data: totdata
##
## REML criterion at convergence: 56127.6
##
## Scaled residuals:
##     Min      1Q  Median      3Q     Max
## -6.5077 -0.4538  0.0823  0.5856  4.8608
##
## Random effects:
##  Groups   Name        Variance Std.Dev.
##  user     (Intercept) 0.07679  0.2771
##  schoolID (Intercept) 0.04173  0.2043
##  task     (Intercept) 0.51572  0.7181
##  Residual             0.36560  0.6046
## Number of obs: 28924, groups:  user, 1638; schoolID, 198; task, 29
##
## Fixed effects:
##                              Estimate Std. Error t value
## (Intercept)                 2.280e-02  1.348e-01   0.169
## logits                      1.099e-01  1.278e-01   0.860
## z_wler                      6.779e-02  1.055e-02   6.427
## z_joy                       2.924e-02  9.703e-03   3.013
## z_meta                      3.875e-02  9.539e-03   4.062
## z_escs                     -2.998e-02  1.085e-02  -2.764
## gender                     -3.335e-02  1.824e-02  -1.829
## logits:z_wler               5.578e-02  4.253e-03  13.117
## logits:z_joy                1.041e-02  4.132e-03   2.520
## z_wler:z_joy               -1.801e-02  9.038e-03  -1.993
## logits:z_meta               2.803e-02  4.011e-03   6.989
## z_wler:z_meta              -2.960e-02  8.945e-03  -3.309
## z_joy:z_meta               -9.888e-03  9.481e-03  -1.043
## logits:z_escs               1.266e-02  3.916e-03   3.234
## logits:gender               4.537e-05  7.352e-03   0.006
## logits:z_wler:z_joy        -4.979e-03  3.805e-03  -1.308
## logits:z_wler:z_meta        5.428e-03  3.696e-03   1.468
## logits:z_joy:z_meta         1.095e-02  4.006e-03   2.733
## z_wler:z_joy:z_meta        -1.192e-03  8.860e-03  -0.135
## logits:z_wler:z_joy:z_meta -6.755e-03  3.831e-03  -1.763

##
## Correlation matrix not shown by default, as p = 20 > 12.
## Use print(x, correlation=TRUE)  or
##   vcov(x)     if you need it
```

COLOMBIA

```r
summary(reltime.dep.r[[5]])
```

```
## Linear mixed model fit by REML ['lmerMod']
## Formula: z_reltime ~ logits * z_wler * z_joy * z_meta + logits * z_escs +
##     logits * gender + (1 | user) + (1 | task) + (1 | schoolID)
##    Data: totdata
##
## REML criterion at convergence: 45879.7
##
## Scaled residuals:
##     Min      1Q  Median      3Q     Max
## -5.3922 -0.4986  0.0895  0.6236  5.3101
##
## Random effects:
##  Groups   Name        Variance Std.Dev.
##  user     (Intercept) 0.07300  0.2702
##  schoolID (Intercept) 0.03498  0.1870
##  task     (Intercept) 0.50299  0.7092
##  Residual             0.38624  0.6215
## Number of obs: 23017, groups:  user, 1345; schoolID, 136; task, 29
##
## Fixed effects:
##                              Estimate Std. Error t value
## (Intercept)                 0.0316362  0.1333578   0.237
## logits                      0.0772331  0.1262205   0.612
## z_wler                      0.0514936  0.0117392   4.386
## z_joy                       0.0235120  0.0107532   2.187
## z_meta                      0.0271893  0.0101818   2.670
## z_escs                     -0.0356047  0.0110459  -3.223
## gender                     -0.0818115  0.0182734  -4.477
## logits:z_wler               0.0413936  0.0050008   8.277
## logits:z_joy                0.0013840  0.0047678   0.290
## z_wler:z_joy               -0.0017556  0.0104411  -0.168
## logits:z_meta               0.0110599  0.0046436   2.382
## z_wler:z_meta               0.0037422  0.0095633   0.391
## z_joy:z_meta               -0.0003481  0.0099496  -0.035
## logits:z_escs               0.0157026  0.0044282   3.546
## logits:gender              -0.0196268  0.0082547  -2.378
## logits:z_wler:z_joy        -0.0046873  0.0046474  -1.009
## logits:z_wler:z_meta        0.0149201  0.0043507   3.429
## logits:z_joy:z_meta         0.0030215  0.0045121   0.670
## z_wler:z_joy:z_meta        -0.0153671  0.0091217  -1.685
## logits:z_wler:z_joy:z_meta  0.0017814  0.0041895   0.425

##
## Correlation matrix not shown by default, as p = 20 > 12.
## Use print(x, correlation=TRUE)  or
##   vcov(x)     if you need it
```

DENMARK

```r
summary(reltime.dep.r[[6]])
```

```
## Linear mixed model fit by REML ['lmerMod']
## Formula: z_reltime ~ logits * z_wler * z_joy * z_meta + logits * z_escs +
##     logits * gender + (1 | user) + (1 | task) + (1 | schoolID)
##    Data: totdata
##
## REML criterion at convergence: 42850.2
##
## Scaled residuals:
##     Min      1Q  Median      3Q     Max
## -8.2512 -0.4472  0.0700  0.5652  5.4975
##
## Random effects:
##  Groups   Name        Variance Std.Dev.
##  user     (Intercept) 0.07773  0.2788
##  schoolID (Intercept) 0.05143  0.2268
##  task     (Intercept) 0.51322  0.7164
##  Residual             0.33571  0.5794
## Number of obs: 23011, groups:  user, 1204; schoolID, 220; task, 29
##
## Fixed effects:
##                              Estimate Std. Error t value
## (Intercept)                 3.090e-02  1.347e-01   0.229
## logits                      1.391e-01  1.275e-01   1.091
## z_wler                      9.885e-02  1.242e-02   7.960
## z_joy                       3.245e-02  1.169e-02   2.776
## z_meta                      3.564e-02  1.175e-02   3.032
## z_escs                     -1.872e-02  1.087e-02  -1.722
## gender                     -3.833e-02  1.999e-02  -1.917
## logits:z_wler               7.995e-02  4.650e-03  17.193
## logits:z_joy                2.192e-02  4.413e-03   4.968
## z_wler:z_joy               -3.774e-02  1.127e-02  -3.350
## logits:z_meta               1.618e-02  4.430e-03   3.652
## z_wler:z_meta              -5.108e-03  1.084e-02  -0.471
## z_joy:z_meta               -4.278e-03  1.135e-02  -0.377
## logits:z_escs               9.214e-03  4.054e-03   2.273
## logits:gender              -5.774e-03  7.928e-03  -0.728
## logits:z_wler:z_joy        -3.644e-03  4.314e-03  -0.845
## logits:z_wler:z_meta        9.036e-03  4.169e-03   2.167
## logits:z_joy:z_meta         2.714e-03  4.348e-03   0.624
## z_wler:z_joy:z_meta         7.707e-05  9.860e-03   0.008
## logits:z_wler:z_joy:z_meta -7.081e-03  3.768e-03  -1.879
##
## Correlation matrix not shown by default, as p = 20 > 12.
## Use print(x, correlation=TRUE)  or
##   vcov(x)     if you need it
```

SPAIN

```r
summary(reltime.dep.r[[7]])
```

```
## Linear mixed model fit by REML ['lmerMod']
## Formula: z_reltime ~ logits * z_wler * z_joy * z_meta + logits * z_escs +
##     logits * gender + (1 | user) + (1 | task) + (1 | schoolID)
##    Data: totdata
##
## REML criterion at convergence: 57787.7
##
## Scaled residuals:
##     Min      1Q  Median      3Q     Max
## -7.5613 -0.4536  0.0706  0.5652  4.7857
##
## Random effects:
##  Groups   Name        Variance Std.Dev.
##  user     (Intercept) 0.06522  0.2554
##  schoolID (Intercept) 0.05148  0.2269
##  task     (Intercept) 0.52489  0.7245
##  Residual             0.33485  0.5787
## Number of obs: 31294, groups:  user, 1649; schoolID, 164; task, 29
##
## Fixed effects:
##                             Estimate Std. Error t value
## (Intercept)                1.677e-02  1.362e-01   0.123
## logits                     1.389e-01  1.289e-01   1.078
## z_wler                     8.633e-02  9.515e-03   9.073
## z_joy                      1.564e-02  9.025e-03   1.733
## z_meta                     4.803e-02  9.138e-03   5.256
## z_escs                     1.204e-02  8.891e-03   1.355
## gender                    -1.346e-02  1.577e-02  -0.854
## logits:z_wler              7.356e-02  3.933e-03  18.705
## logits:z_joy               1.492e-02  3.804e-03   3.923
## z_wler:z_joy              -2.158e-02  8.659e-03  -2.493
## logits:z_meta              1.205e-02  3.809e-03   3.165
## z_wler:z_meta             -2.404e-02  8.335e-03  -2.884
## z_joy:z_meta               4.287e-03  8.464e-03   0.506
## logits:z_escs              1.246e-02  3.392e-03   3.672
## logits:gender             -1.251e-02  6.697e-03  -1.867
## logits:z_wler:z_joy       -6.941e-03  3.655e-03  -1.899
## logits:z_wler:z_meta      -3.521e-04  3.551e-03  -0.099
## logits:z_joy:z_meta        7.703e-05  3.556e-03   0.022
## z_wler:z_joy:z_meta       -1.343e-02  8.481e-03  -1.584
## logits:z_wler:z_joy:z_meta  2.773e-03  3.568e-03   0.777

##
## Correlation matrix not shown by default, as p = 20 > 12.
## Use print(x, correlation=TRUE)  or
##   vcov(x)     if you need it
```

FRANCE

```r
summary(reltime.dep.r[[8]])
```

```
## Linear mixed model fit by REML ['lmerMod']
## Formula: z_reltime ~ logits * z_wler * z_joy * z_meta + logits * z_escs +
##     logits * gender + (1 | user) + (1 | task) + (1 | schoolID)
##    Data: totdata
##
## REML criterion at convergence: 40994.5
##
## Scaled residuals:
##     Min      1Q  Median      3Q     Max
## -7.7260 -0.4332  0.0672  0.5676  5.0408
##
## Random effects:
##  Groups   Name        Variance Std.Dev.
##  user     (Intercept) 0.04733  0.2176
##  schoolID (Intercept) 0.03783  0.1945
##  task     (Intercept) 0.54947  0.7413
##  Residual             0.31186  0.5584
## Number of obs: 23164, groups:  user, 1229; schoolID, 139; task, 29
##
## Fixed effects:
##                              Estimate Std. Error t value
## (Intercept)                 2.838e-02  1.391e-01   0.204
## logits                      1.940e-01  1.319e-01   1.471
## z_wler                      5.735e-02  1.098e-02   5.226
## z_joy                       1.402e-02  9.145e-03   1.533
## z_meta                      1.711e-02  9.259e-03   1.848
## z_escs                     -6.990e-04  8.924e-03  -0.078
## gender                     -2.818e-02  1.581e-02  -1.783
## logits:z_wler               7.992e-02  4.622e-03  17.293
## logits:z_joy                9.656e-03  4.220e-03   2.288
## z_wler:z_joy               -4.196e-02  9.106e-03  -4.609
## logits:z_meta               2.528e-02  4.263e-03   5.930
## z_wler:z_meta              -2.409e-02  8.863e-03  -2.718
## z_joy:z_meta                3.255e-03  9.360e-03   0.348
## logits:z_escs               1.471e-02  3.881e-03   3.791
## logits:gender              -1.498e-02  7.411e-03  -2.021
## logits:z_wler:z_joy        -5.926e-05  4.158e-03  -0.014
## logits:z_wler:z_meta       -1.377e-02  4.099e-03  -3.359
## logits:z_joy:z_meta         8.493e-03  4.347e-03   1.954
## z_wler:z_joy:z_meta        -8.931e-03  7.956e-03  -1.123
## logits:z_wler:z_joy:z_meta -7.259e-03  3.755e-03  -1.933

##
## Correlation matrix not shown by default, as p = 20 > 12.
## Use print(x, correlation=TRUE)  or
##   vcov(x)     if you need it
```

HONG KONG-CHINA

```r
summary(reltime.dep.r[[9]])
```

```
## Linear mixed model fit by REML ['lmerMod']
## Formula: z_reltime ~ logits * z_wler * z_joy * z_meta + logits * z_escs +
##     logits * gender + (1 | user) + (1 | task) + (1 | schoolID)
##    Data: totdata
##
## REML criterion at convergence: 47423.2
##
## Scaled residuals:
##     Min      1Q  Median      3Q     Max
## -8.6530 -0.4576  0.0749  0.5751  5.0880
##
## Random effects:
##  Groups   Name        Variance Std.Dev.
##  user     (Intercept) 0.07526  0.2743
##  schoolID (Intercept) 0.01934  0.1391
##  task     (Intercept) 0.48773  0.6984
##  Residual             0.31707  0.5631
## Number of obs: 26363, groups:  user, 1414; schoolID, 149; task, 29
##
## Fixed effects:
##                              Estimate Std. Error t value
## (Intercept)                 0.0257292  0.1308639   0.197
## logits                      0.2733781  0.1242599   2.200
## z_wler                      0.1128891  0.0106540  10.596
## z_joy                       0.0075330  0.0098541   0.764
## z_meta                      0.0118104  0.0095845   1.232
## z_escs                     -0.0148641  0.0096490  -1.540
## gender                     -0.0077939  0.0182167  -0.428
## logits:z_wler               0.0851231  0.0039376  21.618
## logits:z_joy               -0.0001528  0.0039679  -0.039
## z_wler:z_joy               -0.0307202  0.0092519  -3.320
## logits:z_meta               0.0203596  0.0037803   5.386
## z_wler:z_meta              -0.0204662  0.0097769  -2.093
## z_joy:z_meta                0.0039935  0.0092640   0.431
## logits:z_escs              -0.0008435  0.0035220  -0.240
## logits:gender              -0.0095701  0.0071014  -1.348
## logits:z_wler:z_joy        -0.0054448  0.0037262  -1.461
## logits:z_wler:z_meta       -0.0092447  0.0039022  -2.369
## logits:z_joy:z_meta         0.0097015  0.0037197   2.608
## z_wler:z_joy:z_meta        -0.0013527  0.0082742  -0.163
## logits:z_wler:z_joy:z_meta -0.0051889  0.0032907  -1.577
##
## Correlation matrix not shown by default, as p = 20 > 12.
## Use print(x, correlation=TRUE)  or
##   vcov(x)     if you need it
```

HUNGARY

```r
summary(reltime.dep.r[[10]])
```

```
## Linear mixed model fit by REML ['lmerMod']
## Formula: z_reltime ~ logits * z_wler * z_joy * z_meta + logits * z_escs +
##     logits * gender + (1 | user) + (1 | task) + (1 | schoolID)
##    Data: totdata
##
## REML criterion at convergence: 58978.6
##
## Scaled residuals:
##     Min      1Q  Median      3Q     Max
## -7.8815 -0.4635  0.0739  0.5790  5.1363
##
## Random effects:
##  Groups   Name        Variance Std.Dev.
##  user     (Intercept) 0.07283  0.2699
##  schoolID (Intercept) 0.06950  0.2636
##  task     (Intercept) 0.52179  0.7223
##  Residual             0.32599  0.5710
## Number of obs: 32296, groups:  user, 1697; schoolID, 183; task, 29
##
## Fixed effects:
##                              Estimate Std. Error t value
## (Intercept)                 0.0279211  0.1362062   0.205
## logits                      0.0852025  0.1284990   0.663
## z_wler                      0.1103056  0.0117395   9.396
## z_joy                       0.0245857  0.0097652   2.518
## z_meta                      0.0236275  0.0095750   2.468
## z_escs                     -0.0055431  0.0099282  -0.558
## gender                     -0.0417261  0.0174533  -2.391
## logits:z_wler               0.0844795  0.0041926  20.149
## logits:z_joy                0.0165859  0.0038672   4.289
## z_wler:z_joy               -0.0277722  0.0093990  -2.955
## logits:z_meta               0.0124419  0.0037750   3.296
## z_wler:z_meta              -0.0352753  0.0090655  -3.891
## z_joy:z_meta                0.0002081  0.0090962   0.023
## logits:z_escs               0.0116916  0.0035576   3.286
## logits:gender               0.0152210  0.0067017   2.271
## logits:z_wler:z_joy        -0.0058423  0.0036807  -1.587
## logits:z_wler:z_meta        0.0054401  0.0035248   1.543
## logits:z_joy:z_meta        -0.0021435  0.0036474  -0.588
## z_wler:z_joy:z_meta        -0.0069749  0.0083151  -0.839
## logits:z_wler:z_joy:z_meta  0.0013905  0.0032918   0.422

##
## Correlation matrix not shown by default, as p = 20 > 12.
## Use print(x, correlation=TRUE)  or
##   vcov(x)     if you need it
```

IRELAND

```r
summary(reltime.dep.r[[11]])
```

```
## Linear mixed model fit by REML ['lmerMod']
## Formula: z_reltime ~ logits * z_wler * z_joy * z_meta + logits * z_escs +
##     logits * gender + (1 | user) + (1 | task) + (1 | schoolID)
##    Data: totdata
##
## REML criterion at convergence: 47101.4
##
## Scaled residuals:
##     Min      1Q  Median      3Q     Max
## -7.2241 -0.4542  0.0623  0.5679  6.7081
##
## Random effects:
##  Groups   Name        Variance Std.Dev.
##  user     (Intercept) 0.06698  0.2588
##  schoolID (Intercept) 0.01533  0.1238
##  task     (Intercept) 0.53232  0.7296
##  Residual             0.33854  0.5818
## Number of obs: 25383, groups:  user, 1336; schoolID, 138; task, 29
##
## Fixed effects:
##                              Estimate Std. Error t value
## (Intercept)                  0.058497   0.136562   0.428
## logits                       0.223507   0.129808   1.722
## z_wler                       0.106858   0.010748   9.942
## z_joy                       -0.013426   0.010027  -1.339
## z_meta                       0.012107   0.009935   1.219
## z_escs                      -0.007404   0.009200  -0.805
## gender                      -0.073981   0.019784  -3.739
## logits:z_wler                0.085295   0.004503  18.941
## logits:z_joy                 0.017716   0.004241   4.177
## z_wler:z_joy                -0.037002   0.009687  -3.820
## logits:z_meta                0.020451   0.004241   4.823
## z_wler:z_meta               -0.044622   0.009262  -4.818
## z_joy:z_meta                 0.020508   0.009626   2.130
## logits:z_escs                0.012129   0.003796   3.195
## logits:gender               -0.051209   0.007358  -6.960
## logits:z_wler:z_joy         -0.015207   0.004115  -3.695
## logits:z_wler:z_meta        -0.001348   0.003909  -0.345
## logits:z_joy:z_meta          0.011709   0.004122   2.840
## z_wler:z_joy:z_meta         -0.001710   0.008802  -0.194
## logits:z_wler:z_joy:z_meta -0.011188   0.003766  -2.971

##
## Correlation matrix not shown by default, as p = 20 > 12.
## Use print(x, correlation=TRUE)  or
##   vcov(x)     if you need it
```

ICELAND

```r
summary(reltime.dep.r[[12]])
```

```
## Linear mixed model fit by REML ['lmerMod']
## Formula: z_reltime ~ logits * z_wler * z_joy * z_meta + logits * z_escs +
##     logits * gender + (1 | user) + (1 | task) + (1 | schoolID)
##    Data: totdata
##
## REML criterion at convergence: 31391.8
##
## Scaled residuals:
##     Min      1Q  Median      3Q     Max
## -6.5155 -0.4716  0.0636  0.5727  5.3201
##
## Random effects:
##  Groups   Name        Variance Std.Dev.
##  user     (Intercept) 0.08177  0.2860
##  schoolID (Intercept) 0.03651  0.1911
##  task     (Intercept) 0.50926  0.7136
##  Residual             0.30517  0.5524
## Number of obs: 17706, groups:  user, 930; schoolID, 118; task, 29
##
## Fixed effects:
##                              Estimate Std. Error t value
## (Intercept)                 0.0727291  0.1346775   0.540
## logits                      0.1784689  0.1269983   1.405
## z_wler                      0.0938934  0.0139102   6.750
## z_joy                       0.0187610  0.0134241   1.398
## z_meta                      0.0685132  0.0126794   5.404
## z_escs                      0.0035419  0.0122506   0.289
## gender                     -0.0800009  0.0237055  -3.375
## logits:z_wler               0.0884024  0.0050754  17.418
## logits:z_joy                0.0278401  0.0050364   5.528
## z_wler:z_joy               -0.0147675  0.0126909  -1.164
## logits:z_meta               0.0243258  0.0046860   5.191
## z_wler:z_meta              -0.0415023  0.0132873  -3.123
## z_joy:z_meta                0.0051857  0.0128922   0.402
## logits:z_escs               0.0093826  0.0042026   2.233
## logits:gender              -0.0300063  0.0088396  -3.395
## logits:z_wler:z_joy        -0.0060642  0.0047456  -1.278
## logits:z_wler:z_meta        0.0002083  0.0050166   0.042
## logits:z_joy:z_meta        -0.0135004  0.0048541  -2.781
## z_wler:z_joy:z_meta        -0.0177122  0.0117512  -1.507
## logits:z_wler:z_joy:z_meta -0.0178667  0.0044126  -4.049

##
## Correlation matrix not shown by default, as p = 20 > 12.
## Use print(x, correlation=TRUE)  or
##   vcov(x)     if you need it
```

JAPAN

```
summary(reltime.dep.r[[13]])
```

```
## Linear mixed model fit by REML ['lmerMod']
## Formula: z_reltime ~ logits * z_wler * z_joy * z_meta + logits * z_escs +
##     logits * gender + (1 | user) + (1 | task) + (1 | schoolID)
##    Data: totdata
##
## REML criterion at convergence: 36805.9
##
## Scaled residuals:
##     Min      1Q  Median      3Q     Max
## -7.4050 -0.4401  0.0728  0.5702  6.0162
##
## Random effects:
##  Groups   Name        Variance Std.Dev.
##  user     (Intercept) 0.050038 0.2237
##  schoolID (Intercept) 0.005084 0.0713
##  task     (Intercept) 0.538224 0.7336
##  Residual             0.292944 0.5412
## Number of obs: 21591, groups:  user, 1155; schoolID, 41; task, 29
##
## Fixed effects:
##                               Estimate Std. Error t value
## (Intercept)                  3.755e-02  1.372e-01   0.274
## logits                       3.066e-01  1.305e-01   2.349
## z_wler                       6.208e-02  9.900e-03   6.271
## z_joy                       -3.323e-03  8.839e-03  -0.376
## z_meta                       2.596e-02  9.156e-03   2.835
## z_escs                      -7.010e-03  8.307e-03  -0.844
## gender                      -2.952e-02  1.646e-02  -1.793
## logits:z_wler                5.694e-02  4.404e-03  12.929
## logits:z_joy                 1.138e-02  4.113e-03   2.768
## z_wler:z_joy                -3.004e-02  9.268e-03  -3.241
## logits:z_meta                1.631e-02  4.249e-03   3.837
## z_wler:z_meta               -3.475e-02  7.838e-03  -4.433
## z_joy:z_meta                -9.924e-05  9.167e-03  -0.011
## logits:z_escs                5.402e-04  3.727e-03   0.145
## logits:gender               -3.331e-03  7.328e-03  -0.455
## logits:z_wler:z_joy         -1.151e-02  4.337e-03  -2.654
## logits:z_wler:z_meta         2.403e-03  3.537e-03   0.679
## logits:z_joy:z_meta         -9.094e-03  4.287e-03  -2.121
## z_wler:z_joy:z_meta          1.663e-02  7.508e-03   2.215
## logits:z_wler:z_joy:z_meta  9.616e-03  3.580e-03   2.686

##
## Correlation matrix not shown by default, as p = 20 > 12.
## Use print(x, correlation=TRUE)  or
##   vcov(x)     if you need it
```

KOREA

```r
summary(reltime.dep.r[[14]])
```

```
## Linear mixed model fit by REML ['lmerMod']
## Formula: z_reltime ~ logits * z_wler * z_joy * z_meta + logits * z_escs +
##     logits * gender + (1 | user) + (1 | task) + (1 | schoolID)
##    Data: totdata
##
## REML criterion at convergence: 44291.9
##
## Scaled residuals:
##     Min      1Q  Median      3Q     Max
## -8.5776 -0.5049  0.0356  0.5843  5.0573
##
## Random effects:
##  Groups   Name        Variance Std.Dev.
##  user     (Intercept) 0.048342 0.21987
##  schoolID (Intercept) 0.009676 0.09837
##  task     (Intercept) 0.605911 0.77840
##  Residual             0.262225 0.51208
## Number of obs: 27747, groups:  user, 1452; schoolID, 156; task, 29
##
## Fixed effects:
##                             Estimate Std. Error t value
## (Intercept)                0.0075137  0.1451831   0.052
## logits                     0.3184548  0.1384461   2.300
## z_wler                     0.0702556  0.0086875   8.087
## z_joy                     -0.0136792  0.0079172  -1.728
## z_meta                    -0.0008088  0.0081448  -0.099
## z_escs                    -0.0059644  0.0075862  -0.786
## gender                     0.0207125  0.0160945   1.287
## logits:z_wler              0.0525028  0.0037021  14.182
## logits:z_joy               0.0102025  0.0034752   2.936
## z_wler:z_joy              -0.0257047  0.0080068  -3.210
## logits:z_meta              0.0144125  0.0035535   4.056
## z_wler:z_meta             -0.0269155  0.0074082  -3.633
## z_joy:z_meta               0.0099389  0.0085101   1.168
## logits:z_escs              0.0060148  0.0031621   1.902
## logits:gender             -0.0232316  0.0061515  -3.777
## logits:z_wler:z_joy       -0.0091503  0.0035384  -2.586
## logits:z_wler:z_meta      -0.0080725  0.0032493  -2.484
## logits:z_joy:z_meta       -0.0017260  0.0037257  -0.463
## z_wler:z_joy:z_meta        0.0097852  0.0066612   1.469
## logits:z_wler:z_joy:z_meta -0.0007676  0.0029303  -0.262

##
## Correlation matrix not shown by default, as p = 20 > 12.
## Use print(x, correlation=TRUE)  or
##   vcov(x)     if you need it
```

MACAO-CHINA

```r
summary(reltime.dep.r[[15]])
```

```
## Linear mixed model fit by REML ['lmerMod']
## Formula: z_reltime ~ logits * z_wler * z_joy * z_meta + logits * z_escs +
##     logits * gender + (1 | user) + (1 | task) + (1 | schoolID)
##    Data: totdata
##
## REML criterion at convergence: 79837.5
##
## Scaled residuals:
##     Min      1Q  Median      3Q     Max
## -6.8419 -0.4657  0.0755  0.5971  4.8925
##
## Random effects:
##  Groups   Name        Variance Std.Dev.
##  user     (Intercept) 0.0590   0.2429
##  schoolID (Intercept) 0.0101   0.1005
##  task     (Intercept) 0.5437   0.7374
##  Residual             0.3129   0.5594
## Number of obs: 45159, groups:  user, 2484; schoolID, 44; task, 29
##
## Fixed effects:
##                             Estimate Std. Error t value
## (Intercept)                 0.023531   0.138116   0.170
## logits                      0.282478   0.131133   2.154
## z_wler                      0.064964   0.007027   9.244
## z_joy                       0.004082   0.006505   0.628
## z_meta                      0.020181   0.006319   3.194
## z_escs                     -0.005521   0.006530  -0.845
## gender                     -0.057001   0.013343  -4.272
## logits:z_wler               0.082359   0.002924  28.169
## logits:z_joy                0.010472   0.002959   3.540
## z_wler:z_joy               -0.035497   0.005946  -5.970
## logits:z_meta               0.008193   0.002827   2.899
## z_wler:z_meta              -0.018532   0.005982  -3.098
## z_joy:z_meta                0.010976   0.006033   1.819
## logits:z_escs              -0.004210   0.002636  -1.597
## logits:gender              -0.035217   0.005482  -6.424
## logits:z_wler:z_joy        -0.005182   0.002696  -1.922
## logits:z_wler:z_meta       -0.005293   0.002711  -1.953
## logits:z_joy:z_meta         0.004680   0.002729   1.715
## z_wler:z_joy:z_meta        -0.003488   0.004796  -0.727
## logits:z_wler:z_joy:z_meta  0.000584   0.002158   0.271

##
## Correlation matrix not shown by default, as p = 20 > 12.
## Use print(x, correlation=TRUE)  or
##   vcov(x)     if you need it
```

NORWAY

```r
summary(reltime.dep.r[[16]])
```

```
## Linear mixed model fit by REML ['lmerMod']
## Formula: z_reltime ~ logits * z_wler * z_joy * z_meta + logits * z_escs +
##     logits * gender + (1 | user) + (1 | task) + (1 | schoolID)
##    Data: totdata
##
## REML criterion at convergence: 65906.6
##
## Scaled residuals:
##     Min      1Q  Median      3Q     Max
## -6.9347 -0.4695  0.0699  0.5882  5.4459
##
## Random effects:
##  Groups   Name        Variance Std.Dev.
##  user     (Intercept) 0.09095  0.3016
##  schoolID (Intercept) 0.04201  0.2050
##  task     (Intercept) 0.49699  0.7050
##  Residual             0.32188  0.5673
## Number of obs: 36225, groups:  user, 1902; schoolID, 180; task, 29
##
## Fixed effects:
##                              Estimate Std. Error t value
## (Intercept)                  0.057244   0.132362   0.432
## logits                       0.155333   0.125401   1.239
## z_wler                       0.124379   0.010045  12.382
## z_joy                        0.027718   0.009575   2.895
## z_meta                       0.018637   0.009529   1.956
## z_escs                      -0.017145   0.008464  -2.026
## gender                      -0.046346   0.016521  -2.805
## logits:z_wler                0.088568   0.003661  24.192
## logits:z_joy                 0.020895   0.003484   5.997
## z_wler:z_joy                -0.038098   0.009071  -4.200
## logits:z_meta                0.013133   0.003485   3.769
## z_wler:z_meta               -0.031871   0.008699  -3.664
## z_joy:z_meta                 0.005453   0.009119   0.598
## logits:z_escs                0.008089   0.003011   2.687
## logits:gender               -0.002817   0.006195  -0.455
## logits:z_wler:z_joy          0.005351   0.003340   1.602
## logits:z_wler:z_meta        -0.010576   0.003189  -3.316
## logits:z_joy:z_meta          0.006972   0.003372   2.068
## z_wler:z_joy:z_meta          0.010477   0.007235   1.448
## logits:z_wler:z_joy:z_meta -0.006182   0.002646  -2.337

##
## Correlation matrix not shown by default, as p = 20 > 12.
## Use print(x, correlation=TRUE)  or
##   vcov(x)     if you need it
```

NEW ZEALAND

```
summary(reltime.dep.r[[17]])
```

```
## Linear mixed model fit by REML ['lmerMod']
## Formula: z_reltime ~ logits * z_wler * z_joy * z_meta + logits * z_escs +
##     logits * gender + (1 | user) + (1 | task) + (1 | schoolID)
##    Data: totdata
##
## REML criterion at convergence: 57627.8
##
## Scaled residuals:
##     Min      1Q  Median      3Q     Max
## -8.6919 -0.4483  0.0559  0.5543  5.9463
##
## Random effects:
##  Groups   Name        Variance Std.Dev.
##  user     (Intercept) 0.07202  0.2684
##  schoolID (Intercept) 0.01776  0.1333
##  task     (Intercept) 0.51506  0.7177
##  Residual             0.31920  0.5650
## Number of obs: 32018, groups:  user, 1687; schoolID, 145; task, 29
##
## Fixed effects:
##                            Estimate Std. Error t value
## (Intercept)                 0.082621   0.134302   0.615
## logits                      0.248355   0.127667   1.945
## z_wler                      0.039673   0.010137   3.914
## z_joy                       0.022617   0.009560   2.366
## z_meta                      0.030140   0.009099   3.313
## z_escs                      0.001423   0.008562   0.166
## gender                     -0.084643   0.018097  -4.677
## logits:z_wler               0.083812   0.004145  20.218
## logits:z_joy                0.019575   0.003916   4.999
## z_wler:z_joy               -0.038587   0.008335  -4.629
## logits:z_meta               0.026124   0.003693   7.074
## z_wler:z_meta              -0.054956   0.008651  -6.352
## z_joy:z_meta               -0.007776   0.008692  -0.895
## logits:z_escs               0.014060   0.003347   4.201
## logits:gender              -0.024666   0.006580  -3.748
## logits:z_wler:z_joy        -0.004029   0.003415  -1.180
## logits:z_wler:z_meta       -0.009812   0.003521  -2.787
## logits:z_joy:z_meta        -0.003769   0.003568  -1.056
## z_wler:z_joy:z_meta         0.008354   0.007541   1.108
## logits:z_wler:z_joy:z_meta -0.008195   0.003093  -2.649

##
## Correlation matrix not shown by default, as p = 20 > 12.
## Use print(x, correlation=TRUE)  or
##   vcov(x)     if you need it
```

SWEDEN

```r
summary(reltime.dep.r[[18]])
```

```
## Linear mixed model fit by REML ['lmerMod']
## Formula: z_reltime ~ logits * z_wler * z_joy * z_meta + logits * z_escs +
##     logits * gender + (1 | user) + (1 | task) + (1 | schoolID)
##    Data: totdata
##
## REML criterion at convergence: 60013.5
##
## Scaled residuals:
##     Min      1Q  Median      3Q     Max
## -7.5557 -0.4573  0.0601  0.5658  7.2498
##
## Random effects:
##  Groups   Name        Variance Std.Dev.
##  user     (Intercept) 0.09023  0.3004
##  schoolID (Intercept) 0.01655  0.1286
##  task     (Intercept) 0.51615  0.7184
##  Residual             0.31514  0.5614
## Number of obs: 33405, groups:  user, 1760; schoolID, 179; task, 29
##
## Fixed effects:
##                             Estimate Std. Error t value
## (Intercept)                 0.057478   0.134343   0.428
## logits                      0.180142   0.127795   1.410
## z_wler                      0.099934   0.010909   9.161
## z_joy                       0.033944   0.009941   3.415
## z_meta                      0.036033   0.009873   3.650
## z_escs                      0.013914   0.008960   1.553
## gender                     -0.046800   0.017448  -2.682
## logits:z_wler               0.083891   0.003981  21.074
## logits:z_joy                0.016122   0.003656   4.410
## z_wler:z_joy               -0.059004   0.009217  -6.402
## logits:z_meta               0.024040   0.003554   6.765
## z_wler:z_meta              -0.032187   0.009473  -3.398
## z_joy:z_meta                0.013395   0.009719   1.378
## logits:z_escs               0.014289   0.003251   4.395
## logits:gender              -0.009078   0.006511  -1.394
## logits:z_wler:z_joy        -0.003371   0.003404  -0.990
## logits:z_wler:z_meta       -0.007445   0.003479  -2.140
## logits:z_joy:z_meta         0.006074   0.003582   1.696
## z_wler:z_joy:z_meta        -0.001965   0.007550  -0.260
## logits:z_wler:z_joy:z_meta -0.008223   0.002824  -2.912

##
## Correlation matrix not shown by default, as p = 20 > 12.
## Use print(x, correlation=TRUE)  or
##   vcov(x)     if you need it
```

POLAND

```r
summary(reltime.dep.r[[19]])
```

```
## Linear mixed model fit by REML ['lmerMod']
## Formula: z_reltime ~ logits * z_wler * z_joy * z_meta + logits * z_escs +
##     logits * gender + (1 | user) + (1 | task) + (1 | schoolID)
##    Data: totdata
##
## REML criterion at convergence: 64567.1
##
## Scaled residuals:
##     Min      1Q  Median      3Q     Max
## -7.4776 -0.4517  0.0846  0.5843  6.0711
##
## Random effects:
##  Groups   Name        Variance Std.Dev.
##  user     (Intercept) 0.07727  0.2780
##  schoolID (Intercept) 0.03958  0.1990
##  task     (Intercept) 0.51983  0.7210
##  Residual             0.33562  0.5793
## Number of obs: 34848, groups:  user, 1823; schoolID, 179; task, 29
##
## Fixed effects:
##                             Estimate Std. Error t value
## (Intercept)                0.0345132  0.1352000   0.255
## logits                     0.1280651  0.1282504   0.999
## z_wler                     0.1186544  0.0097934  12.116
## z_joy                      0.0303593  0.0093314   3.253
## z_meta                     0.0219008  0.0086709   2.526
## z_escs                    -0.0072311  0.0090855  -0.796
## gender                    -0.0154860  0.0162692  -0.952
## logits:z_wler              0.0722339  0.0038003  19.008
## logits:z_joy               0.0301760  0.0036981   8.160
## z_wler:z_joy              -0.0440774  0.0089072  -4.949
## logits:z_meta              0.0132562  0.0034573   3.834
## z_wler:z_meta             -0.0353538  0.0086649  -4.080
## z_joy:z_meta               0.0073892  0.0088199   0.838
## logits:z_escs              0.0171354  0.0033031   5.188
## logits:gender             -0.0004848  0.0065622  -0.074
## logits:z_wler:z_joy       -0.0167438  0.0035482  -4.719
## logits:z_wler:z_meta      -0.0027351  0.0034280  -0.798
## logits:z_joy:z_meta        0.0058511  0.0035184   1.663
## z_wler:z_joy:z_meta        0.0048202  0.0078101   0.617
## logits:z_wler:z_joy:z_meta  0.0001627  0.0030763   0.053
##
## Correlation matrix not shown by default, as p = 20 > 12.
## Use print(x, correlation=TRUE)  or
##   vcov(x)     if you need it
```
