# Supplementary material for: The Skilled, the Knowledgeable, and the Motivated: Investigating the Strategic Allocation of Time on Task in a Computer-Based Assessment
Source: Front Psychol. 2019 Jun 27;10:1429. doi: 10.3389/fpsyg.2019.01429 (PMC6660318; doi:10.3389/fpsyg.2019.01429)
Supplement: Supplementary file 3 [file Data_Sheet_3.pdf]

# Supplement 3 to 'The Skilled, the Knowledgeabeable, and the Motivated: Investigating the strategic allocation of time on task in a computer-based assessment'

*Johannes Naumann*

*June 2019*

## Significance of variance components

This supplement presents significance tests (likelihood ration tests) for the random effects for students, tasks, and schools, in this order, for both dependent variables, total time on task, and average time on relevant pages. Variable names are to be read as follows:

Dependent variables:

z_tottime: Total time on task, logarithmised and standardized
z_reltime: Agerage time on relevant pages, logarithmised and standardized

Predictors:

z_wler: Comprehension skill (WLEs), standardized
z_joy: Enjoyment of reading, standardized
z_meta: Knowledge of reading strategies, standardized
logits: Task difficulty, standardized

Control variables:

gender: Gender, Boys = 0, Girls = 1
z_escs: Socio-economic status (ESCS), standardized

# Dependent variable total time on task

## Persons

### AUSTRALIA

```r
anova(time.dep.r[[1]],
      time.dep.r.rp[[1]])
```

```
## refitting model(s) with ML (instead of REML)
```

```
## Data: totdata
## Models:
## time.dep.r.rp[[1]]: z_tottime ~ logits * z_wler * z_joy * z_meta + logits * z_escs +
## time.dep.r.rp[[1]]:     logits * gender + (1 | task) + (1 | schoolID)
## time.dep.r[[1]]: z_tottime ~ logits * z_wler * z_joy * z_meta + logits * z_escs +
## time.dep.r[[1]]:     logits * gender + (1 | user) + (1 | task) + (1 | schoolID)
##                    Df    AIC    BIC logLik deviance  Chisq Chi Df
## time.dep.r.rp[[1]] 23 115315 115519 -57634   115269
## time.dep.r[[1]]    24 107857 108070 -53905   107809 7459.6      1
##                    Pr(>Chisq)
## time.dep.r.rp[[1]]
## time.dep.r[[1]]     < 2.2e-16 ***
## ---
## Signif. codes:  0 '***' 0.001 '**' 0.01 '*' 0.05 '.' 0.1 ' ' 1
```

### AUSTRIA

```r
anova(time.dep.r[[2]],
      time.dep.r.rp[[2]])
```

```
## refitting model(s) with ML (instead of REML)
```

```
## Data: totdata
## Models:
## time.dep.r.rp[[2]]: z_tottime ~ logits * z_wler * z_joy * z_meta + logits * z_escs +
## time.dep.r.rp[[2]]:     logits * gender + (1 | task) + (1 | schoolID)
## time.dep.r[[2]]: z_tottime ~ logits * z_wler * z_joy * z_meta + logits * z_escs +
## time.dep.r[[2]]:     logits * gender + (1 | user) + (1 | task) + (1 | schoolID)
##                    Df    AIC    BIC logLik deviance  Chisq Chi Df
## time.dep.r.rp[[2]] 23 108417 108619 -54186   108371
## time.dep.r[[2]]    24 104546 104756 -52249   104498 3873.5      1
##                    Pr(>Chisq)
## time.dep.r.rp[[2]]
## time.dep.r[[2]]     < 2.2e-16 ***
## ---
## Signif. codes:  0 '***' 0.001 '**' 0.01 '*' 0.05 '.' 0.1 ' ' 1
```

**BELGUIM**

```r
anova(time.dep.r[[3]],
      time.dep.r.rp[[3]])
```

```
## refitting model(s) with ML (instead of REML)

## Data: totdata
## Models:
## time.dep.r.rp[[3]]: z_tottime ~ logits * z_wler * z_joy * z_meta + logits * z_escs +
## time.dep.r.rp[[3]]:     logits * gender + (1 | task) + (1 | schoolID)
## time.dep.r[[3]]: z_tottime ~ logits * z_wler * z_joy * z_meta + logits * z_escs +
## time.dep.r[[3]]:     logits * gender + (1 | user) + (1 | task) + (1 | schoolID)
##                    Df    AIC    BIC logLik deviance  Chisq Chi Df
## time.dep.r.rp[[3]] 23 108653 108857 -54304   108607
## time.dep.r[[3]]    24 104378 104590 -52165   104330 4277.2      1
##                    Pr(>Chisq)
## time.dep.r.rp[[3]]
## time.dep.r[[3]]     < 2.2e-16 ***
## ---
## Signif. codes:  0 '***' 0.001 '**' 0.01 '*' 0.05 '.' 0.1 ' ' 1
```

**CHILE**

```r
anova(time.dep.r[[4]],
      time.dep.r.rp[[4]])
```

```
## refitting model(s) with ML (instead of REML)

## Data: totdata
## Models:
## time.dep.r.rp[[4]]: z_tottime ~ logits * z_wler * z_joy * z_meta + logits * z_escs +
## time.dep.r.rp[[4]]:     logits * gender + (1 | task) + (1 | schoolID)
## time.dep.r[[4]]: z_tottime ~ logits * z_wler * z_joy * z_meta + logits * z_escs +
## time.dep.r[[4]]:     logits * gender + (1 | user) + (1 | task) + (1 | schoolID)
##                    Df   AIC   BIC logLik deviance  Chisq Chi Df Pr(>Chisq)
## time.dep.r.rp[[4]] 23 70247 70437 -35100    70201
## time.dep.r[[4]]    24 67592 67790 -33772    67544 2656.8      1  < 2.2e-16
##
## time.dep.r.rp[[4]]
## time.dep.r[[4]]    ***
## ---
## Signif. codes:  0 '***' 0.001 '**' 0.01 '*' 0.05 '.' 0.1 ' ' 1
```

**COLOMBIA**

```r
anova(time.dep.r[[5]],
      time.dep.r.rp[[5]])
```

```
## refitting model(s) with ML (instead of REML)

## Data: totdata
## Models:
## time.dep.r.rp[[5]]: z_tottime ~ logits * z_wler * z_joy * z_meta + logits * z_escs +
## time.dep.r.rp[[5]]:     logits * gender + (1 | task) + (1 | schoolID)
## time.dep.r[[5]]: z_tottime ~ logits * z_wler * z_joy * z_meta + logits * z_escs +
## time.dep.r[[5]]:     logits * gender + (1 | user) + (1 | task) + (1 | schoolID)
##                     Df   AIC   BIC logLik deviance  Chisq Chi Df Pr(>Chisq)
## time.dep.r.rp[[5]] 23 57926 58111 -28940    57880
## time.dep.r[[5]]    24 56080 56273 -28016    56032 1847.8      1  < 2.2e-16
##
## time.dep.r.rp[[5]]
## time.dep.r[[5]]    ***
## ---
## Signif. codes:  0 '***' 0.001 '**' 0.01 '*' 0.05 '.' 0.1 ' ' 1
```

**DENMARK**

```r
anova(time.dep.r[[6]],
      time.dep.r.rp[[6]])
```

```
## refitting model(s) with ML (instead of REML)

## Data: totdata
## Models:
## time.dep.r.rp[[6]]: z_tottime ~ logits * z_wler * z_joy * z_meta + logits * z_escs +
## time.dep.r.rp[[6]]:     logits * gender + (1 | task) + (1 | schoolID)
## time.dep.r[[6]]: z_tottime ~ logits * z_wler * z_joy * z_meta + logits * z_escs +
## time.dep.r[[6]]:     logits * gender + (1 | user) + (1 | task) + (1 | schoolID)
##                     Df   AIC   BIC logLik deviance  Chisq Chi Df Pr(>Chisq)
## time.dep.r.rp[[6]] 23 52509 52694 -26232    52463
## time.dep.r[[6]]    24 50202 50395 -25077    50154 2308.7      1  < 2.2e-16
##
## time.dep.r.rp[[6]]
## time.dep.r[[6]]    ***
## ---
## Signif. codes:  0 '***' 0.001 '**' 0.01 '*' 0.05 '.' 0.1 ' ' 1
```

**SPAIN**

```r
anova(time.dep.r[[7]],
      time.dep.r.rp[[7]])
```

```
## refitting model(s) with ML (instead of REML)

## Data: totdata
## Models:
## time.dep.r.rp[[7]]: z_tottime ~ logits * z_wler * z_joy * z_meta + logits * z_escs +
## time.dep.r.rp[[7]]:     logits * gender + (1 | task) + (1 | schoolID)
## time.dep.r[[7]]: z_tottime ~ logits * z_wler * z_joy * z_meta + logits * z_escs +
## time.dep.r[[7]]:     logits * gender + (1 | user) + (1 | task) + (1 | schoolID)
##                    Df   AIC   BIC logLik deviance  Chisq Chi Df Pr(>Chisq)
## time.dep.r.rp[[7]] 23 71377 71569 -35665    71331
## time.dep.r[[7]]    24 68756 68957 -34354    68708 2622.2      1  < 2.2e-16
##
## time.dep.r.rp[[7]]
## time.dep.r[[7]]    ***
## ---
## Signif. codes:  0 '***' 0.001 '**' 0.01 '*' 0.05 '.' 0.1 ' ' 1
```

**FRANCE**

```r
anova(time.dep.r[[8]],
      time.dep.r.rp[[8]])
```

```
## refitting model(s) with ML (instead of REML)

## Data: totdata
## Models:
## time.dep.r.rp[[8]]: z_tottime ~ logits * z_wler * z_joy * z_meta + logits * z_escs +
## time.dep.r.rp[[8]]:     logits * gender + (1 | task) + (1 | schoolID)
## time.dep.r[[8]]: z_tottime ~ logits * z_wler * z_joy * z_meta + logits * z_escs +
## time.dep.r[[8]]:     logits * gender + (1 | user) + (1 | task) + (1 | schoolID)
##                    Df   AIC   BIC logLik deviance  Chisq Chi Df Pr(>Chisq)
## time.dep.r.rp[[8]] 23 49658 49843 -24806    49612
## time.dep.r[[8]]    24 48287 48480 -24120    48239 1372.9      1  < 2.2e-16
##
## time.dep.r.rp[[8]]
## time.dep.r[[8]]    ***
## ---
## Signif. codes:  0 '***' 0.001 '**' 0.01 '*' 0.05 '.' 0.1 ' ' 1
```

### HONG KONG-CHINA

```r
anova(time.dep.r[[9]],
      time.dep.r.rp[[9]])
```

```
## refitting model(s) with ML (instead of REML)

## Data: totdata
## Models:
## time.dep.r.rp[[9]]: z_tottime ~ logits * z_wler * z_joy * z_meta + logits * z_escs +
## time.dep.r.rp[[9]]:     logits * gender + (1 | task) + (1 | schoolID)
## time.dep.r[[9]]: z_tottime ~ logits * z_wler * z_joy * z_meta + logits * z_escs +
## time.dep.r[[9]]:     logits * gender + (1 | user) + (1 | task) + (1 | schoolID)
##                    Df   AIC   BIC logLik deviance  Chisq Chi Df Pr(>Chisq)
## time.dep.r.rp[[9]] 23 55739 55927 -27846    55693
## time.dep.r[[9]]    24 52663 52859 -26308    52615 3077.8      1  < 2.2e-16
##
## time.dep.r.rp[[9]]
## time.dep.r[[9]]    ***
## ---
## Signif. codes:  0 '***' 0.001 '**' 0.01 '*' 0.05 '.' 0.1 ' ' 1
```

### HUNGARY

```r
anova(time.dep.r[[10]],
      time.dep.r.rp[[10]])
```

```
## refitting model(s) with ML (instead of REML)

## Data: totdata
## Models:
## time.dep.r.rp[[10]]: z_tottime ~ logits * z_wler * z_joy * z_meta + logits * z_escs +
## time.dep.r.rp[[10]]:     logits * gender + (1 | task) + (1 | schoolID)
## time.dep.r[[10]]: z_tottime ~ logits * z_wler * z_joy * z_meta + logits * z_escs +
## time.dep.r[[10]]:     logits * gender + (1 | user) + (1 | task) + (1 | schoolID)
##                     Df   AIC   BIC logLik deviance  Chisq Chi Df
## time.dep.r.rp[[10]] 23 74265 74457 -37109    74219
## time.dep.r[[10]]    24 71013 71214 -35483    70965 3253.5      1
##                     Pr(>Chisq)
## time.dep.r.rp[[10]]
## time.dep.r[[10]]     < 2.2e-16 ***
## ---
## Signif. codes:  0 '***' 0.001 '**' 0.01 '*' 0.05 '.' 0.1 ' ' 1
```

**IRELAND**

```
anova(time.dep.r[[11]],
      time.dep.r.rp[[11]])
```

```
## refitting model(s) with ML (instead of REML)
```

```
## Data: totdata
## Models:
## time.dep.r.rp[[11]]: z_tottime ~ logits * z_wler * z_joy * z_meta + logits * z_escs +
## time.dep.r.rp[[11]]:     logits * gender + (1 | task) + (1 | schoolID)
## time.dep.r[[11]]: z_tottime ~ logits * z_wler * z_joy * z_meta + logits * z_escs +
## time.dep.r[[11]]:     logits * gender + (1 | user) + (1 | task) + (1 | schoolID)
##                     Df   AIC   BIC logLik deviance  Chisq Chi Df
## time.dep.r.rp[[11]] 23 55917 56104 -27935    55871
## time.dep.r[[11]]    24 53661 53856 -26806    53613 2257.8      1
##                     Pr(>Chisq)
## time.dep.r.rp[[11]]
## time.dep.r[[11]]     < 2.2e-16 ***
## ---
## Signif. codes:  0 '***' 0.001 '**' 0.01 '*' 0.05 '.' 0.1 ' ' 1
```

**ICELAND**

```
anova(time.dep.r[[12]],
      time.dep.r.rp[[12]])
```

```
## refitting model(s) with ML (instead of REML)
```

```
## Data: totdata
## Models:
## time.dep.r.rp[[12]]: z_tottime ~ logits * z_wler * z_joy * z_meta + logits * z_escs +
## time.dep.r.rp[[12]]:     logits * gender + (1 | task) + (1 | schoolID)
## time.dep.r[[12]]: z_tottime ~ logits * z_wler * z_joy * z_meta + logits * z_escs +
## time.dep.r[[12]]:     logits * gender + (1 | user) + (1 | task) + (1 | schoolID)
##                     Df   AIC   BIC logLik deviance  Chisq Chi Df
## time.dep.r.rp[[12]] 23 39020 39199 -19487    38974
## time.dep.r[[12]]    24 36717 36904 -18335    36669 2304.2      1
##                     Pr(>Chisq)
## time.dep.r.rp[[12]]
## time.dep.r[[12]]     < 2.2e-16 ***
## ---
## Signif. codes:  0 '***' 0.001 '**' 0.01 '*' 0.05 '.' 0.1 ' ' 1
```

**JAPAN**

```r
anova(time.dep.r[[13]],
      time.dep.r.rp[[13]])
```

```
## refitting model(s) with ML (instead of REML)

## Data: totdata
## Models:
## time.dep.r.rp[[13]]: z_tottime ~ logits * z_wler * z_joy * z_meta + logits * z_escs +
## time.dep.r.rp[[13]]:     logits * gender + (1 | task) + (1 | schoolID)
## time.dep.r[[13]]: z_tottime ~ logits * z_wler * z_joy * z_meta + logits * z_escs +
## time.dep.r[[13]]:     logits * gender + (1 | user) + (1 | task) + (1 | schoolID)
##                     Df   AIC   BIC logLik deviance  Chisq Chi Df
## time.dep.r.rp[[13]] 23 42819 43003 -21387    42773
## time.dep.r[[13]]    24 41102 41293 -20527    41054 1719.8      1
##                     Pr(>Chisq)
## time.dep.r.rp[[13]]
## time.dep.r[[13]]     < 2.2e-16 ***
## ---
## Signif. codes:  0 '***' 0.001 '**' 0.01 '*' 0.05 '.' 0.1 ' ' 1
```

**KOREA**

```r
anova(time.dep.r[[14]],
      time.dep.r.rp[[14]])
```

```
## refitting model(s) with ML (instead of REML)

## Data: totdata
## Models:
## time.dep.r.rp[[14]]: z_tottime ~ logits * z_wler * z_joy * z_meta + logits * z_escs +
## time.dep.r.rp[[14]]:     logits * gender + (1 | task) + (1 | schoolID)
## time.dep.r[[14]]: z_tottime ~ logits * z_wler * z_joy * z_meta + logits * z_escs +
## time.dep.r[[14]]:     logits * gender + (1 | user) + (1 | task) + (1 | schoolID)
##                     Df   AIC   BIC logLik deviance  Chisq Chi Df
## time.dep.r.rp[[14]] 23 53462 53651 -26708    53416
## time.dep.r[[14]]    24 51268 51466 -25610    51220 2195.6      1
##                     Pr(>Chisq)
## time.dep.r.rp[[14]]
## time.dep.r[[14]]     < 2.2e-16 ***
## ---
## Signif. codes:  0 '***' 0.001 '**' 0.01 '*' 0.05 '.' 0.1 ' ' 1
```

**MACAO-CHINA**

```r
anova(time.dep.r[[15]],
      time.dep.r.rp[[15]])
```

```
## refitting model(s) with ML (instead of REML)

## Data: totdata
## Models:
## time.dep.r.rp[[15]]: z_tottime ~ logits * z_wler * z_joy * z_meta + logits * z_escs +
## time.dep.r.rp[[15]]:     logits * gender + (1 | task) + (1 | schoolID)
## time.dep.r[[15]]: z_tottime ~ logits * z_wler * z_joy * z_meta + logits * z_escs +
## time.dep.r[[15]]:     logits * gender + (1 | user) + (1 | task) + (1 | schoolID)
##                     Df   AIC   BIC logLik deviance Chisq Chi Df Pr(>Chisq)
## time.dep.r.rp[[15]] 23 94257 94458 -47106    94211
## time.dep.r[[15]]    24 90377 90587 -45165    90329  3882      1  < 2.2e-16
##
## time.dep.r.rp[[15]]
## time.dep.r[[15]]    ***
## ---
## Signif. codes:  0 '***' 0.001 '**' 0.01 '*' 0.05 '.' 0.1 ' ' 1
```

**NORWAY**

```r
anova(time.dep.r[[16]],
      time.dep.r.rp[[16]])
```

```
## refitting model(s) with ML (instead of REML)

## Data: totdata
## Models:
## time.dep.r.rp[[16]]: z_tottime ~ logits * z_wler * z_joy * z_meta + logits * z_escs +
## time.dep.r.rp[[16]]:     logits * gender + (1 | task) + (1 | schoolID)
## time.dep.r[[16]]: z_tottime ~ logits * z_wler * z_joy * z_meta + logits * z_escs +
## time.dep.r[[16]]:     logits * gender + (1 | user) + (1 | task) + (1 | schoolID)
##                     Df   AIC   BIC logLik deviance  Chisq Chi Df
## time.dep.r.rp[[16]] 23 81359 81555 -40657    81313
## time.dep.r[[16]]    24 76400 76604 -38176    76352 4961.5      1
##                     Pr(>Chisq)
## time.dep.r.rp[[16]]
## time.dep.r[[16]]     < 2.2e-16 ***
## ---
## Signif. codes:  0 '***' 0.001 '**' 0.01 '*' 0.05 '.' 0.1 ' ' 1
```

**NEW ZEALAND**

```r
anova(time.dep.r[[17]],
      time.dep.r.rp[[17]])
```

```
## refitting model(s) with ML (instead of REML)

## Data: totdata
## Models:
## time.dep.r.rp[[17]]: z_tottime ~ logits * z_wler * z_joy * z_meta + logits * z_escs +
## time.dep.r.rp[[17]]:     logits * gender + (1 | task) + (1 | schoolID)
## time.dep.r[[17]]: z_tottime ~ logits * z_wler * z_joy * z_meta + logits * z_escs +
## time.dep.r[[17]]:     logits * gender + (1 | user) + (1 | task) + (1 | schoolID)
##                     Df   AIC   BIC logLik deviance  Chisq Chi Df
## time.dep.r.rp[[17]] 23 67639 67832 -33797    67593
## time.dep.r[[17]]    24 64214 64415 -32083    64166 3427.3      1
##                     Pr(>Chisq)
## time.dep.r.rp[[17]]
## time.dep.r[[17]]     < 2.2e-16 ***
## ---
## Signif. codes:  0 '***' 0.001 '**' 0.01 '*' 0.05 '.' 0.1 ' ' 1
```

**SWEDEN**

```r
anova(time.dep.r[[18]],
      time.dep.r.rp[[18]])
```

```
## refitting model(s) with ML (instead of REML)

## Data: totdata
## Models:
## time.dep.r.rp[[18]]: z_tottime ~ logits * z_wler * z_joy * z_meta + logits * z_escs +
## time.dep.r.rp[[18]]:     logits * gender + (1 | task) + (1 | schoolID)
## time.dep.r[[18]]: z_tottime ~ logits * z_wler * z_joy * z_meta + logits * z_escs +
## time.dep.r[[18]]:     logits * gender + (1 | user) + (1 | task) + (1 | schoolID)
##                     Df   AIC   BIC logLik deviance  Chisq Chi Df
## time.dep.r.rp[[18]] 23 75080 75274 -37517    75034
## time.dep.r[[18]]    24 70521 70723 -35236    70473 4561.7      1
##                     Pr(>Chisq)
## time.dep.r.rp[[18]]
## time.dep.r[[18]]     < 2.2e-16 ***
## ---
## Signif. codes:  0 '***' 0.001 '**' 0.01 '*' 0.05 '.' 0.1 ' ' 1
```

**POLAND**

```
anova(time.dep.r[[19]],
      time.dep.r.rp[[19]])
```

```
## refitting model(s) with ML (instead of REML)
```

```
## Data: totdata
## Models:
## time.dep.r.rp[[19]]: z_tottime ~ logits * z_wler * z_joy * z_meta + logits * z_escs +
## time.dep.r.rp[[19]]:     logits * gender + (1 | task) + (1 | schoolID)
## time.dep.r[[19]]: z_tottime ~ logits * z_wler * z_joy * z_meta + logits * z_escs +
## time.dep.r[[19]]:     logits * gender + (1 | user) + (1 | task) + (1 | schoolID)
##                     Df   AIC   BIC logLik deviance  Chisq Chi Df
## time.dep.r.rp[[19]] 23 80177 80371 -40065    80131
## time.dep.r[[19]]    24 76143 76346 -38048    76095 4035.6      1
##                     Pr(>Chisq)
## time.dep.r.rp[[19]]
## time.dep.r[[19]]     < 2.2e-16 ***
## ---
## Signif. codes:  0 '***' 0.001 '**' 0.01 '*' 0.05 '.' 0.1 ' ' 1
```

## Tasks

### AUSTRALIA

```r
anova(time.dep.r[[1]],
      time.dep.r.rt[[1]])
```

```
## refitting model(s) with ML (instead of REML)

## Data: totdata
## Models:
## time.dep.r.rt[[1]]: z_tottime ~ logits * z_wler * z_joy * z_meta + logits * z_escs +
## time.dep.r.rt[[1]]:     logits * gender + (1 | user) + (1 | schoolID)
## time.dep.r[[1]]: z_tottime ~ logits * z_wler * z_joy * z_meta + logits * z_escs +
## time.dep.r[[1]]:     logits * gender + (1 | user) + (1 | task) + (1 | schoolID)
##                    Df    AIC    BIC logLik deviance Chisq Chi Df
## time.dep.r.rt[[1]] 23 129368 129572 -64661   129322
## time.dep.r[[1]]    24 107857 108070 -53905   107809 21513      1
##                    Pr(>Chisq)
## time.dep.r.rt[[1]]
## time.dep.r[[1]]     < 2.2e-16 ***
## ---
## Signif. codes:  0 '***' 0.001 '**' 0.01 '*' 0.05 '.' 0.1 ' ' 1
```

### AUSTRIA

```r
anova(time.dep.r[[2]],
      time.dep.r.rt[[2]])
```

```
## refitting model(s) with ML (instead of REML)

## Data: totdata
## Models:
## time.dep.r.rt[[2]]: z_tottime ~ logits * z_wler * z_joy * z_meta + logits * z_escs +
## time.dep.r.rt[[2]]:     logits * gender + (1 | user) + (1 | schoolID)
## time.dep.r[[2]]: z_tottime ~ logits * z_wler * z_joy * z_meta + logits * z_escs +
## time.dep.r[[2]]:     logits * gender + (1 | user) + (1 | task) + (1 | schoolID)
##                    Df    AIC    BIC logLik deviance Chisq Chi Df
## time.dep.r.rt[[2]] 23 115310 115512 -57632   115264
## time.dep.r[[2]]    24 104546 104756 -52249   104498 10766      1
##                    Pr(>Chisq)
## time.dep.r.rt[[2]]
## time.dep.r[[2]]     < 2.2e-16 ***
## ---
## Signif. codes:  0 '***' 0.001 '**' 0.01 '*' 0.05 '.' 0.1 ' ' 1
```

**BELGUIM**

```r
anova(time.dep.r[[3]],
      time.dep.r.rt[[3]])
```

```
## refitting model(s) with ML (instead of REML)

## Data: totdata
## Models:
## time.dep.r.rt[[3]]: z_tottime ~ logits * z_wler * z_joy * z_meta + logits * z_escs +
## time.dep.r.rt[[3]]:     logits * gender + (1 | user) + (1 | schoolID)
## time.dep.r[[3]]: z_tottime ~ logits * z_wler * z_joy * z_meta + logits * z_escs +
## time.dep.r[[3]]:     logits * gender + (1 | user) + (1 | task) + (1 | schoolID)
##                    Df    AIC    BIC logLik deviance Chisq Chi Df
## time.dep.r.rt[[3]] 23 123705 123908 -61829   123659
## time.dep.r[[3]]    24 104378 104590 -52165   104330 19329      1
##                    Pr(>Chisq)
## time.dep.r.rt[[3]]
## time.dep.r[[3]]     < 2.2e-16 ***
## ---
## Signif. codes:  0 '***' 0.001 '**' 0.01 '*' 0.05 '.' 0.1 ' ' 1
```

**CHILE**

```r
anova(time.dep.r[[4]],
      time.dep.r.rt[[4]])
```

```
## refitting model(s) with ML (instead of REML)

## Data: totdata
## Models:
## time.dep.r.rt[[4]]: z_tottime ~ logits * z_wler * z_joy * z_meta + logits * z_escs +
## time.dep.r.rt[[4]]:     logits * gender + (1 | user) + (1 | schoolID)
## time.dep.r[[4]]: z_tottime ~ logits * z_wler * z_joy * z_meta + logits * z_escs +
## time.dep.r[[4]]:     logits * gender + (1 | user) + (1 | task) + (1 | schoolID)
##                    Df   AIC   BIC logLik deviance  Chisq Chi Df Pr(>Chisq)
## time.dep.r.rt[[4]] 23 73574 73764 -36764    73528
## time.dep.r[[4]]    24 67592 67790 -33772    67544 5984.4      1  < 2.2e-16
##
## time.dep.r.rt[[4]]
## time.dep.r[[4]]    ***
## ---
## Signif. codes:  0 '***' 0.001 '**' 0.01 '*' 0.05 '.' 0.1 ' ' 1
```

**COLOMBIA**

```r
anova(time.dep.r[[5]],
      time.dep.r.rt[[5]])
```

```
## refitting model(s) with ML (instead of REML)

## Data: totdata
## Models:
## time.dep.r.rt[[5]]: z_tottime ~ logits * z_wler * z_joy * z_meta + logits * z_escs +
## time.dep.r.rt[[5]]:     logits * gender + (1 | user) + (1 | schoolID)
## time.dep.r[[5]]: z_tottime ~ logits * z_wler * z_joy * z_meta + logits * z_escs +
## time.dep.r[[5]]:     logits * gender + (1 | user) + (1 | task) + (1 | schoolID)
##                    Df   AIC   BIC logLik deviance Chisq Chi Df Pr(>Chisq)
## time.dep.r.rt[[5]] 23 59797 59982 -29876    59751
## time.dep.r[[5]]    24 56080 56273 -28016    56032  3719      1  < 2.2e-16
##
## time.dep.r.rt[[5]]
## time.dep.r[[5]]    ***
## ---
## Signif. codes:  0 '***' 0.001 '**' 0.01 '*' 0.05 '.' 0.1 ' ' 1
```

**DENMARK**

```r
anova(time.dep.r[[6]],
      time.dep.r.rt[[6]])
```

```
## refitting model(s) with ML (instead of REML)

## Data: totdata
## Models:
## time.dep.r.rt[[6]]: z_tottime ~ logits * z_wler * z_joy * z_meta + logits * z_escs +
## time.dep.r.rt[[6]]:     logits * gender + (1 | user) + (1 | schoolID)
## time.dep.r[[6]]: z_tottime ~ logits * z_wler * z_joy * z_meta + logits * z_escs +
## time.dep.r[[6]]:     logits * gender + (1 | user) + (1 | task) + (1 | schoolID)
##                    Df   AIC   BIC logLik deviance  Chisq Chi Df Pr(>Chisq)
## time.dep.r.rt[[6]] 23 57284 57469 -28619    57238
## time.dep.r[[6]]    24 50202 50395 -25077    50154 7084.1      1  < 2.2e-16
##
## time.dep.r.rt[[6]]
## time.dep.r[[6]]    ***
## ---
## Signif. codes:  0 '***' 0.001 '**' 0.01 '*' 0.05 '.' 0.1 ' ' 1
```

**SPAIN**

```r
anova(time.dep.r[[7]],
      time.dep.r.rt[[7]])
```

```
## refitting model(s) with ML (instead of REML)

## Data: totdata
## Models:
## time.dep.r.rt[[7]]: z_tottime ~ logits * z_wler * z_joy * z_meta + logits * z_escs +
## time.dep.r.rt[[7]]:     logits * gender + (1 | user) + (1 | schoolID)
## time.dep.r[[7]]: z_tottime ~ logits * z_wler * z_joy * z_meta + logits * z_escs +
## time.dep.r[[7]]:     logits * gender + (1 | user) + (1 | task) + (1 | schoolID)
##                    Df   AIC   BIC logLik deviance  Chisq Chi Df Pr(>Chisq)
## time.dep.r.rt[[7]] 23 77537 77730 -38746    77491
## time.dep.r[[7]]    24 68756 68957 -34354    68708 8783.1      1  < 2.2e-16
##
## time.dep.r.rt[[7]]
## time.dep.r[[7]]    ***
## ---
## Signif. codes:  0 '***' 0.001 '**' 0.01 '*' 0.05 '.' 0.1 ' ' 1
```

**FRANCE**

```r
anova(time.dep.r[[8]],
      time.dep.r.rt[[8]])
```

```
## refitting model(s) with ML (instead of REML)

## Data: totdata
## Models:
## time.dep.r.rt[[8]]: z_tottime ~ logits * z_wler * z_joy * z_meta + logits * z_escs +
## time.dep.r.rt[[8]]:     logits * gender + (1 | user) + (1 | schoolID)
## time.dep.r[[8]]: z_tottime ~ logits * z_wler * z_joy * z_meta + logits * z_escs +
## time.dep.r[[8]]:     logits * gender + (1 | user) + (1 | task) + (1 | schoolID)
##                    Df   AIC   BIC logLik deviance  Chisq Chi Df Pr(>Chisq)
## time.dep.r.rt[[8]] 23 56860 57045 -28407    56814
## time.dep.r[[8]]    24 48287 48480 -24120    48239 8575.1      1  < 2.2e-16
##
## time.dep.r.rt[[8]]
## time.dep.r[[8]]    ***
## ---
## Signif. codes:  0 '***' 0.001 '**' 0.01 '*' 0.05 '.' 0.1 ' ' 1
```

### HONG KONG-CHINA

```r
anova(time.dep.r[[9]],
      time.dep.r.rt[[9]])
```

```
## refitting model(s) with ML (instead of REML)

## Data: totdata
## Models:
## time.dep.r.rt[[9]]: z_tottime ~ logits * z_wler * z_joy * z_meta + logits * z_escs +
## time.dep.r.rt[[9]]:     logits * gender + (1 | user) + (1 | schoolID)
## time.dep.r[[9]]: z_tottime ~ logits * z_wler * z_joy * z_meta + logits * z_escs +
## time.dep.r[[9]]:     logits * gender + (1 | user) + (1 | task) + (1 | schoolID)
##                    Df   AIC   BIC logLik deviance Chisq Chi Df Pr(>Chisq)
## time.dep.r.rt[[9]] 23 63590 63778 -31772    63544
## time.dep.r[[9]]    24 52663 52859 -26308    52615 10929      1  < 2.2e-16
##
## time.dep.r.rt[[9]]
## time.dep.r[[9]]    ***
## ---
## Signif. codes:  0 '***' 0.001 '**' 0.01 '*' 0.05 '.' 0.1 ' ' 1
```

### HUNGARY

```r
anova(time.dep.r[[10]],
      time.dep.r.rt[[10]])
```

```
## refitting model(s) with ML (instead of REML)

## Data: totdata
## Models:
## time.dep.r.rt[[10]]: z_tottime ~ logits * z_wler * z_joy * z_meta + logits * z_escs +
## time.dep.r.rt[[10]]:     logits * gender + (1 | user) + (1 | schoolID)
## time.dep.r[[10]]: z_tottime ~ logits * z_wler * z_joy * z_meta + logits * z_escs +
## time.dep.r[[10]]:     logits * gender + (1 | user) + (1 | task) + (1 | schoolID)
##                     Df   AIC   BIC logLik deviance  Chisq Chi Df
## time.dep.r.rt[[10]] 23 79858 80051 -39906    79812
## time.dep.r[[10]]    24 71013 71214 -35483    70965 8846.8      1
##                     Pr(>Chisq)
## time.dep.r.rt[[10]]
## time.dep.r[[10]]     < 2.2e-16 ***
## ---
## Signif. codes:  0 '***' 0.001 '**' 0.01 '*' 0.05 '.' 0.1 ' ' 1
```

**IRELAND**

```
anova(time.dep.r[[11]],
      time.dep.r.rt[[11]])
```

```
## refitting model(s) with ML (instead of REML)
```

```
## Data: totdata
## Models:
## time.dep.r.rt[[11]]: z_tottime ~ logits * z_wler * z_joy * z_meta + logits * z_escs +
## time.dep.r.rt[[11]]:     logits * gender + (1 | user) + (1 | schoolID)
## time.dep.r[[11]]: z_tottime ~ logits * z_wler * z_joy * z_meta + logits * z_escs +
## time.dep.r[[11]]:     logits * gender + (1 | user) + (1 | task) + (1 | schoolID)
##                     Df   AIC   BIC logLik deviance  Chisq Chi Df
## time.dep.r.rt[[11]] 23 62952 63139 -31453    62906
## time.dep.r[[11]]    24 53661 53856 -26806    53613 9293.3      1
##                     Pr(>Chisq)
## time.dep.r.rt[[11]]
## time.dep.r[[11]]     < 2.2e-16 ***
## ---
## Signif. codes:  0 '***' 0.001 '**' 0.01 '*' 0.05 '.' 0.1 ' ' 1
```

**ICELAND**

```
anova(time.dep.r[[12]],
      time.dep.r.rt[[12]])
```

```
## refitting model(s) with ML (instead of REML)
```

```
## Data: totdata
## Models:
## time.dep.r.rt[[12]]: z_tottime ~ logits * z_wler * z_joy * z_meta + logits * z_escs +
## time.dep.r.rt[[12]]:     logits * gender + (1 | user) + (1 | schoolID)
## time.dep.r[[12]]: z_tottime ~ logits * z_wler * z_joy * z_meta + logits * z_escs +
## time.dep.r[[12]]:     logits * gender + (1 | user) + (1 | task) + (1 | schoolID)
##                     Df   AIC   BIC logLik deviance  Chisq Chi Df
## time.dep.r.rt[[12]] 23 43138 43317 -21546    43092
## time.dep.r[[12]]    24 36717 36904 -18335    36669 6422.1      1
##                     Pr(>Chisq)
## time.dep.r.rt[[12]]
## time.dep.r[[12]]     < 2.2e-16 ***
## ---
## Signif. codes:  0 '***' 0.001 '**' 0.01 '*' 0.05 '.' 0.1 ' ' 1
```

**JAPAN**

```r
anova(time.dep.r[[13]],
      time.dep.r.rt[[13]])
```

```
## refitting model(s) with ML (instead of REML)

## Data: totdata
## Models:
## time.dep.r.rt[[13]]: z_tottime ~ logits * z_wler * z_joy * z_meta + logits * z_escs +
## time.dep.r.rt[[13]]:     logits * gender + (1 | user) + (1 | schoolID)
## time.dep.r[[13]]: z_tottime ~ logits * z_wler * z_joy * z_meta + logits * z_escs +
## time.dep.r[[13]]:     logits * gender + (1 | user) + (1 | task) + (1 | schoolID)
##                     Df   AIC   BIC logLik deviance Chisq Chi Df Pr(>Chisq)
## time.dep.r.rt[[13]] 23 52082 52266 -26018    52036
## time.dep.r[[13]]    24 41102 41293 -20527    41054 10982      1  < 2.2e-16
##
## time.dep.r.rt[[13]]
## time.dep.r[[13]]    ***
## ---
## Signif. codes:  0 '***' 0.001 '**' 0.01 '*' 0.05 '.' 0.1 ' ' 1
```

**KOREA**

```r
anova(time.dep.r[[14]],
      time.dep.r.rt[[14]])
```

```
## refitting model(s) with ML (instead of REML)

## Data: totdata
## Models:
## time.dep.r.rt[[14]]: z_tottime ~ logits * z_wler * z_joy * z_meta + logits * z_escs +
## time.dep.r.rt[[14]]:     logits * gender + (1 | user) + (1 | schoolID)
## time.dep.r[[14]]: z_tottime ~ logits * z_wler * z_joy * z_meta + logits * z_escs +
## time.dep.r[[14]]:     logits * gender + (1 | user) + (1 | task) + (1 | schoolID)
##                     Df   AIC   BIC logLik deviance Chisq Chi Df Pr(>Chisq)
## time.dep.r.rt[[14]] 23 66667 66857 -33311    66621
## time.dep.r[[14]]    24 51268 51466 -25610    51220 15401      1  < 2.2e-16
##
## time.dep.r.rt[[14]]
## time.dep.r[[14]]    ***
## ---
## Signif. codes:  0 '***' 0.001 '**' 0.01 '*' 0.05 '.' 0.1 ' ' 1
```

### MACAO-CHINA

```r
anova(time.dep.r[[15]],
      time.dep.r.rt[[15]])
```

```
## refitting model(s) with ML (instead of REML)
```

```
## Data: totdata
## Models:
## time.dep.r.rt[[15]]: z_tottime ~ logits * z_wler * z_joy * z_meta + logits * z_escs +
## time.dep.r.rt[[15]]:     logits * gender + (1 | user) + (1 | schoolID)
## time.dep.r[[15]]: z_tottime ~ logits * z_wler * z_joy * z_meta + logits * z_escs +
## time.dep.r[[15]]:     logits * gender + (1 | user) + (1 | task) + (1 | schoolID)
##                     Df    AIC    BIC logLik deviance Chisq Chi Df
## time.dep.r.rt[[15]] 23 110340 110540 -55147   110294
## time.dep.r[[15]]    24  90377  90587 -45165    90329 19965      1
##                     Pr(>Chisq)
## time.dep.r.rt[[15]]
## time.dep.r[[15]]     < 2.2e-16 ***
## ---
## Signif. codes:  0 '***' 0.001 '**' 0.01 '*' 0.05 '.' 0.1 ' ' 1
```

### NORWAY

```r
anova(time.dep.r[[16]],
      time.dep.r.rt[[16]])
```

```
## refitting model(s) with ML (instead of REML)
```

```
## Data: totdata
## Models:
## time.dep.r.rt[[16]]: z_tottime ~ logits * z_wler * z_joy * z_meta + logits * z_escs +
## time.dep.r.rt[[16]]:     logits * gender + (1 | user) + (1 | schoolID)
## time.dep.r[[16]]: z_tottime ~ logits * z_wler * z_joy * z_meta + logits * z_escs +
## time.dep.r[[16]]:     logits * gender + (1 | user) + (1 | task) + (1 | schoolID)
##                     Df   AIC   BIC logLik deviance Chisq Chi Df Pr(>Chisq)
## time.dep.r.rt[[16]] 23 89172 89368 -44563    89126
## time.dep.r[[16]]    24 76400 76604 -38176    76352 12774      1  < 2.2e-16
##
## time.dep.r.rt[[16]]
## time.dep.r[[16]]    ***
## ---
## Signif. codes:  0 '***' 0.001 '**' 0.01 '*' 0.05 '.' 0.1 ' ' 1
```

**NEW ZEALAND**

```
anova(time.dep.r[[17]],
      time.dep.r.rt[[17]])
```

```
## refitting model(s) with ML (instead of REML)
```

```
## Data: totdata
## Models:
## time.dep.r.rt[[17]]: z_tottime ~ logits * z_wler * z_joy * z_meta + logits * z_escs +
## time.dep.r.rt[[17]]:     logits * gender + (1 | user) + (1 | schoolID)
## time.dep.r[[17]]: z_tottime ~ logits * z_wler * z_joy * z_meta + logits * z_escs +
## time.dep.r[[17]]:     logits * gender + (1 | user) + (1 | task) + (1 | schoolID)
##                     Df   AIC   BIC logLik deviance Chisq Chi Df Pr(>Chisq)
## time.dep.r.rt[[17]] 23 77782 77975 -38868    77736
## time.dep.r[[17]]    24 64214 64415 -32083    64166 13570      1  < 2.2e-16
##
## time.dep.r.rt[[17]]
## time.dep.r[[17]]    ***
## ---
## Signif. codes:  0 '***' 0.001 '**' 0.01 '*' 0.05 '.' 0.1 ' ' 1
```

**SWEDEN**

```
anova(time.dep.r[[18]],
      time.dep.r.rt[[18]])
```

```
## refitting model(s) with ML (instead of REML)
```

```
## Data: totdata
## Models:
## time.dep.r.rt[[18]]: z_tottime ~ logits * z_wler * z_joy * z_meta + logits * z_escs +
## time.dep.r.rt[[18]]:     logits * gender + (1 | user) + (1 | schoolID)
## time.dep.r[[18]]: z_tottime ~ logits * z_wler * z_joy * z_meta + logits * z_escs +
## time.dep.r[[18]]:     logits * gender + (1 | user) + (1 | task) + (1 | schoolID)
##                     Df   AIC   BIC logLik deviance Chisq Chi Df Pr(>Chisq)
## time.dep.r.rt[[18]] 23 82358 82552 -41156    82312
## time.dep.r[[18]]    24 70521 70723 -35236    70473 11840      1  < 2.2e-16
##
## time.dep.r.rt[[18]]
## time.dep.r[[18]]    ***
## ---
## Signif. codes:  0 '***' 0.001 '**' 0.01 '*' 0.05 '.' 0.1 ' ' 1
```

**POLAND**

```
anova(time.dep.r[[19]],
      time.dep.r.rt[[19]])
```

```
## refitting model(s) with ML (instead of REML)
```

```
## Data: totdata
## Models:
## time.dep.r.rt[[19]]: z_tottime ~ logits * z_wler * z_joy * z_meta + logits * z_escs +
## time.dep.r.rt[[19]]:     logits * gender + (1 | user) + (1 | schoolID)
## time.dep.r[[19]]: z_tottime ~ logits * z_wler * z_joy * z_meta + logits * z_escs +
## time.dep.r[[19]]:     logits * gender + (1 | user) + (1 | task) + (1 | schoolID)
##                     Df   AIC   BIC logLik deviance  Chisq Chi Df
## time.dep.r.rt[[19]] 23 85894 86088 -42924    85848
## time.dep.r[[19]]    24 76143 76346 -38048    76095 9752.4      1
##                     Pr(>Chisq)
## time.dep.r.rt[[19]]
## time.dep.r[[19]]     < 2.2e-16 ***
## ---
## Signif. codes:  0 '***' 0.001 '**' 0.01 '*' 0.05 '.' 0.1 ' ' 1
```

## Schools

### AUSTRALIA

```
anova(time.dep.r[[1]],
      time.dep.r.rs[[1]])
```

```
## refitting model(s) with ML (instead of REML)
```

```
## Data: totdata
## Models:
## time.dep.r.rs[[1]]: z_tottime ~ logits * z_wler * z_joy * z_meta + logits * z_escs +
## time.dep.r.rs[[1]]:     logits * gender + (1 | user) + (1 | task)
## time.dep.r[[1]]: z_tottime ~ logits * z_wler * z_joy * z_meta + logits * z_escs +
## time.dep.r[[1]]:     logits * gender + (1 | user) + (1 | task) + (1 | schoolID)
##                    Df    AIC    BIC logLik deviance  Chisq Chi Df
## time.dep.r.rs[[1]] 23 108084 108289 -54019   108038
## time.dep.r[[1]]    24 107857 108070 -53905   107809 229.06      1
##                    Pr(>Chisq)
## time.dep.r.rs[[1]]
## time.dep.r[[1]]     < 2.2e-16 ***
## ---
## Signif. codes:  0 '***' 0.001 '**' 0.01 '*' 0.05 '.' 0.1 ' ' 1
```

### AUSTRIA

```
anova(time.dep.r[[2]],
      time.dep.r.rs[[2]])
```

```
## refitting model(s) with ML (instead of REML)
```

```
## Data: totdata
## Models:
## time.dep.r.rs[[2]]: z_tottime ~ logits * z_wler * z_joy * z_meta + logits * z_escs +
## time.dep.r.rs[[2]]:     logits * gender + (1 | user) + (1 | task)
## time.dep.r[[2]]: z_tottime ~ logits * z_wler * z_joy * z_meta + logits * z_escs +
## time.dep.r[[2]]:     logits * gender + (1 | user) + (1 | task) + (1 | schoolID)
##                    Df    AIC    BIC logLik deviance  Chisq Chi Df
## time.dep.r.rs[[2]] 23 105612 105813 -52783   105566
## time.dep.r[[2]]    24 104546 104756 -52249   104498 1067.5      1
##                    Pr(>Chisq)
## time.dep.r.rs[[2]]
## time.dep.r[[2]]     < 2.2e-16 ***
## ---
## Signif. codes:  0 '***' 0.001 '**' 0.01 '*' 0.05 '.' 0.1 ' ' 1
```

**BELGUIM**

```
anova(time.dep.r[[3]],
      time.dep.r.rs[[3]])
```

```
## refitting model(s) with ML (instead of REML)
```

```
## Data: totdata
## Models:
## time.dep.r.rs[[3]]: z_tottime ~ logits * z_wler * z_joy * z_meta + logits * z_escs +
## time.dep.r.rs[[3]]:     logits * gender + (1 | user) + (1 | task)
## time.dep.r[[3]]: z_tottime ~ logits * z_wler * z_joy * z_meta + logits * z_escs +
## time.dep.r[[3]]:     logits * gender + (1 | user) + (1 | task) + (1 | schoolID)
##                    Df    AIC    BIC logLik deviance  Chisq Chi Df
## time.dep.r.rs[[3]] 23 104684 104888 -52319   104638
## time.dep.r[[3]]    24 104378 104590 -52165   104330 308.05      1
##                    Pr(>Chisq)
## time.dep.r.rs[[3]]
## time.dep.r[[3]]     < 2.2e-16 ***
## ---
## Signif. codes:  0 '***' 0.001 '**' 0.01 '*' 0.05 '.' 0.1 ' ' 1
```

**CHILE**

```
anova(time.dep.r[[4]],
      time.dep.r.rs[[4]])
```

```
## refitting model(s) with ML (instead of REML)
```

```
## Data: totdata
## Models:
## time.dep.r.rs[[4]]: z_tottime ~ logits * z_wler * z_joy * z_meta + logits * z_escs +
## time.dep.r.rs[[4]]:     logits * gender + (1 | user) + (1 | task)
## time.dep.r[[4]]: z_tottime ~ logits * z_wler * z_joy * z_meta + logits * z_escs +
## time.dep.r[[4]]:     logits * gender + (1 | user) + (1 | task) + (1 | schoolID)
##                    Df   AIC   BIC logLik deviance Chisq Chi Df Pr(>Chisq)
## time.dep.r.rs[[4]] 23 67827 68017 -33890    67781
## time.dep.r[[4]]    24 67592 67790 -33772    67544 236.8      1  < 2.2e-16
##
## time.dep.r.rs[[4]]
## time.dep.r[[4]]    ***
## ---
## Signif. codes:  0 '***' 0.001 '**' 0.01 '*' 0.05 '.' 0.1 ' ' 1
```

**COLOMBIA**

```
anova(time.dep.r[[5]],
      time.dep.r.rs[[5]])
```

```
## refitting model(s) with ML (instead of REML)
```

```
## Data: totdata
## Models:
## time.dep.r.rs[[5]]: z_tottime ~ logits * z_wler * z_joy * z_meta + logits * z_escs +
## time.dep.r.rs[[5]]:     logits * gender + (1 | user) + (1 | task)
## time.dep.r[[5]]: z_tottime ~ logits * z_wler * z_joy * z_meta + logits * z_escs +
## time.dep.r[[5]]:     logits * gender + (1 | user) + (1 | task) + (1 | schoolID)
##                    Df   AIC   BIC logLik deviance  Chisq Chi Df Pr(>Chisq)
## time.dep.r.rs[[5]] 23 56278 56463 -28116    56232
## time.dep.r[[5]]    24 56080 56273 -28016    56032 199.76      1  < 2.2e-16
##
## time.dep.r.rs[[5]]
## time.dep.r[[5]]    ***
## ---
## Signif. codes:  0 '***' 0.001 '**' 0.01 '*' 0.05 '.' 0.1 ' ' 1
```

**DENMARK**

```
anova(time.dep.r[[6]],
      time.dep.r.rs[[6]])
```

```
## refitting model(s) with ML (instead of REML)
```

```
## Data: totdata
## Models:
## time.dep.r.rs[[6]]: z_tottime ~ logits * z_wler * z_joy * z_meta + logits * z_escs +
## time.dep.r.rs[[6]]:     logits * gender + (1 | user) + (1 | task)
## time.dep.r[[6]]: z_tottime ~ logits * z_wler * z_joy * z_meta + logits * z_escs +
## time.dep.r[[6]]:     logits * gender + (1 | user) + (1 | task) + (1 | schoolID)
##                    Df   AIC   BIC logLik deviance  Chisq Chi Df Pr(>Chisq)
## time.dep.r.rs[[6]] 23 50396 50581 -25175    50350
## time.dep.r[[6]]    24 50202 50395 -25077    50154 195.95      1  < 2.2e-16
##
## time.dep.r.rs[[6]]
## time.dep.r[[6]]    ***
## ---
## Signif. codes:  0 '***' 0.001 '**' 0.01 '*' 0.05 '.' 0.1 ' ' 1
```

**SPAIN**

```r
anova(time.dep.r[[7]],
      time.dep.r.rs[[7]])
```

```
## refitting model(s) with ML (instead of REML)
```

```
## Data: totdata
## Models:
## time.dep.r.rs[[7]]: z_tottime ~ logits * z_wler * z_joy * z_meta + logits * z_escs +
## time.dep.r.rs[[7]]:     logits * gender + (1 | user) + (1 | task)
## time.dep.r[[7]]: z_tottime ~ logits * z_wler * z_joy * z_meta + logits * z_escs +
## time.dep.r[[7]]:     logits * gender + (1 | user) + (1 | task) + (1 | schoolID)
##                    Df   AIC   BIC logLik deviance  Chisq Chi Df Pr(>Chisq)
## time.dep.r.rs[[7]] 23 69227 69419 -34590    69181
## time.dep.r[[7]]    24 68756 68957 -34354    68708 472.42      1  < 2.2e-16
##
## time.dep.r.rs[[7]]
## time.dep.r[[7]]    ***
## ---
## Signif. codes:  0 '***' 0.001 '**' 0.01 '*' 0.05 '.' 0.1 ' ' 1
```

**FRANCE**

```r
anova(time.dep.r[[8]],
      time.dep.r.rs[[8]])
```

```
## refitting model(s) with ML (instead of REML)
```

```
## Data: totdata
## Models:
## time.dep.r.rs[[8]]: z_tottime ~ logits * z_wler * z_joy * z_meta + logits * z_escs +
## time.dep.r.rs[[8]]:     logits * gender + (1 | user) + (1 | task)
## time.dep.r[[8]]: z_tottime ~ logits * z_wler * z_joy * z_meta + logits * z_escs +
## time.dep.r[[8]]:     logits * gender + (1 | user) + (1 | task) + (1 | schoolID)
##                    Df   AIC   BIC logLik deviance  Chisq Chi Df Pr(>Chisq)
## time.dep.r.rs[[8]] 23 48511 48696 -24232    48465
## time.dep.r[[8]]    24 48287 48480 -24120    48239 225.66      1  < 2.2e-16
##
## time.dep.r.rs[[8]]
## time.dep.r[[8]]    ***
## ---
## Signif. codes:  0 '***' 0.001 '**' 0.01 '*' 0.05 '.' 0.1 ' ' 1
```

**HONG KONG-CHINA**

```r
anova(time.dep.r[[9]],
      time.dep.r.rs[[9]])
```

```
## refitting model(s) with ML (instead of REML)

## Data: totdata
## Models:
## time.dep.r.rs[[9]]: z_tottime ~ logits * z_wler * z_joy * z_meta + logits * z_escs +
## time.dep.r.rs[[9]]:     logits * gender + (1 | user) + (1 | task)
## time.dep.r[[9]]: z_tottime ~ logits * z_wler * z_joy * z_meta + logits * z_escs +
## time.dep.r[[9]]:     logits * gender + (1 | user) + (1 | task) + (1 | schoolID)
##                    Df   AIC   BIC logLik deviance  Chisq Chi Df Pr(>Chisq)
## time.dep.r.rs[[9]] 23 52752 52940 -26353    52706
## time.dep.r[[9]]    24 52663 52859 -26308    52615 90.626      1  < 2.2e-16
##
## time.dep.r.rs[[9]]
## time.dep.r[[9]]    ***
## ---
## Signif. codes:  0 '***' 0.001 '**' 0.01 '*' 0.05 '.' 0.1 ' ' 1
```

**HUNGARY**

```r
anova(time.dep.r[[10]],
      time.dep.r.rs[[10]])
```

```
## refitting model(s) with ML (instead of REML)

## Data: totdata
## Models:
## time.dep.r.rs[[10]]: z_tottime ~ logits * z_wler * z_joy * z_meta + logits * z_escs +
## time.dep.r.rs[[10]]:     logits * gender + (1 | user) + (1 | task)
## time.dep.r[[10]]: z_tottime ~ logits * z_wler * z_joy * z_meta + logits * z_escs +
## time.dep.r[[10]]:     logits * gender + (1 | user) + (1 | task) + (1 | schoolID)
##                     Df   AIC   BIC logLik deviance  Chisq Chi Df
## time.dep.r.rs[[10]] 23 71384 71576 -35669    71338
## time.dep.r[[10]]    24 71013 71214 -35483    70965 372.51      1
##                     Pr(>Chisq)
## time.dep.r.rs[[10]]
## time.dep.r[[10]]     < 2.2e-16 ***
## ---
## Signif. codes:  0 '***' 0.001 '**' 0.01 '*' 0.05 '.' 0.1 ' ' 1
```

### IRELAND

```r
anova(time.dep.r[[11]],
      time.dep.r.rs[[11]])
```

```
## refitting model(s) with ML (instead of REML)
```

```
## Data: totdata
## Models:
## time.dep.r.rs[[11]]: z_tottime ~ logits * z_wler * z_joy * z_meta + logits * z_escs +
## time.dep.r.rs[[11]]:     logits * gender + (1 | user) + (1 | task)
## time.dep.r[[11]]: z_tottime ~ logits * z_wler * z_joy * z_meta + logits * z_escs +
## time.dep.r[[11]]:     logits * gender + (1 | user) + (1 | task) + (1 | schoolID)
##                     Df   AIC   BIC logLik deviance  Chisq Chi Df
## time.dep.r.rs[[11]] 23 53740 53928 -26847    53694
## time.dep.r[[11]]    24 53661 53856 -26806    53613 81.381      1
##                     Pr(>Chisq)
## time.dep.r.rs[[11]]
## time.dep.r[[11]]     < 2.2e-16 ***
## ---
## Signif. codes:  0 '***' 0.001 '**' 0.01 '*' 0.05 '.' 0.1 ' ' 1
```

### ICELAND

```r
anova(time.dep.r[[12]],
      time.dep.r.rs[[12]])
```

```
## refitting model(s) with ML (instead of REML)
```

```
## Data: totdata
## Models:
## time.dep.r.rs[[12]]: z_tottime ~ logits * z_wler * z_joy * z_meta + logits * z_escs +
## time.dep.r.rs[[12]]:     logits * gender + (1 | user) + (1 | task)
## time.dep.r[[12]]: z_tottime ~ logits * z_wler * z_joy * z_meta + logits * z_escs +
## time.dep.r[[12]]:     logits * gender + (1 | user) + (1 | task) + (1 | schoolID)
##                     Df   AIC   BIC logLik deviance  Chisq Chi Df
## time.dep.r.rs[[12]] 23 36857 37036 -18406    36811
## time.dep.r[[12]]    24 36717 36904 -18335    36669 141.51      1
##                     Pr(>Chisq)
## time.dep.r.rs[[12]]
## time.dep.r[[12]]     < 2.2e-16 ***
## ---
## Signif. codes:  0 '***' 0.001 '**' 0.01 '*' 0.05 '.' 0.1 ' ' 1
```

### JAPAN

```
anova(time.dep.r[[13]],
      time.dep.r.rs[[13]])
```

```
## refitting model(s) with ML (instead of REML)
```

```
## Data: totdata
## Models:
## time.dep.r.rs[[13]]: z_tottime ~ logits * z_wler * z_joy * z_meta + logits * z_escs +
## time.dep.r.rs[[13]]:     logits * gender + (1 | user) + (1 | task)
## time.dep.r[[13]]: z_tottime ~ logits * z_wler * z_joy * z_meta + logits * z_escs +
## time.dep.r[[13]]:     logits * gender + (1 | user) + (1 | task) + (1 | schoolID)
##                     Df   AIC   BIC logLik deviance  Chisq Chi Df
## time.dep.r.rs[[13]] 23 41133 41316 -20543    41087
## time.dep.r[[13]]    24 41102 41293 -20527    41054 33.216      1
##                     Pr(>Chisq)
## time.dep.r.rs[[13]]
## time.dep.r[[13]]     8.247e-09 ***
## ---
## Signif. codes:  0 '***' 0.001 '**' 0.01 '*' 0.05 '.' 0.1 ' ' 1
```

### KOREA

```
anova(time.dep.r[[14]],
      time.dep.r.rs[[14]])
```

```
## refitting model(s) with ML (instead of REML)
```

```
## Data: totdata
## Models:
## time.dep.r.rs[[14]]: z_tottime ~ logits * z_wler * z_joy * z_meta + logits * z_escs +
## time.dep.r.rs[[14]]:     logits * gender + (1 | user) + (1 | task)
## time.dep.r[[14]]: z_tottime ~ logits * z_wler * z_joy * z_meta + logits * z_escs +
## time.dep.r[[14]]:     logits * gender + (1 | user) + (1 | task) + (1 | schoolID)
##                     Df   AIC   BIC logLik deviance  Chisq Chi Df
## time.dep.r.rs[[14]] 23 51349 51538 -25651    51303
## time.dep.r[[14]]    24 51268 51466 -25610    51220 82.647      1
##                     Pr(>Chisq)
## time.dep.r.rs[[14]]
## time.dep.r[[14]]     < 2.2e-16 ***
## ---
## Signif. codes:  0 '***' 0.001 '**' 0.01 '*' 0.05 '.' 0.1 ' ' 1
```

**MACAO-CHINA**

```
anova(time.dep.r[[15]],
      time.dep.r.rs[[15]])
```

```
## refitting model(s) with ML (instead of REML)
```

```
## Data: totdata
## Models:
## time.dep.r.rs[[15]]: z_tottime ~ logits * z_wler * z_joy * z_meta + logits * z_escs +
## time.dep.r.rs[[15]]:     logits * gender + (1 | user) + (1 | task)
## time.dep.r[[15]]: z_tottime ~ logits * z_wler * z_joy * z_meta + logits * z_escs +
## time.dep.r[[15]]:     logits * gender + (1 | user) + (1 | task) + (1 | schoolID)
##                     Df   AIC   BIC logLik deviance Chisq Chi Df Pr(>Chisq)
## time.dep.r.rs[[15]] 23 90594 90795 -45274    90548
## time.dep.r[[15]]    24 90377 90587 -45165    90329 218.7      1  < 2.2e-16
##
## time.dep.r.rs[[15]]
## time.dep.r[[15]]    ***
## ---
## Signif. codes:  0 '***' 0.001 '**' 0.01 '*' 0.05 '.' 0.1 ' ' 1
```

**NORWAY**

```
anova(time.dep.r[[16]],
      time.dep.r.rs[[16]])
```

```
## refitting model(s) with ML (instead of REML)
```

```
## Data: totdata
## Models:
## time.dep.r.rs[[16]]: z_tottime ~ logits * z_wler * z_joy * z_meta + logits * z_escs +
## time.dep.r.rs[[16]]:     logits * gender + (1 | user) + (1 | task)
## time.dep.r[[16]]: z_tottime ~ logits * z_wler * z_joy * z_meta + logits * z_escs +
## time.dep.r[[16]]:     logits * gender + (1 | user) + (1 | task) + (1 | schoolID)
##                     Df   AIC   BIC logLik deviance  Chisq Chi Df
## time.dep.r.rs[[16]] 23 76735 76930 -38344    76689
## time.dep.r[[16]]    24 76400 76604 -38176    76352 337.01      1
##                     Pr(>Chisq)
## time.dep.r.rs[[16]]
## time.dep.r[[16]]     < 2.2e-16 ***
## ---
## Signif. codes:  0 '***' 0.001 '**' 0.01 '*' 0.05 '.' 0.1 ' ' 1
```

**NEW ZEALAND**

```r
anova(time.dep.r[[17]],
      time.dep.r.rs[[17]])
```

```
## refitting model(s) with ML (instead of REML)

## Data: totdata
## Models:
## time.dep.r.rs[[17]]: z_tottime ~ logits * z_wler * z_joy * z_meta + logits * z_escs +
## time.dep.r.rs[[17]]:     logits * gender + (1 | user) + (1 | task)
## time.dep.r[[17]]: z_tottime ~ logits * z_wler * z_joy * z_meta + logits * z_escs +
## time.dep.r[[17]]:     logits * gender + (1 | user) + (1 | task) + (1 | schoolID)
##                     Df   AIC   BIC logLik deviance  Chisq Chi Df
## time.dep.r.rs[[17]] 23 64343 64536 -32149    64297
## time.dep.r[[17]]    24 64214 64415 -32083    64166 131.67      1
##                     Pr(>Chisq)
## time.dep.r.rs[[17]]
## time.dep.r[[17]]     < 2.2e-16 ***
## ---
## Signif. codes:  0 '***' 0.001 '**' 0.01 '*' 0.05 '.' 0.1 ' ' 1
```

**SWEDEN**

```r
anova(time.dep.r[[18]],
      time.dep.r.rs[[18]])
```

```
## refitting model(s) with ML (instead of REML)

## Data: totdata
## Models:
## time.dep.r.rs[[18]]: z_tottime ~ logits * z_wler * z_joy * z_meta + logits * z_escs +
## time.dep.r.rs[[18]]:     logits * gender + (1 | user) + (1 | task)
## time.dep.r[[18]]: z_tottime ~ logits * z_wler * z_joy * z_meta + logits * z_escs +
## time.dep.r[[18]]:     logits * gender + (1 | user) + (1 | task) + (1 | schoolID)
##                     Df   AIC   BIC logLik deviance Chisq Chi Df Pr(>Chisq)
## time.dep.r.rs[[18]] 23 70601 70795 -35278    70555
## time.dep.r[[18]]    24 70521 70723 -35236    70473 82.53      1  < 2.2e-16
##
## time.dep.r.rs[[18]]
## time.dep.r[[18]]    ***
## ---
## Signif. codes:  0 '***' 0.001 '**' 0.01 '*' 0.05 '.' 0.1 ' ' 1
```

**POLAND**

```r
anova(time.dep.r[[19]],
      time.dep.r.rs[[19]])
```

```
## refitting model(s) with ML (instead of REML)
```

```
## Data: totdata
## Models:
## time.dep.r.rs[[19]]: z_tottime ~ logits * z_wler * z_joy * z_meta + logits * z_escs +
## time.dep.r.rs[[19]]:     logits * gender + (1 | user) + (1 | task)
## time.dep.r[[19]]: z_tottime ~ logits * z_wler * z_joy * z_meta + logits * z_escs +
## time.dep.r[[19]]:     logits * gender + (1 | user) + (1 | task) + (1 | schoolID)
##                     Df   AIC   BIC logLik deviance  Chisq Chi Df
## time.dep.r.rs[[19]] 23 76470 76665 -38212    76424
## time.dep.r[[19]]    24 76143 76346 -38048    76095 329.04      1
##                     Pr(>Chisq)
## time.dep.r.rs[[19]]
## time.dep.r[[19]]     < 2.2e-16 ***
## ---
## Signif. codes:  0 '***' 0.001 '**' 0.01 '*' 0.05 '.' 0.1 ' ' 1
```

# Dependent variable average time on relevant pages

## Persons

### AUSTRALIA

```r
anova(reltime.dep.r[[1]],
      reltime.dep.r.rp[[1]])
```

```
## refitting model(s) with ML (instead of REML)
```

```
## Data: totdata
## Models:
## reltime.dep.r.rp[[1]]: z_reltime ~ logits * z_wler * z_joy * z_meta + logits * z_escs +
## reltime.dep.r.rp[[1]]:     logits * gender + (1 | task) + (1 | schoolID)
## reltime.dep.r[[1]]: z_reltime ~ logits * z_wler * z_joy * z_meta + logits * z_escs +
## reltime.dep.r[[1]]:     logits * gender + (1 | user) + (1 | task) + (1 | schoolID)
##                       Df    AIC    BIC logLik deviance  Chisq Chi Df
## reltime.dep.r.rp[[1]] 23 102203 102408 -51079   102157
## reltime.dep.r[[1]]    24  94929  95142 -47440    94881 7276.4      1
##                       Pr(>Chisq)
## reltime.dep.r.rp[[1]]
## reltime.dep.r[[1]]     < 2.2e-16 ***
## ---
## Signif. codes:  0 '***' 0.001 '**' 0.01 '*' 0.05 '.' 0.1 ' ' 1
```

### AUSTRIA

```r
anova(reltime.dep.r[[2]],
      reltime.dep.r.rp[[2]])
```

```
## refitting model(s) with ML (instead of REML)
```

```
## Data: totdata
## Models:
## reltime.dep.r.rp[[2]]: z_reltime ~ logits * z_wler * z_joy * z_meta + logits * z_escs +
## reltime.dep.r.rp[[2]]:     logits * gender + (1 | task) + (1 | schoolID)
## reltime.dep.r[[2]]: z_reltime ~ logits * z_wler * z_joy * z_meta + logits * z_escs +
## reltime.dep.r[[2]]:     logits * gender + (1 | user) + (1 | task) + (1 | schoolID)
##                       Df   AIC   BIC logLik deviance  Chisq Chi Df
## reltime.dep.r.rp[[2]] 23 93411 93612 -46682    93365
## reltime.dep.r[[2]]    24 89708 89919 -44830    89660 3704.2      1
##                       Pr(>Chisq)
## reltime.dep.r.rp[[2]]
## reltime.dep.r[[2]]     < 2.2e-16 ***
## ---
## Signif. codes:  0 '***' 0.001 '**' 0.01 '*' 0.05 '.' 0.1 ' ' 1
```

**BELGUIM**

```r
anova(reltime.dep.r[[3]],
      reltime.dep.r.rp[[3]])
```

```
## refitting model(s) with ML (instead of REML)
```

```
## Data: totdata
## Models:
## reltime.dep.r.rp[[3]]: z_reltime ~ logits * z_wler * z_joy * z_meta + logits * z_escs +
## reltime.dep.r.rp[[3]]:     logits * gender + (1 | task) + (1 | schoolID)
## reltime.dep.r[[3]]: z_reltime ~ logits * z_wler * z_joy * z_meta + logits * z_escs +
## reltime.dep.r[[3]]:     logits * gender + (1 | user) + (1 | task) + (1 | schoolID)
##                       Df   AIC   BIC logLik deviance  Chisq Chi Df
## reltime.dep.r.rp[[3]] 23 92670 92873 -46312    92624
## reltime.dep.r[[3]]    24 88526 88738 -44239    88478 4146.2      1
##                       Pr(>Chisq)
## reltime.dep.r.rp[[3]]
## reltime.dep.r[[3]]     < 2.2e-16 ***
## ---
## Signif. codes:  0 '***' 0.001 '**' 0.01 '*' 0.05 '.' 0.1 ' ' 1
```

**CHILE**

```r
anova(reltime.dep.r[[4]],
      reltime.dep.r.rp[[4]])
```

```
## refitting model(s) with ML (instead of REML)
```

```
## Data: totdata
## Models:
## reltime.dep.r.rp[[4]]: z_reltime ~ logits * z_wler * z_joy * z_meta + logits * z_escs +
## reltime.dep.r.rp[[4]]:     logits * gender + (1 | task) + (1 | schoolID)
## reltime.dep.r[[4]]: z_reltime ~ logits * z_wler * z_joy * z_meta + logits * z_escs +
## reltime.dep.r[[4]]:     logits * gender + (1 | user) + (1 | task) + (1 | schoolID)
##                       Df   AIC   BIC logLik deviance  Chisq Chi Df
## reltime.dep.r.rp[[4]] 23 58543 58734 -29249    58497
## reltime.dep.r[[4]]    24 56022 56220 -27987    55974 2523.8      1
##                       Pr(>Chisq)
## reltime.dep.r.rp[[4]]
## reltime.dep.r[[4]]     < 2.2e-16 ***
## ---
## Signif. codes:  0 '***' 0.001 '**' 0.01 '*' 0.05 '.' 0.1 ' ' 1
```

**COLOMBIA**

```r
anova(reltime.dep.r[[5]],
      reltime.dep.r.rp[[5]])
```

```
## refitting model(s) with ML (instead of REML)

## Data: totdata
## Models:
## reltime.dep.r.rp[[5]]: z_reltime ~ logits * z_wler * z_joy * z_meta + logits * z_escs +
## reltime.dep.r.rp[[5]]:     logits * gender + (1 | task) + (1 | schoolID)
## reltime.dep.r[[5]]: z_reltime ~ logits * z_wler * z_joy * z_meta + logits * z_escs +
## reltime.dep.r[[5]]:     logits * gender + (1 | user) + (1 | task) + (1 | schoolID)
##                       Df   AIC   BIC logLik deviance  Chisq Chi Df
## reltime.dep.r.rp[[5]] 23 47505 47690 -23729    47459
## reltime.dep.r[[5]]    24 45777 45970 -22865    45729 1729.4      1
##                       Pr(>Chisq)
## reltime.dep.r.rp[[5]]
## reltime.dep.r[[5]]     < 2.2e-16 ***
## ---
## Signif. codes:  0 '***' 0.001 '**' 0.01 '*' 0.05 '.' 0.1 ' ' 1
```

**DENMARK**

```r
anova(reltime.dep.r[[6]],
      reltime.dep.r.rp[[6]])
```

```
## refitting model(s) with ML (instead of REML)

## Data: totdata
## Models:
## reltime.dep.r.rp[[6]]: z_reltime ~ logits * z_wler * z_joy * z_meta + logits * z_escs +
## reltime.dep.r.rp[[6]]:     logits * gender + (1 | task) + (1 | schoolID)
## reltime.dep.r[[6]]: z_reltime ~ logits * z_wler * z_joy * z_meta + logits * z_escs +
## reltime.dep.r[[6]]:     logits * gender + (1 | user) + (1 | task) + (1 | schoolID)
##                       Df   AIC   BIC logLik deviance  Chisq Chi Df
## reltime.dep.r.rp[[6]] 23 44970 45155 -22462    44924
## reltime.dep.r[[6]]    24 42748 42941 -21350    42700 2224.6      1
##                       Pr(>Chisq)
## reltime.dep.r.rp[[6]]
## reltime.dep.r[[6]]     < 2.2e-16 ***
## ---
## Signif. codes:  0 '***' 0.001 '**' 0.01 '*' 0.05 '.' 0.1 ' ' 1
```

**SPAIN**

```r
anova(reltime.dep.r[[7]],
      reltime.dep.r.rp[[7]])
```

```
## refitting model(s) with ML (instead of REML)

## Data: totdata
## Models:
## reltime.dep.r.rp[[7]]: z_reltime ~ logits * z_wler * z_joy * z_meta + logits * z_escs +
## reltime.dep.r.rp[[7]]:     logits * gender + (1 | task) + (1 | schoolID)
## reltime.dep.r[[7]]: z_reltime ~ logits * z_wler * z_joy * z_meta + logits * z_escs +
## reltime.dep.r[[7]]:     logits * gender + (1 | user) + (1 | task) + (1 | schoolID)
##                       Df   AIC   BIC logLik deviance  Chisq Chi Df
## reltime.dep.r.rp[[7]] 23 60351 60543 -30153    60305
## reltime.dep.r[[7]]    24 57678 57879 -28815    57630 2674.9      1
##                       Pr(>Chisq)
## reltime.dep.r.rp[[7]]
## reltime.dep.r[[7]]     < 2.2e-16 ***
## ---
## Signif. codes:  0 '***' 0.001 '**' 0.01 '*' 0.05 '.' 0.1 ' ' 1
```

**FRANCE**

```r
anova(reltime.dep.r[[8]],
      reltime.dep.r.rp[[8]])
```

```
## refitting model(s) with ML (instead of REML)

## Data: totdata
## Models:
## reltime.dep.r.rp[[8]]: z_reltime ~ logits * z_wler * z_joy * z_meta + logits * z_escs +
## reltime.dep.r.rp[[8]]:     logits * gender + (1 | task) + (1 | schoolID)
## reltime.dep.r[[8]]: z_reltime ~ logits * z_wler * z_joy * z_meta + logits * z_escs +
## reltime.dep.r[[8]]:     logits * gender + (1 | user) + (1 | task) + (1 | schoolID)
##                       Df   AIC   BIC logLik deviance  Chisq Chi Df
## reltime.dep.r.rp[[8]] 23 42230 42415 -21092    42184
## reltime.dep.r[[8]]    24 40888 41081 -20420    40840 1343.7      1
##                       Pr(>Chisq)
## reltime.dep.r.rp[[8]]
## reltime.dep.r[[8]]     < 2.2e-16 ***
## ---
## Signif. codes:  0 '***' 0.001 '**' 0.01 '*' 0.05 '.' 0.1 ' ' 1
```

### HONG KONG-CHINA

```
anova(reltime.dep.r[[9]],
      reltime.dep.r.rp[[9]])
```

```
## refitting model(s) with ML (instead of REML)

## Data: totdata
## Models:
## reltime.dep.r.rp[[9]]: z_reltime ~ logits * z_wler * z_joy * z_meta + logits * z_escs +
## reltime.dep.r.rp[[9]]:     logits * gender + (1 | task) + (1 | schoolID)
## reltime.dep.r[[9]]: z_reltime ~ logits * z_wler * z_joy * z_meta + logits * z_escs +
## reltime.dep.r[[9]]:     logits * gender + (1 | user) + (1 | task) + (1 | schoolID)
##                       Df   AIC   BIC logLik deviance Chisq Chi Df
## reltime.dep.r.rp[[9]] 23 50156 50344 -25055    50110
## reltime.dep.r[[9]]    24 47316 47512 -23634    47268  2842      1
##                       Pr(>Chisq)
## reltime.dep.r.rp[[9]]
## reltime.dep.r[[9]]     < 2.2e-16 ***
## ---
## Signif. codes:  0 '***' 0.001 '**' 0.01 '*' 0.05 '.' 0.1 ' ' 1
```

### HUNGARY

```
anova(reltime.dep.r[[10]],
      reltime.dep.r.rp[[10]])
```

```
## refitting model(s) with ML (instead of REML)

## Data: totdata
## Models:
## reltime.dep.r.rp[[10]]: z_reltime ~ logits * z_wler * z_joy * z_meta + logits * z_escs +
## reltime.dep.r.rp[[10]]:     logits * gender + (1 | task) + (1 | schoolID)
## reltime.dep.r[[10]]: z_reltime ~ logits * z_wler * z_joy * z_meta + logits * z_escs +
## reltime.dep.r[[10]]:     logits * gender + (1 | user) + (1 | task) + (1 | schoolID)
##                        Df   AIC   BIC logLik deviance  Chisq Chi Df
## reltime.dep.r.rp[[10]] 23 62087 62280 -31021    62041
## reltime.dep.r[[10]]    24 58871 59072 -29411    58823 3218.6      1
##                        Pr(>Chisq)
## reltime.dep.r.rp[[10]]
## reltime.dep.r[[10]]     < 2.2e-16 ***
## ---
## Signif. codes:  0 '***' 0.001 '**' 0.01 '*' 0.05 '.' 0.1 ' ' 1
```

**IRELAND**

```r
anova(reltime.dep.r[[11]],
      reltime.dep.r.rp[[11]])
```

```
## refitting model(s) with ML (instead of REML)

## Data: totdata
## Models:
## reltime.dep.r.rp[[11]]: z_reltime ~ logits * z_wler * z_joy * z_meta + logits * z_escs +
## reltime.dep.r.rp[[11]]:     logits * gender + (1 | task) + (1 | schoolID)
## reltime.dep.r[[11]]: z_reltime ~ logits * z_wler * z_joy * z_meta + logits * z_escs +
## reltime.dep.r[[11]]:     logits * gender + (1 | user) + (1 | task) + (1 | schoolID)
##                        Df   AIC   BIC logLik deviance  Chisq Chi Df
## reltime.dep.r.rp[[11]] 23 49221 49408 -24587    49175
## reltime.dep.r[[11]]    24 46996 47191 -23474    46948 2226.7      1
##                        Pr(>Chisq)
## reltime.dep.r.rp[[11]]
## reltime.dep.r[[11]]     < 2.2e-16 ***
## ---
## Signif. codes:  0 '***' 0.001 '**' 0.01 '*' 0.05 '.' 0.1 ' ' 1
```

**ICELAND**

```r
anova(reltime.dep.r[[12]],
      reltime.dep.r.rp[[12]])
```

```
## refitting model(s) with ML (instead of REML)

## Data: totdata
## Models:
## reltime.dep.r.rp[[12]]: z_reltime ~ logits * z_wler * z_joy * z_meta + logits * z_escs +
## reltime.dep.r.rp[[12]]:     logits * gender + (1 | task) + (1 | schoolID)
## reltime.dep.r[[12]]: z_reltime ~ logits * z_wler * z_joy * z_meta + logits * z_escs +
## reltime.dep.r[[12]]:     logits * gender + (1 | user) + (1 | task) + (1 | schoolID)
##                        Df   AIC   BIC logLik deviance  Chisq Chi Df
## reltime.dep.r.rp[[12]] 23 33472 33651 -16713    33426
## reltime.dep.r[[12]]    24 31294 31481 -15623    31246 2180.5      1
##                        Pr(>Chisq)
## reltime.dep.r.rp[[12]]
## reltime.dep.r[[12]]     < 2.2e-16 ***
## ---
## Signif. codes:  0 '***' 0.001 '**' 0.01 '*' 0.05 '.' 0.1 ' ' 1
```

**JAPAN**

```r
anova(reltime.dep.r[[13]],
      reltime.dep.r.rp[[13]])
```

```
## refitting model(s) with ML (instead of REML)
```

```
## Data: totdata
## Models:
## reltime.dep.r.rp[[13]]: z_reltime ~ logits * z_wler * z_joy * z_meta + logits * z_escs +
## reltime.dep.r.rp[[13]]:     logits * gender + (1 | task) + (1 | schoolID)
## reltime.dep.r[[13]]: z_reltime ~ logits * z_wler * z_joy * z_meta + logits * z_escs +
## reltime.dep.r[[13]]:     logits * gender + (1 | user) + (1 | task) + (1 | schoolID)
##                        Df   AIC   BIC logLik deviance  Chisq Chi Df
## reltime.dep.r.rp[[13]] 23 38323 38506 -19138    38277
## reltime.dep.r[[13]]    24 36698 36890 -18325    36650 1626.2      1
##                        Pr(>Chisq)
## reltime.dep.r.rp[[13]]
## reltime.dep.r[[13]]     < 2.2e-16 ***
## ---
## Signif. codes:  0 '***' 0.001 '**' 0.01 '*' 0.05 '.' 0.1 ' ' 1
```

**KOREA**

```r
anova(reltime.dep.r[[14]],
      reltime.dep.r.rp[[14]])
```

```
## refitting model(s) with ML (instead of REML)
```

```
## Data: totdata
## Models:
## reltime.dep.r.rp[[14]]: z_reltime ~ logits * z_wler * z_joy * z_meta + logits * z_escs +
## reltime.dep.r.rp[[14]]:     logits * gender + (1 | task) + (1 | schoolID)
## reltime.dep.r[[14]]: z_reltime ~ logits * z_wler * z_joy * z_meta + logits * z_escs +
## reltime.dep.r[[14]]:     logits * gender + (1 | user) + (1 | task) + (1 | schoolID)
##                        Df   AIC   BIC logLik deviance  Chisq Chi Df
## reltime.dep.r.rp[[14]] 23 46407 46597 -23181    46361
## reltime.dep.r[[14]]    24 44180 44377 -22066    44132 2229.6      1
##                        Pr(>Chisq)
## reltime.dep.r.rp[[14]]
## reltime.dep.r[[14]]     < 2.2e-16 ***
## ---
## Signif. codes:  0 '***' 0.001 '**' 0.01 '*' 0.05 '.' 0.1 ' ' 1
```

### MACAO-CHINA

```r
anova(reltime.dep.r[[15]],
      reltime.dep.r.rp[[15]])
```

```
## refitting model(s) with ML (instead of REML)
```

```
## Data: totdata
## Models:
## reltime.dep.r.rp[[15]]: z_reltime ~ logits * z_wler * z_joy * z_meta + logits * z_escs +
## reltime.dep.r.rp[[15]]:     logits * gender + (1 | task) + (1 | schoolID)
## reltime.dep.r[[15]]: z_reltime ~ logits * z_wler * z_joy * z_meta + logits * z_escs +
## reltime.dep.r[[15]]:     logits * gender + (1 | user) + (1 | task) + (1 | schoolID)
##                        Df   AIC   BIC logLik deviance  Chisq Chi Df
## reltime.dep.r.rp[[15]] 23 83610 83811 -41782    83564
## reltime.dep.r[[15]]    24 79717 79927 -39835    79669 3894.6      1
##                        Pr(>Chisq)
## reltime.dep.r.rp[[15]]
## reltime.dep.r[[15]]     < 2.2e-16 ***
## ---
## Signif. codes:  0 '***' 0.001 '**' 0.01 '*' 0.05 '.' 0.1 ' ' 1
```

### NORWAY

```r
anova(reltime.dep.r[[16]],
      reltime.dep.r.rp[[16]])
```

```
## refitting model(s) with ML (instead of REML)
```

```
## Data: totdata
## Models:
## reltime.dep.r.rp[[16]]: z_reltime ~ logits * z_wler * z_joy * z_meta + logits * z_escs +
## reltime.dep.r.rp[[16]]:     logits * gender + (1 | task) + (1 | schoolID)
## reltime.dep.r[[16]]: z_reltime ~ logits * z_wler * z_joy * z_meta + logits * z_escs +
## reltime.dep.r[[16]]:     logits * gender + (1 | user) + (1 | task) + (1 | schoolID)
##                        Df   AIC   BIC logLik deviance  Chisq Chi Df
## reltime.dep.r.rp[[16]] 23 70715 70911 -35335    70669
## reltime.dep.r[[16]]    24 65795 65999 -32874    65747 4922.3      1
##                        Pr(>Chisq)
## reltime.dep.r.rp[[16]]
## reltime.dep.r[[16]]     < 2.2e-16 ***
## ---
## Signif. codes:  0 '***' 0.001 '**' 0.01 '*' 0.05 '.' 0.1 ' ' 1
```

**NEW ZEALAND**

```
anova(reltime.dep.r[[17]],
      reltime.dep.r.rp[[17]])
```

```
## refitting model(s) with ML (instead of REML)
```

```
## Data: totdata
## Models:
## reltime.dep.r.rp[[17]]: z_reltime ~ logits * z_wler * z_joy * z_meta + logits * z_escs +
## reltime.dep.r.rp[[17]]:     logits * gender + (1 | task) + (1 | schoolID)
## reltime.dep.r[[17]]: z_reltime ~ logits * z_wler * z_joy * z_meta + logits * z_escs +
## reltime.dep.r[[17]]:     logits * gender + (1 | user) + (1 | task) + (1 | schoolID)
##                        Df   AIC   BIC logLik deviance  Chisq Chi Df
## reltime.dep.r.rp[[17]] 23 60838 61031 -30396    60792
## reltime.dep.r[[17]]    24 57518 57719 -28735    57470 3322.2      1
##                        Pr(>Chisq)
## reltime.dep.r.rp[[17]]
## reltime.dep.r[[17]]     < 2.2e-16 ***
## ---
## Signif. codes:  0 '***' 0.001 '**' 0.01 '*' 0.05 '.' 0.1 ' ' 1
```

**SWEDEN**

```
anova(reltime.dep.r[[18]],
      reltime.dep.r.rp[[18]])
```

```
## refitting model(s) with ML (instead of REML)
```

```
## Data: totdata
## Models:
## reltime.dep.r.rp[[18]]: z_reltime ~ logits * z_wler * z_joy * z_meta + logits * z_escs +
## reltime.dep.r.rp[[18]]:     logits * gender + (1 | task) + (1 | schoolID)
## reltime.dep.r[[18]]: z_reltime ~ logits * z_wler * z_joy * z_meta + logits * z_escs +
## reltime.dep.r[[18]]:     logits * gender + (1 | user) + (1 | task) + (1 | schoolID)
##                        Df   AIC   BIC logLik deviance  Chisq Chi Df
## reltime.dep.r.rp[[18]] 23 64448 64642 -32201    64402
## reltime.dep.r[[18]]    24 59904 60106 -29928    59856 4545.9      1
##                        Pr(>Chisq)
## reltime.dep.r.rp[[18]]
## reltime.dep.r[[18]]     < 2.2e-16 ***
## ---
## Signif. codes:  0 '***' 0.001 '**' 0.01 '*' 0.05 '.' 0.1 ' ' 1
```

**POLAND**

```
anova(reltime.dep.r[[19]],
      reltime.dep.r.rp[[19]])
```

```
## refitting model(s) with ML (instead of REML)
```

```
## Data: totdata
## Models:
## reltime.dep.r.rp[[19]]: z_reltime ~ logits * z_wler * z_joy * z_meta + logits * z_escs +
## reltime.dep.r.rp[[19]]:     logits * gender + (1 | task) + (1 | schoolID)
## reltime.dep.r[[19]]: z_reltime ~ logits * z_wler * z_joy * z_meta + logits * z_escs +
## reltime.dep.r[[19]]:     logits * gender + (1 | user) + (1 | task) + (1 | schoolID)
##                        Df   AIC   BIC logLik deviance  Chisq Chi Df
## reltime.dep.r.rp[[19]] 23 68163 68357 -34058    68117
## reltime.dep.r[[19]]    24 64457 64660 -32204    64409 3707.6      1
##                        Pr(>Chisq)
## reltime.dep.r.rp[[19]]
## reltime.dep.r[[19]]     < 2.2e-16 ***
## ---
## Signif. codes:  0 '***' 0.001 '**' 0.01 '*' 0.05 '.' 0.1 ' ' 1
```

## Tasks

### AUSTRALIA

```r
anova(reltime.dep.r[[1]],
      reltime.dep.r.rt[[1]])
```

```
## refitting model(s) with ML (instead of REML)
```

```
## Data: totdata
## Models:
## reltime.dep.r.rt[[1]]: z_reltime ~ logits * z_wler * z_joy * z_meta + logits * z_escs +
## reltime.dep.r.rt[[1]]:     logits * gender + (1 | user) + (1 | schoolID)
## reltime.dep.r[[1]]: z_reltime ~ logits * z_wler * z_joy * z_meta + logits * z_escs +
## reltime.dep.r[[1]]:     logits * gender + (1 | user) + (1 | task) + (1 | schoolID)
##                       Df    AIC    BIC logLik deviance Chisq Chi Df
## reltime.dep.r.rt[[1]] 23 143977 144182 -71966   143931
## reltime.dep.r[[1]]    24  94929  95142 -47440    94881 49051      1
##                       Pr(>Chisq)
## reltime.dep.r.rt[[1]]
## reltime.dep.r[[1]]     < 2.2e-16 ***
## ---
## Signif. codes:  0 '***' 0.001 '**' 0.01 '*' 0.05 '.' 0.1 ' ' 1
```

### AUSTRIA

```r
anova(reltime.dep.r[[2]],
      reltime.dep.r.rt[[2]])
```

```
## refitting model(s) with ML (instead of REML)
```

```
## Data: totdata
## Models:
## reltime.dep.r.rt[[2]]: z_reltime ~ logits * z_wler * z_joy * z_meta + logits * z_escs +
## reltime.dep.r.rt[[2]]:     logits * gender + (1 | user) + (1 | schoolID)
## reltime.dep.r[[2]]: z_reltime ~ logits * z_wler * z_joy * z_meta + logits * z_escs +
## reltime.dep.r[[2]]:     logits * gender + (1 | user) + (1 | task) + (1 | schoolID)
##                       Df    AIC    BIC logLik deviance Chisq Chi Df
## reltime.dep.r.rt[[2]] 23 125910 126112 -62932   125864
## reltime.dep.r[[2]]    24  89708  89919 -44830    89660 36204      1
##                       Pr(>Chisq)
## reltime.dep.r.rt[[2]]
## reltime.dep.r[[2]]     < 2.2e-16 ***
## ---
## Signif. codes:  0 '***' 0.001 '**' 0.01 '*' 0.05 '.' 0.1 ' ' 1
```

### BELGUIM

```r
anova(reltime.dep.r[[3]],
      reltime.dep.r.rt[[3]])
```

```
## refitting model(s) with ML (instead of REML)

## Data: totdata
## Models:
## reltime.dep.r.rt[[3]]: z_reltime ~ logits * z_wler * z_joy * z_meta + logits * z_escs +
## reltime.dep.r.rt[[3]]:     logits * gender + (1 | user) + (1 | schoolID)
## reltime.dep.r[[3]]: z_reltime ~ logits * z_wler * z_joy * z_meta + logits * z_escs +
## reltime.dep.r[[3]]:     logits * gender + (1 | user) + (1 | task) + (1 | schoolID)
##                       Df    AIC    BIC logLik deviance Chisq Chi Df
## reltime.dep.r.rt[[3]] 23 137909 138112 -68931   137863
## reltime.dep.r[[3]]    24  88526  88738 -44239    88478 49385      1
##                       Pr(>Chisq)
## reltime.dep.r.rt[[3]]
## reltime.dep.r[[3]]     < 2.2e-16 ***
## ---
## Signif. codes:  0 '***' 0.001 '**' 0.01 '*' 0.05 '.' 0.1 ' ' 1
```

### CHILE

```r
anova(reltime.dep.r[[4]],
      reltime.dep.r.rt[[4]])
```

```
## refitting model(s) with ML (instead of REML)

## Data: totdata
## Models:
## reltime.dep.r.rt[[4]]: z_reltime ~ logits * z_wler * z_joy * z_meta + logits * z_escs +
## reltime.dep.r.rt[[4]]:     logits * gender + (1 | user) + (1 | schoolID)
## reltime.dep.r[[4]]: z_reltime ~ logits * z_wler * z_joy * z_meta + logits * z_escs +
## reltime.dep.r[[4]]:     logits * gender + (1 | user) + (1 | task) + (1 | schoolID)
##                       Df   AIC   BIC logLik deviance Chisq Chi Df
## reltime.dep.r.rt[[4]] 23 79567 79757 -39761    79521
## reltime.dep.r[[4]]    24 56022 56220 -27987    55974 23547      1
##                       Pr(>Chisq)
## reltime.dep.r.rt[[4]]
## reltime.dep.r[[4]]     < 2.2e-16 ***
## ---
## Signif. codes:  0 '***' 0.001 '**' 0.01 '*' 0.05 '.' 0.1 ' ' 1
```

**COLOMBIA**

```
anova(reltime.dep.r[[5]],
      reltime.dep.r.rt[[5]])
```

```
## refitting model(s) with ML (instead of REML)
```

```
## Data: totdata
## Models:
## reltime.dep.r.rt[[5]]: z_reltime ~ logits * z_wler * z_joy * z_meta + logits * z_escs +
## reltime.dep.r.rt[[5]]:     logits * gender + (1 | user) + (1 | schoolID)
## reltime.dep.r[[5]]: z_reltime ~ logits * z_wler * z_joy * z_meta + logits * z_escs +
## reltime.dep.r[[5]]:     logits * gender + (1 | user) + (1 | task) + (1 | schoolID)
##                       Df   AIC   BIC logLik deviance Chisq Chi Df
## reltime.dep.r.rt[[5]] 23 63392 63577 -31673    63346
## reltime.dep.r[[5]]    24 45777 45970 -22865    45729 17617      1
##                       Pr(>Chisq)
## reltime.dep.r.rt[[5]]
## reltime.dep.r[[5]]     < 2.2e-16 ***
## ---
## Signif. codes:  0 '***' 0.001 '**' 0.01 '*' 0.05 '.' 0.1 ' ' 1
```

**DENMARK**

```
anova(reltime.dep.r[[6]],
      reltime.dep.r.rt[[6]])
```

```
## refitting model(s) with ML (instead of REML)
```

```
## Data: totdata
## Models:
## reltime.dep.r.rt[[6]]: z_reltime ~ logits * z_wler * z_joy * z_meta + logits * z_escs +
## reltime.dep.r.rt[[6]]:     logits * gender + (1 | user) + (1 | schoolID)
## reltime.dep.r[[6]]: z_reltime ~ logits * z_wler * z_joy * z_meta + logits * z_escs +
## reltime.dep.r[[6]]:     logits * gender + (1 | user) + (1 | task) + (1 | schoolID)
##                       Df   AIC   BIC logLik deviance Chisq Chi Df
## reltime.dep.r.rt[[6]] 23 62483 62668 -31219    62437
## reltime.dep.r[[6]]    24 42748 42941 -21350    42700 19737      1
##                       Pr(>Chisq)
## reltime.dep.r.rt[[6]]
## reltime.dep.r[[6]]     < 2.2e-16 ***
## ---
## Signif. codes:  0 '***' 0.001 '**' 0.01 '*' 0.05 '.' 0.1 ' ' 1
```

**SPAIN**

```
anova(reltime.dep.r[[7]],
      reltime.dep.r.rt[[7]])
```

```
## refitting model(s) with ML (instead of REML)
```

```
## Data: totdata
## Models:
## reltime.dep.r.rt[[7]]: z_reltime ~ logits * z_wler * z_joy * z_meta + logits * z_escs +
## reltime.dep.r.rt[[7]]:     logits * gender + (1 | user) + (1 | schoolID)
## reltime.dep.r[[7]]: z_reltime ~ logits * z_wler * z_joy * z_meta + logits * z_escs +
## reltime.dep.r[[7]]:     logits * gender + (1 | user) + (1 | task) + (1 | schoolID)
##                       Df   AIC   BIC logLik deviance Chisq Chi Df
## reltime.dep.r.rt[[7]] 23 85085 85277 -42520    85039
## reltime.dep.r[[7]]    24 57678 57879 -28815    57630 27409      1
##                       Pr(>Chisq)
## reltime.dep.r.rt[[7]]
## reltime.dep.r[[7]]     < 2.2e-16 ***
## ---
## Signif. codes:  0 '***' 0.001 '**' 0.01 '*' 0.05 '.' 0.1 ' ' 1
```

**FRANCE**

```
anova(reltime.dep.r[[8]],
      reltime.dep.r.rt[[8]])
```

```
## refitting model(s) with ML (instead of REML)
```

```
## Data: totdata
## Models:
## reltime.dep.r.rt[[8]]: z_reltime ~ logits * z_wler * z_joy * z_meta + logits * z_escs +
## reltime.dep.r.rt[[8]]:     logits * gender + (1 | user) + (1 | schoolID)
## reltime.dep.r[[8]]: z_reltime ~ logits * z_wler * z_joy * z_meta + logits * z_escs +
## reltime.dep.r[[8]]:     logits * gender + (1 | user) + (1 | task) + (1 | schoolID)
##                       Df   AIC   BIC logLik deviance Chisq Chi Df
## reltime.dep.r.rt[[8]] 23 62806 62991 -31380    62760
## reltime.dep.r[[8]]    24 40888 41081 -20420    40840 21920      1
##                       Pr(>Chisq)
## reltime.dep.r.rt[[8]]
## reltime.dep.r[[8]]     < 2.2e-16 ***
## ---
## Signif. codes:  0 '***' 0.001 '**' 0.01 '*' 0.05 '.' 0.1 ' ' 1
```

### HONG KONG-CHINA

```
anova(reltime.dep.r[[9]],
      reltime.dep.r.rt[[9]])
```

```
## refitting model(s) with ML (instead of REML)
```

```
## Data: totdata
## Models:
## reltime.dep.r.rt[[9]]: z_reltime ~ logits * z_wler * z_joy * z_meta + logits * z_escs +
## reltime.dep.r.rt[[9]]:     logits * gender + (1 | user) + (1 | schoolID)
## reltime.dep.r[[9]]: z_reltime ~ logits * z_wler * z_joy * z_meta + logits * z_escs +
## reltime.dep.r[[9]]:     logits * gender + (1 | user) + (1 | task) + (1 | schoolID)
##                       Df   AIC   BIC logLik deviance Chisq Chi Df
## reltime.dep.r.rt[[9]] 23 70187 70375 -35070    70141
## reltime.dep.r[[9]]    24 47316 47512 -23634    47268 22873      1
##                       Pr(>Chisq)
## reltime.dep.r.rt[[9]]
## reltime.dep.r[[9]]     < 2.2e-16 ***
## ---
## Signif. codes:  0 '***' 0.001 '**' 0.01 '*' 0.05 '.' 0.1 ' ' 1
```

### HUNGARY

```
anova(reltime.dep.r[[10]],
      reltime.dep.r.rt[[10]])
```

```
## refitting model(s) with ML (instead of REML)
```

```
## Data: totdata
## Models:
## reltime.dep.r.rt[[10]]: z_reltime ~ logits * z_wler * z_joy * z_meta + logits * z_escs +
## reltime.dep.r.rt[[10]]:     logits * gender + (1 | user) + (1 | schoolID)
## reltime.dep.r[[10]]: z_reltime ~ logits * z_wler * z_joy * z_meta + logits * z_escs +
## reltime.dep.r[[10]]:     logits * gender + (1 | user) + (1 | task) + (1 | schoolID)
##                        Df   AIC   BIC logLik deviance Chisq Chi Df
## reltime.dep.r.rt[[10]] 23 87516 87709 -43735    87470
## reltime.dep.r[[10]]    24 58871 59072 -29411    58823 28647      1
##                        Pr(>Chisq)
## reltime.dep.r.rt[[10]]
## reltime.dep.r[[10]]     < 2.2e-16 ***
## ---
## Signif. codes:  0 '***' 0.001 '**' 0.01 '*' 0.05 '.' 0.1 ' ' 1
```

**IRELAND**

```r
anova(reltime.dep.r[[11]],
      reltime.dep.r.rt[[11]])
```

```
## refitting model(s) with ML (instead of REML)

## Data: totdata
## Models:
## reltime.dep.r.rt[[11]]: z_reltime ~ logits * z_wler * z_joy * z_meta + logits * z_escs +
## reltime.dep.r.rt[[11]]:     logits * gender + (1 | user) + (1 | schoolID)
## reltime.dep.r[[11]]: z_reltime ~ logits * z_wler * z_joy * z_meta + logits * z_escs +
## reltime.dep.r[[11]]:     logits * gender + (1 | user) + (1 | task) + (1 | schoolID)
##                        Df   AIC   BIC logLik deviance Chisq Chi Df
## reltime.dep.r.rt[[11]] 23 69226 69413 -34590    69180
## reltime.dep.r[[11]]    24 46996 47191 -23474    46948 22232      1
##                        Pr(>Chisq)
## reltime.dep.r.rt[[11]]
## reltime.dep.r[[11]]     < 2.2e-16 ***
## ---
## Signif. codes:  0 '***' 0.001 '**' 0.01 '*' 0.05 '.' 0.1 ' ' 1
```

**ICELAND**

```r
anova(reltime.dep.r[[12]],
      reltime.dep.r.rt[[12]])
```

```
## refitting model(s) with ML (instead of REML)

## Data: totdata
## Models:
## reltime.dep.r.rt[[12]]: z_reltime ~ logits * z_wler * z_joy * z_meta + logits * z_escs +
## reltime.dep.r.rt[[12]]:     logits * gender + (1 | user) + (1 | schoolID)
## reltime.dep.r[[12]]: z_reltime ~ logits * z_wler * z_joy * z_meta + logits * z_escs +
## reltime.dep.r[[12]]:     logits * gender + (1 | user) + (1 | task) + (1 | schoolID)
##                        Df   AIC   BIC logLik deviance Chisq Chi Df
## reltime.dep.r.rt[[12]] 23 47438 47617 -23696    47392
## reltime.dep.r[[12]]    24 31294 31481 -15623    31246 16146      1
##                        Pr(>Chisq)
## reltime.dep.r.rt[[12]]
## reltime.dep.r[[12]]     < 2.2e-16 ***
## ---
## Signif. codes:  0 '***' 0.001 '**' 0.01 '*' 0.05 '.' 0.1 ' ' 1
```

**JAPAN**

```r
anova(reltime.dep.r[[13]],
      reltime.dep.r.rt[[13]])
```

```
## refitting model(s) with ML (instead of REML)

## Data: totdata
## Models:
## reltime.dep.r.rt[[13]]: z_reltime ~ logits * z_wler * z_joy * z_meta + logits * z_escs +
## reltime.dep.r.rt[[13]]:     logits * gender + (1 | user) + (1 | schoolID)
## reltime.dep.r[[13]]: z_reltime ~ logits * z_wler * z_joy * z_meta + logits * z_escs +
## reltime.dep.r[[13]]:     logits * gender + (1 | user) + (1 | task) + (1 | schoolID)
##                        Df   AIC   BIC logLik deviance Chisq Chi Df
## reltime.dep.r.rt[[13]] 23 57680 57863 -28817    57634
## reltime.dep.r[[13]]    24 36698 36890 -18325    36650 20983      1
##                        Pr(>Chisq)
## reltime.dep.r.rt[[13]]
## reltime.dep.r[[13]]     < 2.2e-16 ***
## ---
## Signif. codes:  0 '***' 0.001 '**' 0.01 '*' 0.05 '.' 0.1 ' ' 1
```

**KOREA**

```r
anova(reltime.dep.r[[14]],
      reltime.dep.r.rt[[14]])
```

```
## refitting model(s) with ML (instead of REML)

## Data: totdata
## Models:
## reltime.dep.r.rt[[14]]: z_reltime ~ logits * z_wler * z_joy * z_meta + logits * z_escs +
## reltime.dep.r.rt[[14]]:     logits * gender + (1 | user) + (1 | schoolID)
## reltime.dep.r[[14]]: z_reltime ~ logits * z_wler * z_joy * z_meta + logits * z_escs +
## reltime.dep.r[[14]]:     logits * gender + (1 | user) + (1 | task) + (1 | schoolID)
##                        Df   AIC   BIC logLik deviance Chisq Chi Df
## reltime.dep.r.rt[[14]] 23 75136 75325 -37545    75090
## reltime.dep.r[[14]]    24 44180 44377 -22066    44132 30958      1
##                        Pr(>Chisq)
## reltime.dep.r.rt[[14]]
## reltime.dep.r[[14]]     < 2.2e-16 ***
## ---
## Signif. codes:  0 '***' 0.001 '**' 0.01 '*' 0.05 '.' 0.1 ' ' 1
```

### MACAO-CHINA

```r
anova(reltime.dep.r[[15]],
      reltime.dep.r.rt[[15]])
```

```
## refitting model(s) with ML (instead of REML)

## Data: totdata
## Models:
## reltime.dep.r.rt[[15]]: z_reltime ~ logits * z_wler * z_joy * z_meta + logits * z_escs +
## reltime.dep.r.rt[[15]]:     logits * gender + (1 | user) + (1 | schoolID)
## reltime.dep.r[[15]]: z_reltime ~ logits * z_wler * z_joy * z_meta + logits * z_escs +
## reltime.dep.r[[15]]:     logits * gender + (1 | user) + (1 | task) + (1 | schoolID)
##                        Df    AIC    BIC logLik deviance Chisq Chi Df
## reltime.dep.r.rt[[15]] 23 122375 122576 -61165   122329
## reltime.dep.r[[15]]    24  79717  79927 -39835    79669 42660      1
##                        Pr(>Chisq)
## reltime.dep.r.rt[[15]]
## reltime.dep.r[[15]]     < 2.2e-16 ***
## ---
## Signif. codes:  0 '***' 0.001 '**' 0.01 '*' 0.05 '.' 0.1 ' ' 1
```

### NORWAY

```r
anova(reltime.dep.r[[16]],
      reltime.dep.r.rt[[16]])
```

```
## refitting model(s) with ML (instead of REML)

## Data: totdata
## Models:
## reltime.dep.r.rt[[16]]: z_reltime ~ logits * z_wler * z_joy * z_meta + logits * z_escs +
## reltime.dep.r.rt[[16]]:     logits * gender + (1 | user) + (1 | schoolID)
## reltime.dep.r[[16]]: z_reltime ~ logits * z_wler * z_joy * z_meta + logits * z_escs +
## reltime.dep.r[[16]]:     logits * gender + (1 | user) + (1 | task) + (1 | schoolID)
##                        Df   AIC   BIC logLik deviance Chisq Chi Df
## reltime.dep.r.rt[[16]] 23 97206 97402 -48580    97160
## reltime.dep.r[[16]]    24 65795 65999 -32874    65747 31413      1
##                        Pr(>Chisq)
## reltime.dep.r.rt[[16]]
## reltime.dep.r[[16]]     < 2.2e-16 ***
## ---
## Signif. codes:  0 '***' 0.001 '**' 0.01 '*' 0.05 '.' 0.1 ' ' 1
```

**NEW ZEALAND**

```r
anova(reltime.dep.r[[17]],
      reltime.dep.r.rt[[17]])
```

```
## refitting model(s) with ML (instead of REML)

## Data: totdata
## Models:
## reltime.dep.r.rt[[17]]: z_reltime ~ logits * z_wler * z_joy * z_meta + logits * z_escs +
## reltime.dep.r.rt[[17]]:     logits * gender + (1 | user) + (1 | schoolID)
## reltime.dep.r[[17]]: z_reltime ~ logits * z_wler * z_joy * z_meta + logits * z_escs +
## reltime.dep.r[[17]]:     logits * gender + (1 | user) + (1 | task) + (1 | schoolID)
##                        Df   AIC   BIC logLik deviance Chisq Chi Df
## reltime.dep.r.rt[[17]] 23 86180 86373 -43067    86134
## reltime.dep.r[[17]]    24 57518 57719 -28735    57470 28664      1
##                        Pr(>Chisq)
## reltime.dep.r.rt[[17]]
## reltime.dep.r[[17]]     < 2.2e-16 ***
## ---
## Signif. codes:  0 '***' 0.001 '**' 0.01 '*' 0.05 '.' 0.1 ' ' 1
```

**SWEDEN**

```r
anova(reltime.dep.r[[18]],
      reltime.dep.r.rt[[18]])
```

```
## refitting model(s) with ML (instead of REML)

## Data: totdata
## Models:
## reltime.dep.r.rt[[18]]: z_reltime ~ logits * z_wler * z_joy * z_meta + logits * z_escs +
## reltime.dep.r.rt[[18]]:     logits * gender + (1 | user) + (1 | schoolID)
## reltime.dep.r[[18]]: z_reltime ~ logits * z_wler * z_joy * z_meta + logits * z_escs +
## reltime.dep.r[[18]]:     logits * gender + (1 | user) + (1 | task) + (1 | schoolID)
##                        Df   AIC   BIC logLik deviance Chisq Chi Df
## reltime.dep.r.rt[[18]] 23 90082 90276 -45018    90036
## reltime.dep.r[[18]]    24 59904 60106 -29928    59856 30180      1
##                        Pr(>Chisq)
## reltime.dep.r.rt[[18]]
## reltime.dep.r[[18]]     < 2.2e-16 ***
## ---
## Signif. codes:  0 '***' 0.001 '**' 0.01 '*' 0.05 '.' 0.1 ' ' 1
```

**POLAND**

```
anova(reltime.dep.r[[19]],
      reltime.dep.r.rt[[19]])
```

```
## refitting model(s) with ML (instead of REML)
```

```
## Data: totdata
## Models:
## reltime.dep.r.rt[[19]]: z_reltime ~ logits * z_wler * z_joy * z_meta + logits * z_escs +
## reltime.dep.r.rt[[19]]:     logits * gender + (1 | user) + (1 | schoolID)
## reltime.dep.r[[19]]: z_reltime ~ logits * z_wler * z_joy * z_meta + logits * z_escs +
## reltime.dep.r[[19]]:     logits * gender + (1 | user) + (1 | task) + (1 | schoolID)
##                        Df   AIC   BIC logLik deviance Chisq Chi Df
## reltime.dep.r.rt[[19]] 23 94643 94837 -47298    94597
## reltime.dep.r[[19]]    24 64457 64660 -32204    64409 30188      1
##                        Pr(>Chisq)
## reltime.dep.r.rt[[19]]
## reltime.dep.r[[19]]     < 2.2e-16 ***
## ---
## Signif. codes:  0 '***' 0.001 '**' 0.01 '*' 0.05 '.' 0.1 ' ' 1
```

## Schools

### AUSTRALIA

```
anova(reltime.dep.r[[1]],
      reltime.dep.r.rs[[1]])
```

```
## refitting model(s) with ML (instead of REML)
```

```
## Data: totdata
## Models:
## reltime.dep.r.rs[[1]]: z_reltime ~ logits * z_wler * z_joy * z_meta + logits * z_escs +
## reltime.dep.r.rs[[1]]:     logits * gender + (1 | user) + (1 | task)
## reltime.dep.r[[1]]: z_reltime ~ logits * z_wler * z_joy * z_meta + logits * z_escs +
## reltime.dep.r[[1]]:     logits * gender + (1 | user) + (1 | task) + (1 | schoolID)
##                        Df   AIC   BIC logLik deviance  Chisq Chi Df
## reltime.dep.r.rs[[1]] 23 95146 95350 -47550    95100
## reltime.dep.r[[1]]    24 94929 95142 -47440    94881 218.85      1
##                        Pr(>Chisq)
## reltime.dep.r.rs[[1]]
## reltime.dep.r[[1]]     < 2.2e-16 ***
## ---
## Signif. codes:  0 '***' 0.001 '**' 0.01 '*' 0.05 '.' 0.1 ' ' 1
```

### AUSTRIA

```
anova(reltime.dep.r[[2]],
      reltime.dep.r.rs[[2]])
```

```
## refitting model(s) with ML (instead of REML)
```

```
## Data: totdata
## Models:
## reltime.dep.r.rs[[2]]: z_reltime ~ logits * z_wler * z_joy * z_meta + logits * z_escs +
## reltime.dep.r.rs[[2]]:     logits * gender + (1 | user) + (1 | task)
## reltime.dep.r[[2]]: z_reltime ~ logits * z_wler * z_joy * z_meta + logits * z_escs +
## reltime.dep.r[[2]]:     logits * gender + (1 | user) + (1 | task) + (1 | schoolID)
##                        Df   AIC   BIC logLik deviance  Chisq Chi Df
## reltime.dep.r.rs[[2]] 23 90725 90927 -45339    90679
## reltime.dep.r[[2]]    24 89708 89919 -44830    89660 1018.6      1
##                        Pr(>Chisq)
## reltime.dep.r.rs[[2]]
## reltime.dep.r[[2]]     < 2.2e-16 ***
## ---
## Signif. codes:  0 '***' 0.001 '**' 0.01 '*' 0.05 '.' 0.1 ' ' 1
```

**BELGUIM**

```r
anova(reltime.dep.r[[3]],
      reltime.dep.r.rs[[3]])
```

```
## refitting model(s) with ML (instead of REML)
```

```
## Data: totdata
## Models:
## reltime.dep.r.rs[[3]]: z_reltime ~ logits * z_wler * z_joy * z_meta + logits * z_escs +
## reltime.dep.r.rs[[3]]:     logits * gender + (1 | user) + (1 | task)
## reltime.dep.r[[3]]: z_reltime ~ logits * z_wler * z_joy * z_meta + logits * z_escs +
## reltime.dep.r[[3]]:     logits * gender + (1 | user) + (1 | task) + (1 | schoolID)
##                       Df   AIC   BIC logLik deviance  Chisq Chi Df
## reltime.dep.r.rs[[3]] 23 88807 89010 -44380    88761
## reltime.dep.r[[3]]    24 88526 88738 -44239    88478 283.23      1
##                       Pr(>Chisq)
## reltime.dep.r.rs[[3]]
## reltime.dep.r[[3]]     < 2.2e-16 ***
## ---
## Signif. codes:  0 '***' 0.001 '**' 0.01 '*' 0.05 '.' 0.1 ' ' 1
```

**CHILE**

```r
anova(reltime.dep.r[[4]],
      reltime.dep.r.rs[[4]])
```

```
## refitting model(s) with ML (instead of REML)
```

```
## Data: totdata
## Models:
## reltime.dep.r.rs[[4]]: z_reltime ~ logits * z_wler * z_joy * z_meta + logits * z_escs +
## reltime.dep.r.rs[[4]]:     logits * gender + (1 | user) + (1 | task)
## reltime.dep.r[[4]]: z_reltime ~ logits * z_wler * z_joy * z_meta + logits * z_escs +
## reltime.dep.r[[4]]:     logits * gender + (1 | user) + (1 | task) + (1 | schoolID)
##                       Df   AIC   BIC logLik deviance  Chisq Chi Df
## reltime.dep.r.rs[[4]] 23 56231 56421 -28092    56185
## reltime.dep.r[[4]]    24 56022 56220 -27987    55974 211.09      1
##                       Pr(>Chisq)
## reltime.dep.r.rs[[4]]
## reltime.dep.r[[4]]     < 2.2e-16 ***
## ---
## Signif. codes:  0 '***' 0.001 '**' 0.01 '*' 0.05 '.' 0.1 ' ' 1
```

**COLOMBIA**

```r
anova(reltime.dep.r[[5]],
      reltime.dep.r.rs[[5]])
```

```
## refitting model(s) with ML (instead of REML)

## Data: totdata
## Models:
## reltime.dep.r.rs[[5]]: z_reltime ~ logits * z_wler * z_joy * z_meta + logits * z_escs +
## reltime.dep.r.rs[[5]]:     logits * gender + (1 | user) + (1 | task)
## reltime.dep.r[[5]]: z_reltime ~ logits * z_wler * z_joy * z_meta + logits * z_escs +
## reltime.dep.r[[5]]:     logits * gender + (1 | user) + (1 | task) + (1 | schoolID)
##                       Df   AIC   BIC logLik deviance  Chisq Chi Df
## reltime.dep.r.rs[[5]] 23 45958 46143 -22956    45912
## reltime.dep.r[[5]]    24 45777 45970 -22865    45729 182.33      1
##                       Pr(>Chisq)
## reltime.dep.r.rs[[5]]
## reltime.dep.r[[5]]     < 2.2e-16 ***
## ---
## Signif. codes:  0 '***' 0.001 '**' 0.01 '*' 0.05 '.' 0.1 ' ' 1
```

**DENMARK**

```r
anova(reltime.dep.r[[6]],
      reltime.dep.r.rs[[6]])
```

```
## refitting model(s) with ML (instead of REML)

## Data: totdata
## Models:
## reltime.dep.r.rs[[6]]: z_reltime ~ logits * z_wler * z_joy * z_meta + logits * z_escs +
## reltime.dep.r.rs[[6]]:     logits * gender + (1 | user) + (1 | task)
## reltime.dep.r[[6]]: z_reltime ~ logits * z_wler * z_joy * z_meta + logits * z_escs +
## reltime.dep.r[[6]]:     logits * gender + (1 | user) + (1 | task) + (1 | schoolID)
##                       Df   AIC   BIC logLik deviance  Chisq Chi Df
## reltime.dep.r.rs[[6]] 23 42940 43125 -21447    42894
## reltime.dep.r[[6]]    24 42748 42941 -21350    42700 193.68      1
##                       Pr(>Chisq)
## reltime.dep.r.rs[[6]]
## reltime.dep.r[[6]]     < 2.2e-16 ***
## ---
## Signif. codes:  0 '***' 0.001 '**' 0.01 '*' 0.05 '.' 0.1 ' ' 1
```

**SPAIN**

```r
anova(reltime.dep.r[[7]],
      reltime.dep.r.rs[[7]])
```

```
## refitting model(s) with ML (instead of REML)
```

```
## Data: totdata
## Models:
## reltime.dep.r.rs[[7]]: z_reltime ~ logits * z_wler * z_joy * z_meta + logits * z_escs +
## reltime.dep.r.rs[[7]]:     logits * gender + (1 | user) + (1 | task)
## reltime.dep.r[[7]]: z_reltime ~ logits * z_wler * z_joy * z_meta + logits * z_escs +
## reltime.dep.r[[7]]:     logits * gender + (1 | user) + (1 | task) + (1 | schoolID)
##                       Df   AIC   BIC logLik deviance  Chisq Chi Df
## reltime.dep.r.rs[[7]] 23 58112 58304 -29033    58066
## reltime.dep.r[[7]]    24 57678 57879 -28815    57630 435.69      1
##                       Pr(>Chisq)
## reltime.dep.r.rs[[7]]
## reltime.dep.r[[7]]     < 2.2e-16 ***
## ---
## Signif. codes:  0 '***' 0.001 '**' 0.01 '*' 0.05 '.' 0.1 ' ' 1
```

**FRANCE**

```r
anova(reltime.dep.r[[8]],
      reltime.dep.r.rs[[8]])
```

```
## refitting model(s) with ML (instead of REML)
```

```
## Data: totdata
## Models:
## reltime.dep.r.rs[[8]]: z_reltime ~ logits * z_wler * z_joy * z_meta + logits * z_escs +
## reltime.dep.r.rs[[8]]:     logits * gender + (1 | user) + (1 | task)
## reltime.dep.r[[8]]: z_reltime ~ logits * z_wler * z_joy * z_meta + logits * z_escs +
## reltime.dep.r[[8]]:     logits * gender + (1 | user) + (1 | task) + (1 | schoolID)
##                       Df   AIC   BIC logLik deviance  Chisq Chi Df
## reltime.dep.r.rs[[8]] 23 41086 41271 -20520    41040
## reltime.dep.r[[8]]    24 40888 41081 -20420    40840 199.72      1
##                       Pr(>Chisq)
## reltime.dep.r.rs[[8]]
## reltime.dep.r[[8]]     < 2.2e-16 ***
## ---
## Signif. codes:  0 '***' 0.001 '**' 0.01 '*' 0.05 '.' 0.1 ' ' 1
```

**HONG KONG-CHINA**

```r
anova(reltime.dep.r[[9]],
      reltime.dep.r.rs[[9]])
```

```
## refitting model(s) with ML (instead of REML)

## Data: totdata
## Models:
## reltime.dep.r.rs[[9]]: z_reltime ~ logits * z_wler * z_joy * z_meta + logits * z_escs +
## reltime.dep.r.rs[[9]]:     logits * gender + (1 | user) + (1 | task)
## reltime.dep.r[[9]]: z_reltime ~ logits * z_wler * z_joy * z_meta + logits * z_escs +
## reltime.dep.r[[9]]:     logits * gender + (1 | user) + (1 | task) + (1 | schoolID)
##                       Df   AIC   BIC logLik deviance  Chisq Chi Df
## reltime.dep.r.rs[[9]] 23 47411 47599 -23682    47365
## reltime.dep.r[[9]]    24 47316 47512 -23634    47268 96.723      1
##                       Pr(>Chisq)
## reltime.dep.r.rs[[9]]
## reltime.dep.r[[9]]     < 2.2e-16 ***
## ---
## Signif. codes:  0 '***' 0.001 '**' 0.01 '*' 0.05 '.' 0.1 ' ' 1
```

**HUNGARY**

```r
anova(reltime.dep.r[[10]],
      reltime.dep.r.rs[[10]])
```

```
## refitting model(s) with ML (instead of REML)

## Data: totdata
## Models:
## reltime.dep.r.rs[[10]]: z_reltime ~ logits * z_wler * z_joy * z_meta + logits * z_escs +
## reltime.dep.r.rs[[10]]:     logits * gender + (1 | user) + (1 | task)
## reltime.dep.r[[10]]: z_reltime ~ logits * z_wler * z_joy * z_meta + logits * z_escs +
## reltime.dep.r[[10]]:     logits * gender + (1 | user) + (1 | task) + (1 | schoolID)
##                        Df   AIC   BIC logLik deviance  Chisq Chi Df
## reltime.dep.r.rs[[10]] 23 59206 59399 -29580    59160
## reltime.dep.r[[10]]    24 58871 59072 -29411    58823 337.22      1
##                        Pr(>Chisq)
## reltime.dep.r.rs[[10]]
## reltime.dep.r[[10]]     < 2.2e-16 ***
## ---
## Signif. codes:  0 '***' 0.001 '**' 0.01 '*' 0.05 '.' 0.1 ' ' 1
```

**IRELAND**

```r
anova(reltime.dep.r[[11]],
      reltime.dep.r.rs[[11]])
```

```
## refitting model(s) with ML (instead of REML)

## Data: totdata
## Models:
## reltime.dep.r.rs[[11]]: z_reltime ~ logits * z_wler * z_joy * z_meta + logits * z_escs +
## reltime.dep.r.rs[[11]]:     logits * gender + (1 | user) + (1 | task)
## reltime.dep.r[[11]]: z_reltime ~ logits * z_wler * z_joy * z_meta + logits * z_escs +
## reltime.dep.r[[11]]:     logits * gender + (1 | user) + (1 | task) + (1 | schoolID)
##                        Df   AIC   BIC logLik deviance Chisq Chi Df
## reltime.dep.r.rs[[11]] 23 47072 47259 -23513    47026
## reltime.dep.r[[11]]    24 46996 47191 -23474    46948 77.92      1
##                        Pr(>Chisq)
## reltime.dep.r.rs[[11]]
## reltime.dep.r[[11]]     < 2.2e-16 ***
## ---
## Signif. codes:  0 '***' 0.001 '**' 0.01 '*' 0.05 '.' 0.1 ' ' 1
```

**ICELAND**

```r
anova(reltime.dep.r[[12]],
      reltime.dep.r.rs[[12]])
```

```
## refitting model(s) with ML (instead of REML)

## Data: totdata
## Models:
## reltime.dep.r.rs[[12]]: z_reltime ~ logits * z_wler * z_joy * z_meta + logits * z_escs +
## reltime.dep.r.rs[[12]]:     logits * gender + (1 | user) + (1 | task)
## reltime.dep.r[[12]]: z_reltime ~ logits * z_wler * z_joy * z_meta + logits * z_escs +
## reltime.dep.r[[12]]:     logits * gender + (1 | user) + (1 | task) + (1 | schoolID)
##                        Df   AIC   BIC logLik deviance  Chisq Chi Df
## reltime.dep.r.rs[[12]] 23 31432 31611 -15693    31386
## reltime.dep.r[[12]]    24 31294 31481 -15623    31246 140.09      1
##                        Pr(>Chisq)
## reltime.dep.r.rs[[12]]
## reltime.dep.r[[12]]     < 2.2e-16 ***
## ---
## Signif. codes:  0 '***' 0.001 '**' 0.01 '*' 0.05 '.' 0.1 ' ' 1
```

### JAPAN

```r
anova(reltime.dep.r[[13]],
      reltime.dep.r.rs[[13]])
```

```
## refitting model(s) with ML (instead of REML)
```

```
## Data: totdata
## Models:
## reltime.dep.r.rs[[13]]: z_reltime ~ logits * z_wler * z_joy * z_meta + logits * z_escs +
## reltime.dep.r.rs[[13]]:     logits * gender + (1 | user) + (1 | task)
## reltime.dep.r[[13]]: z_reltime ~ logits * z_wler * z_joy * z_meta + logits * z_escs +
## reltime.dep.r[[13]]:     logits * gender + (1 | user) + (1 | task) + (1 | schoolID)
##                        Df   AIC   BIC logLik deviance  Chisq Chi Df
## reltime.dep.r.rs[[13]] 23 36723 36906 -18338    36677
## reltime.dep.r[[13]]    24 36698 36890 -18325    36650 26.563      1
##                        Pr(>Chisq)
## reltime.dep.r.rs[[13]]
## reltime.dep.r[[13]]     2.551e-07 ***
## ---
## Signif. codes:  0 '***' 0.001 '**' 0.01 '*' 0.05 '.' 0.1 ' ' 1
```

### KOREA

```r
anova(reltime.dep.r[[14]],
      reltime.dep.r.rs[[14]])
```

```
## refitting model(s) with ML (instead of REML)
```

```
## Data: totdata
## Models:
## reltime.dep.r.rs[[14]]: z_reltime ~ logits * z_wler * z_joy * z_meta + logits * z_escs +
## reltime.dep.r.rs[[14]]:     logits * gender + (1 | user) + (1 | task)
## reltime.dep.r[[14]]: z_reltime ~ logits * z_wler * z_joy * z_meta + logits * z_escs +
## reltime.dep.r[[14]]:     logits * gender + (1 | user) + (1 | task) + (1 | schoolID)
##                        Df   AIC   BIC logLik deviance  Chisq Chi Df
## reltime.dep.r.rs[[14]] 23 44246 44435 -22100    44200
## reltime.dep.r[[14]]    24 44180 44377 -22066    44132 68.027      1
##                        Pr(>Chisq)
## reltime.dep.r.rs[[14]]
## reltime.dep.r[[14]]     < 2.2e-16 ***
## ---
## Signif. codes:  0 '***' 0.001 '**' 0.01 '*' 0.05 '.' 0.1 ' ' 1
```

### MACAO-CHINA

```
anova(reltime.dep.r[[15]],
      reltime.dep.r.rs[[15]])
```

```
## refitting model(s) with ML (instead of REML)
```

```
## Data: totdata
## Models:
## reltime.dep.r.rs[[15]]: z_reltime ~ logits * z_wler * z_joy * z_meta + logits * z_escs +
## reltime.dep.r.rs[[15]]:     logits * gender + (1 | user) + (1 | task)
## reltime.dep.r[[15]]: z_reltime ~ logits * z_wler * z_joy * z_meta + logits * z_escs +
## reltime.dep.r[[15]]:     logits * gender + (1 | user) + (1 | task) + (1 | schoolID)
##                         Df   AIC   BIC logLik deviance  Chisq Chi Df
## reltime.dep.r.rs[[15]] 23 79964 80164 -39959    79918
## reltime.dep.r[[15]]    24 79717 79927 -39835    79669 248.17      1
##                         Pr(>Chisq)
## reltime.dep.r.rs[[15]]
## reltime.dep.r[[15]]     < 2.2e-16 ***
## ---
## Signif. codes:  0 '***' 0.001 '**' 0.01 '*' 0.05 '.' 0.1 ' ' 1
```

### NORWAY

```
anova(reltime.dep.r[[16]],
      reltime.dep.r.rs[[16]])
```

```
## refitting model(s) with ML (instead of REML)
```

```
## Data: totdata
## Models:
## reltime.dep.r.rs[[16]]: z_reltime ~ logits * z_wler * z_joy * z_meta + logits * z_escs +
## reltime.dep.r.rs[[16]]:     logits * gender + (1 | user) + (1 | task)
## reltime.dep.r[[16]]: z_reltime ~ logits * z_wler * z_joy * z_meta + logits * z_escs +
## reltime.dep.r[[16]]:     logits * gender + (1 | user) + (1 | task) + (1 | schoolID)
##                         Df   AIC   BIC logLik deviance  Chisq Chi Df
## reltime.dep.r.rs[[16]] 23 66125 66320 -33039    66079
## reltime.dep.r[[16]]    24 65795 65999 -32874    65747 331.37      1
##                         Pr(>Chisq)
## reltime.dep.r.rs[[16]]
## reltime.dep.r[[16]]     < 2.2e-16 ***
## ---
## Signif. codes:  0 '***' 0.001 '**' 0.01 '*' 0.05 '.' 0.1 ' ' 1
```

**NEW ZEALAND**

```r
anova(reltime.dep.r[[17]],
      reltime.dep.r.rs[[17]])
```

```
## refitting model(s) with ML (instead of REML)
```

```
## Data: totdata
## Models:
## reltime.dep.r.rs[[17]]: z_reltime ~ logits * z_wler * z_joy * z_meta + logits * z_escs +
## reltime.dep.r.rs[[17]]:     logits * gender + (1 | user) + (1 | task)
## reltime.dep.r[[17]]: z_reltime ~ logits * z_wler * z_joy * z_meta + logits * z_escs +
## reltime.dep.r[[17]]:     logits * gender + (1 | user) + (1 | task) + (1 | schoolID)
##                        Df   AIC   BIC logLik deviance  Chisq Chi Df
## reltime.dep.r.rs[[17]] 23 57628 57821 -28791    57582
## reltime.dep.r[[17]]    24 57518 57719 -28735    57470 112.21      1
##                        Pr(>Chisq)
## reltime.dep.r.rs[[17]]
## reltime.dep.r[[17]]     < 2.2e-16 ***
## ---
## Signif. codes:  0 '***' 0.001 '**' 0.01 '*' 0.05 '.' 0.1 ' ' 1
```

**SWEDEN**

```r
anova(reltime.dep.r[[18]],
      reltime.dep.r.rs[[18]])
```

```
## refitting model(s) with ML (instead of REML)
```

```
## Data: totdata
## Models:
## reltime.dep.r.rs[[18]]: z_reltime ~ logits * z_wler * z_joy * z_meta + logits * z_escs +
## reltime.dep.r.rs[[18]]:     logits * gender + (1 | user) + (1 | task)
## reltime.dep.r[[18]]: z_reltime ~ logits * z_wler * z_joy * z_meta + logits * z_escs +
## reltime.dep.r[[18]]:     logits * gender + (1 | user) + (1 | task) + (1 | schoolID)
##                        Df   AIC   BIC logLik deviance  Chisq Chi Df
## reltime.dep.r.rs[[18]] 23 59987 60181 -29971    59941
## reltime.dep.r[[18]]    24 59904 60106 -29928    59856 85.226      1
##                        Pr(>Chisq)
## reltime.dep.r.rs[[18]]
## reltime.dep.r[[18]]     < 2.2e-16 ***
## ---
## Signif. codes:  0 '***' 0.001 '**' 0.01 '*' 0.05 '.' 0.1 ' ' 1
```

**POLAND**

```r
anova(reltime.dep.r[[19]],
      reltime.dep.r.rs[[19]])
```

```
## refitting model(s) with ML (instead of REML)
```

```
## Data: totdata
## Models:
## reltime.dep.r.rs[[19]]: z_reltime ~ logits * z_wler * z_joy * z_meta + logits * z_escs +
## reltime.dep.r.rs[[19]]:     logits * gender + (1 | user) + (1 | task)
## reltime.dep.r[[19]]: z_reltime ~ logits * z_wler * z_joy * z_meta + logits * z_escs +
## reltime.dep.r[[19]]:     logits * gender + (1 | user) + (1 | task) + (1 | schoolID)
##                        Df   AIC   BIC logLik deviance  Chisq Chi Df
## reltime.dep.r.rs[[19]] 23 64775 64970 -32365    64729
## reltime.dep.r[[19]]    24 64457 64660 -32204    64409 320.14      1
##                        Pr(>Chisq)
## reltime.dep.r.rs[[19]]
## reltime.dep.r[[19]]     < 2.2e-16 ***
## ---
## Signif. codes:  0 '***' 0.001 '**' 0.01 '*' 0.05 '.' 0.1 ' ' 1
```
