# Supplementary material for: The Skilled, the Knowledgeable, and the Motivated: Investigating the Strategic Allocation of Time on Task in a Computer-Based Assessment
Source: Front Psychol. 2019 Jun 27;10:1429. doi: 10.3389/fpsyg.2019.01429 (PMC6660318; doi:10.3389/fpsyg.2019.01429)
Supplement: Supplementary file 4 [file Data_Sheet_4.pdf]

Supplement 4 to ‘The Skilled, the Knowledgeabeable, and the Motivated: Investigating the strategic allocation of time on task in a computer-based assessment’

Johannes Naumann

June 2019

Meta-analytic results for all theory-relevant effects

This supplement presents presents the full results of the meta-analyses for the theory-relevant effects (all main effects, interactions, and simple slopes for both total time on task and average time on relevant pages as dependent variables).

Main effects

Difficulty

Total time on task

```r
rma(main.dif.tottime[,1],main.dif.tottime[,2]^2)
```

```
##
## Random-Effects Model (k = 19; tau^2 estimator: REML)
##
## tau^2 (estimated amount of total heterogeneity): 0.0011 (SE = 0.0024)
## tau (square root of estimated tau^2 value):      0.0339
## I^2 (total heterogeneity / total variability):   15.63%
## H^2 (total variability / sampling variability):  1.19
##
## Test for Heterogeneity:
## Q(df = 18) = 20.8604, p-val = 0.2865
##
## Model Results:
##
## estimate      se     zval    pval   ci.lb   ci.ub
##   0.3931  0.0197  19.9479  <.0001  0.3545  0.4317  ***
##
## ---
## Signif. codes:  0 '***' 0.001 '**' 0.01 '*' 0.05 '.' 0.1 ' ' 1
```

Average time on relevant pages

```r
rma(main.dif.reltime[,1],main.dif.tottime[,2]^2)
```

```
##
## Random-Effects Model (k = 19; tau^2 estimator: REML)
##
## tau^2 (estimated amount of total heterogeneity): 0 (SE = 0.0020)
## tau (square root of estimated tau^2 value):      0
## I^2 (total heterogeneity / total variability):   0.00%
## H^2 (total variability / sampling variability):  1.00
##
## Test for Heterogeneity:
## Q(df = 18) = 16.3036, p-val = 0.5714
##
## Model Results:
##
## estimate      se    zval    pval   ci.lb   ci.ub
##   0.1712  0.0180  9.4941  <.0001  0.1359  0.2066  ***
##
## ---
## Signif. codes:  0 '***' 0.001 '**' 0.01 '*' 0.05 '.' 0.1 ' ' 1
```

Comprehension skill

Total time on task

```r
rma(main.comp.tottime[,1],main.comp.tottime[,2]^2)
```

```
##
## Random-Effects Model (k = 19; tau^2 estimator: REML)
##
## tau^2 (estimated amount of total heterogeneity): 0.0008 (SE = 0.0003)
## tau (square root of estimated tau^2 value):      0.0289
## I^2 (total heterogeneity / total variability):   86.30%
## H^2 (total variability / sampling variability):  7.30
##
## Test for Heterogeneity:
## Q(df = 18) = 133.3906, p-val < .0001
##
## Model Results:
##
## estimate      se     zval    pval   ci.lb   ci.ub
##   0.0868  0.0072  12.0581  <.0001  0.0726  0.1009  ***
##
## ---
## Signif. codes:  0 '***' 0.001 '**' 0.01 '*' 0.05 '.' 0.1 ' ' 1
```

Average time on relevant pages

```r
rma(main.comp.reltime[,1],main.comp.reltime[,2]^2)
```

```
##
## Random-Effects Model (k = 19; tau^2 estimator: REML)
##
## tau^2 (estimated amount of total heterogeneity): 0.0005 (SE = 0.0002)
## tau (square root of estimated tau^2 value):      0.0223
## I^2 (total heterogeneity / total variability):   83.69%
## H^2 (total variability / sampling variability):  6.13
##
## Test for Heterogeneity:
## Q(df = 18) = 106.2603, p-val < .0001
##
## Model Results:
##
## estimate      se     zval    pval   ci.lb   ci.ub
##   0.0863  0.0056  15.3214  <.0001  0.0753  0.0974  ***
##
## ---
## Signif. codes:  0 '***' 0.001 '**' 0.01 '*' 0.05 '.' 0.1 ' ' 1
```

Knowledge of reading strategies

Total time on task

```r
rma(main.meta.tottime[,1],main.meta.tottime[,2]^2)
```

```
##
## Random-Effects Model (k = 19; tau^2 estimator: REML)
##
## tau^2 (estimated amount of total heterogeneity): 0.0002 (SE = 0.0001)
## tau (square root of estimated tau^2 value):      0.0132
## I^2 (total heterogeneity / total variability):   61.78%
## H^2 (total variability / sampling variability):  2.62
##
## Test for Heterogeneity:
## Q(df = 18) = 46.3142, p-val = 0.0003
##
## Model Results:
##
## estimate      se    zval    pval   ci.lb   ci.ub
##   0.0258  0.0039  6.5904  <.0001  0.0181  0.0335  ***
##
## ---
## Signif. codes:  0 '***' 0.001 '**' 0.01 '*' 0.05 '.' 0.1 ' ' 1
```

Average time on relevant pages

```r
rma(main.meta.reltime[,1],main.meta.reltime[,2]^2)
```

```
##
## Random-Effects Model (k = 19; tau^2 estimator: REML)
##
## tau^2 (estimated amount of total heterogeneity): 0.0001 (SE = 0.0001)
## tau (square root of estimated tau^2 value):      0.0110
## I^2 (total heterogeneity / total variability):   60.50%
## H^2 (total variability / sampling variability):  2.53
##
## Test for Heterogeneity:
## Q(df = 18) = 44.9582, p-val = 0.0004
##
## Model Results:
##
## estimate      se    zval    pval   ci.lb   ci.ub
##   0.0257  0.0033  7.8235  <.0001  0.0193  0.0321  ***
##
## ---
## Signif. codes:  0 '***' 0.001 '**' 0.01 '*' 0.05 '.' 0.1 ' ' 1
```

Enjoyment of reading

Total time on task

```r
rma(main.joy.tottime[,1],main.joy.tottime[,2]^2)
```

```
##
## Random-Effects Model (k = 19; tau^2 estimator: REML)
##
## tau^2 (estimated amount of total heterogeneity): 0.0002 (SE = 0.0001)
## tau (square root of estimated tau^2 value):      0.0151
## I^2 (total heterogeneity / total variability):   67.07%
## H^2 (total variability / sampling variability):  3.04
##
## Test for Heterogeneity:
## Q(df = 18) = 55.0101, p-val < .0001
##
## Model Results:
##
## estimate      se    zval    pval   ci.lb   ci.ub
##   0.0184  0.0043  4.3055  <.0001  0.0100  0.0268  ***
##
## ---
## Signif. codes:  0 '***' 0.001 '**' 0.01 '*' 0.05 '.' 0.1 ' ' 1
```

Average time on relevant pages

```r
rma(main.joy.reltime[,1],main.joy.reltime[,2]^2)
```

```
##
## Random-Effects Model (k = 19; tau^2 estimator: REML)
##
## tau^2 (estimated amount of total heterogeneity): 0.0001 (SE = 0.0001)
## tau (square root of estimated tau^2 value):      0.0118
## I^2 (total heterogeneity / total variability):   63.09%
## H^2 (total variability / sampling variability):  2.71
##
## Test for Heterogeneity:
## Q(df = 18) = 49.1143, p-val = 0.0001
##
## Model Results:
##
## estimate      se    zval    pval   ci.lb   ci.ub
##   0.0165  0.0034  4.7919  <.0001  0.0098  0.0233  ***
##
## ---
## Signif. codes:  0 '***' 0.001 '**' 0.01 '*' 0.05 '.' 0.1 ' ' 1
```

Interactions

Difficulty x Comprehension skill

Total time on task

```r
rma(int.comp.tottime[,1],int.comp.tottime[,2]^2)
```

```
##
## Random-Effects Model (k = 19; tau^2 estimator: REML)
##
## tau^2 (estimated amount of total heterogeneity): 0.0002 (SE = 0.0001)
## tau (square root of estimated tau^2 value):      0.0150
## I^2 (total heterogeneity / total variability):   91.70%
## H^2 (total variability / sampling variability):  12.05
##
## Test for Heterogeneity:
## Q(df = 18) = 195.2334, p-val < .0001
##
## Model Results:
##
## estimate      se     zval    pval   ci.lb   ci.ub
##   0.0872  0.0036  24.2081  <.0001  0.0801  0.0942  ***
##
## ---
## Signif. codes:  0 '***' 0.001 '**' 0.01 '*' 0.05 '.' 0.1 ' ' 1
```

Average time on relevant pages

```r
rma(int.comp.reltime[,1],int.comp.reltime[,2]^2)
```

```
##
## Random-Effects Model (k = 19; tau^2 estimator: REML)
##
## tau^2 (estimated amount of total heterogeneity): 0.0002 (SE = 0.0001)
## tau (square root of estimated tau^2 value):      0.0130
## I^2 (total heterogeneity / total variability):   91.82%
## H^2 (total variability / sampling variability):  12.22
##
## Test for Heterogeneity:
## Q(df = 18) = 191.4135, p-val < .0001
##
## Model Results:
##
## estimate      se     zval    pval   ci.lb   ci.ub
##   0.0760  0.0031  24.2740  <.0001  0.0699  0.0822  ***
##
## ---
## Signif. codes:  0 '***' 0.001 '**' 0.01 '*' 0.05 '.' 0.1 ' ' 1
```

Difficulty x Knowledge of reading strategies

Total time on task

```r
rma(int.meta.tottime[,1],int.meta.tottime[,2]^2)
```

```
##
## Random-Effects Model (k = 19; tau^2 estimator: REML)
##
## tau^2 (estimated amount of total heterogeneity): 0.0000 (SE = 0.0000)
## tau (square root of estimated tau^2 value):      0.0060
## I^2 (total heterogeneity / total variability):   66.87%
## H^2 (total variability / sampling variability):  3.02
##
## Test for Heterogeneity:
## Q(df = 18) = 55.8440, p-val < .0001
##
## Model Results:
##
## estimate      se     zval    pval   ci.lb   ci.ub
##   0.0212  0.0017  12.4275  <.0001  0.0178  0.0245  ***
##
## ---
## Signif. codes:  0 '***' 0.001 '**' 0.01 '*' 0.05 '.' 0.1 ' ' 1
```

Average time on relevant pages

```r
rma(int.meta.reltime[,1],int.meta.reltime[,2]^2)
```

```
##
## Random-Effects Model (k = 19; tau^2 estimator: REML)
##
## tau^2 (estimated amount of total heterogeneity): 0.0000 (SE = 0.0000)
## tau (square root of estimated tau^2 value):      0.0049
## I^2 (total heterogeneity / total variability):   64.25%
## H^2 (total variability / sampling variability):  2.80
##
## Test for Heterogeneity:
## Q(df = 18) = 51.5574, p-val < .0001
##
## Model Results:
##
## estimate      se     zval    pval   ci.lb   ci.ub
##   0.0185  0.0014  13.1140  <.0001  0.0158  0.0213  ***
##
## ---
## Signif. codes:  0 '***' 0.001 '**' 0.01 '*' 0.05 '.' 0.1 ' ' 1
```

Difficulty x Reading enjoyment

Total time on task

```r
rma(int.joy.tottime[,1],int.joy.tottime[,2]^2)
```

```
##
## Random-Effects Model (k = 19; tau^2 estimator: REML)
##
## tau^2 (estimated amount of total heterogeneity): 0.0001 (SE = 0.0000)
## tau (square root of estimated tau^2 value):      0.0082
## I^2 (total heterogeneity / total variability):   78.22%
## H^2 (total variability / sampling variability):  4.59
##
## Test for Heterogeneity:
## Q(df = 18) = 78.1034, p-val < .0001
##
## Model Results:
##
## estimate      se    zval    pval   ci.lb   ci.ub
##   0.0185  0.0021  8.6572  <.0001  0.0143  0.0227  ***
##
## ---
## Signif. codes:  0 '***' 0.001 '**' 0.01 '*' 0.05 '.' 0.1 ' ' 1
```

Average time on relevant pages

```r
rma(int.joy.reltime[,1],int.joy.reltime[,2]^2)
```

```
##
## Random-Effects Model (k = 19; tau^2 estimator: REML)
##
## tau^2 (estimated amount of total heterogeneity): 0.0001 (SE = 0.0000)
## tau (square root of estimated tau^2 value):      0.0071
## I^2 (total heterogeneity / total variability):   78.42%
## H^2 (total variability / sampling variability):  4.63
##
## Test for Heterogeneity:
## Q(df = 18) = 81.2955, p-val < .0001
##
## Model Results:
##
## estimate      se    zval    pval   ci.lb   ci.ub
##   0.0160  0.0019  8.6173  <.0001  0.0123  0.0196  ***
##
## ---
## Signif. codes:  0 '***' 0.001 '**' 0.01 '*' 0.05 '.' 0.1 ' ' 1
```

Simple slopes

Difficulty in strong comprehenders

Total time on task

```r
rma(main.dif.tottime.neg[,1],main.dif.tottime.neg[,2]^2)
```

```
##
## Random-Effects Model (k = 19; tau^2 estimator: REML)
##
## tau^2 (estimated amount of total heterogeneity): 0.0026 (SE = 0.0030)
## tau (square root of estimated tau^2 value):      0.0509
## I^2 (total heterogeneity / total variability):   29.20%
## H^2 (total variability / sampling variability):  1.41
##
## Test for Heterogeneity:
## Q(df = 18) = 24.0661, p-val = 0.1529
##
## Model Results:
##
## estimate      se     zval    pval   ci.lb   ci.ub
##   0.5659  0.0217  26.0749  <.0001  0.5234  0.6085  ***
##
## ---
## Signif. codes:  0 '***' 0.001 '**' 0.01 '*' 0.05 '.' 0.1 ' ' 1
```

Average time on relevant pages

```r
rma(main.dif.reltime.neg[,1],main.dif.reltime.neg[,2]^2)
```

```
##
## Random-Effects Model (k = 19; tau^2 estimator: REML)
##
## tau^2 (estimated amount of total heterogeneity): 0 (SE = 0.0055)
## tau (square root of estimated tau^2 value):      0
## I^2 (total heterogeneity / total variability):   0.00%
## H^2 (total variability / sampling variability):  1.00
##
## Test for Heterogeneity:
## Q(df = 18) = 7.4143, p-val = 0.9861
##
## Model Results:
##
## estimate      se     zval    pval   ci.lb   ci.ub
##   0.3333  0.0295  11.3044  <.0001  0.2755  0.3911  ***
##
## ---
## Signif. codes:  0 '***' 0.001 '**' 0.01 '*' 0.05 '.' 0.1 ' ' 1
```

Difficulty in poor comprehenders

Total time on task

```r
rma(main.dif.tottime.pos[,1],main.dif.tottime.pos[,2]^2)
```

```
##
## Random-Effects Model (k = 19; tau^2 estimator: REML)
##
## tau^2 (estimated amount of total heterogeneity): 0.0012 (SE = 0.0025)
## tau (square root of estimated tau^2 value):      0.0353
## I^2 (total heterogeneity / total variability):   16.51%
## H^2 (total variability / sampling variability):  1.20
##
## Test for Heterogeneity:
## Q(df = 18) = 22.7490, p-val = 0.2004
##
## Model Results:
##
## estimate      se     zval    pval   ci.lb   ci.ub
##   0.2237  0.0200  11.1975  <.0001  0.1845  0.2628  ***
##
## ---
## Signif. codes:  0 '***' 0.001 '**' 0.01 '*' 0.05 '.' 0.1 ' ' 1
```

Average time on relevant pages

```r
rma(main.dif.reltime.pos[,1],main.dif.reltime.pos[,2]^2)
```

```
##
## Random-Effects Model (k = 19; tau^2 estimator: REML)
##
## tau^2 (estimated amount of total heterogeneity): 0 (SE = 0.0055)
## tau (square root of estimated tau^2 value):      0
## I^2 (total heterogeneity / total variability):   0.00%
## H^2 (total variability / sampling variability):  1.00
##
## Test for Heterogeneity:
## Q(df = 18) = 6.5999, p-val = 0.9931
##
## Model Results:
##
## estimate      se    zval    pval    ci.lb   ci.ub
##   0.0349  0.0295  1.1831  0.2368  -0.0229  0.0927
##
## ---
## Signif. codes:  0 '***' 0.001 '**' 0.01 '*' 0.05 '.' 0.1 ' ' 1
```

Comprehension skill in hard tasks

Total time on task

```r
rma(main.dif.tottime.neg.l[,1],main.dif.tottime.neg.l[,2]^2)
```

```
##
## Random-Effects Model (k = 19; tau^2 estimator: REML)
##
## tau^2 (estimated amount of total heterogeneity): 0.0025 (SE = 0.0009)
## tau (square root of estimated tau^2 value):      0.0501
## I^2 (total heterogeneity / total variability):   92.20%
## H^2 (total variability / sampling variability):  12.82
##
## Test for Heterogeneity:
## Q(df = 18) = 218.5156, p-val < .0001
##
## Model Results:
##
## estimate      se     zval    pval   ci.lb   ci.ub
##   0.2577  0.0120  21.4580  <.0001  0.2342  0.2813  ***
##
## ---
## Signif. codes:  0 '***' 0.001 '**' 0.01 '*' 0.05 '.' 0.1 ' ' 1
```

Average time on relevant pages

```r
rma(main.dif.reltime.neg.l[,1],main.dif.reltime.neg.l[,2]^2)
```

```
##
## Random-Effects Model (k = 19; tau^2 estimator: REML)
##
## tau^2 (estimated amount of total heterogeneity): 0.0018 (SE = 0.0007)
## tau (square root of estimated tau^2 value):      0.0426
## I^2 (total heterogeneity / total variability):   92.06%
## H^2 (total variability / sampling variability):  12.60
##
## Test for Heterogeneity:
## Q(df = 18) = 202.6282, p-val < .0001
##
## Model Results:
##
## estimate      se     zval    pval   ci.lb   ci.ub
##   0.2354  0.0102  23.0464  <.0001  0.2153  0.2554  ***
##
## ---
## Signif. codes:  0 '***' 0.001 '**' 0.01 '*' 0.05 '.' 0.1 ' ' 1
```

Comprehension skill in easy tasks

Total time on task

```
rma(main.dif.tottime.pos.l[,1],main.dif.tottime.pos.l[,2]^2)
```

```
##
## Random-Effects Model (k = 19; tau^2 estimator: REML)
##
## tau^2 (estimated amount of total heterogeneity): 0.0009 (SE = 0.0004)
## tau (square root of estimated tau^2 value):      0.0303
## I^2 (total heterogeneity / total variability):   81.30%
## H^2 (total variability / sampling variability):  5.35
##
## Test for Heterogeneity:
## Q(df = 18) = 102.6910, p-val < .0001
##
## Model Results:
##
## estimate      se      zval    pval    ci.lb    ci.ub
##  -0.0840  0.0078  -10.8012  <.0001  -0.0992  -0.0687  ***
##
## ---
## Signif. codes:  0 '***' 0.001 '**' 0.01 '*' 0.05 '.' 0.1 ' ' 1
```

Average time on relevant pages

```
rma(main.dif.reltime.pos.l[,1],main.dif.reltime.pos.l[,2]^2)
```

```
##
## Random-Effects Model (k = 19; tau^2 estimator: REML)
##
## tau^2 (estimated amount of total heterogeneity): 0.0005 (SE = 0.0002)
## tau (square root of estimated tau^2 value):      0.0227
## I^2 (total heterogeneity / total variability):   76.83%
## H^2 (total variability / sampling variability):  4.32
##
## Test for Heterogeneity:
## Q(df = 18) = 79.8716, p-val < .0001
##
## Model Results:
##
## estimate      se      zval    pval    ci.lb    ci.ub
##  -0.0625  0.0060  -10.4340  <.0001  -0.0743  -0.0508  ***
##
## ---
## Signif. codes:  0 '***' 0.001 '**' 0.01 '*' 0.05 '.' 0.1 ' ' 1
```

Difficulty in high knowledge of reading strategy students

Total time on task

```r
rma(main.dif.tottime.neg.meta[,1],main.dif.tottime.neg.meta[,2]^2)
```

```
##
## Random-Effects Model (k = 19; tau^2 estimator: REML)
##
## tau^2 (estimated amount of total heterogeneity): 0.0011 (SE = 0.0024)
## tau (square root of estimated tau^2 value):      0.0336
## I^2 (total heterogeneity / total variability):   15.28%
## H^2 (total variability / sampling variability):  1.18
##
## Test for Heterogeneity:
## Q(df = 18) = 20.5580, p-val = 0.3023
##
## Model Results:
##
## estimate      se     zval    pval   ci.lb   ci.ub
##   0.4352  0.0198  21.9986  <.0001  0.3965  0.4740  ***
##
## ---
## Signif. codes:  0 '***' 0.001 '**' 0.01 '*' 0.05 '.' 0.1 ' ' 1
```

Average time on relevant pages

```r
rma(main.dif.reltime.neg.meta[,1],main.dif.reltime.neg.meta[,2]^2)
```

```
##
## Random-Effects Model (k = 19; tau^2 estimator: REML)
##
## tau^2 (estimated amount of total heterogeneity): 0 (SE = 0.0055)
## tau (square root of estimated tau^2 value):      0
## I^2 (total heterogeneity / total variability):   0.00%
## H^2 (total variability / sampling variability):  1.00
##
## Test for Heterogeneity:
## Q(df = 18) = 6.3346, p-val = 0.9946
##
## Model Results:
##
## estimate      se    zval    pval   ci.lb   ci.ub
##   0.2207  0.0295  7.4884  <.0001  0.1630  0.2785  ***
##
## ---
## Signif. codes:  0 '***' 0.001 '**' 0.01 '*' 0.05 '.' 0.1 ' ' 1
```

Difficulty in low knowledge of reading strategy students

Total time on task

```
rma(main.dif.tottime.pos.meta[,1],main.dif.tottime.pos.meta[,2]^2)
```

```
##
## Random-Effects Model (k = 19; tau^2 estimator: REML)
##
## tau^2 (estimated amount of total heterogeneity): 0.0014 (SE = 0.0026)
## tau (square root of estimated tau^2 value):      0.0379
## I^2 (total heterogeneity / total variability):   18.56%
## H^2 (total variability / sampling variability):  1.23
##
## Test for Heterogeneity:
## Q(df = 18) = 21.8649, p-val = 0.2380
##
## Model Results:
##
## estimate      se     zval    pval   ci.lb   ci.ub
##   0.3521  0.0202  17.4184  <.0001  0.3124  0.3917  ***
##
## ---
## Signif. codes:  0 '***' 0.001 '**' 0.01 '*' 0.05 '.' 0.1 ' ' 1
```

Average time on relevant pages

```
rma(main.dif.reltime.pos.meta[,1],main.dif.reltime.pos.meta[,2]^2)
```

```
##
## Random-Effects Model (k = 19; tau^2 estimator: REML)
##
## tau^2 (estimated amount of total heterogeneity): 0 (SE = 0.0055)
## tau (square root of estimated tau^2 value):      0
## I^2 (total heterogeneity / total variability):   0.00%
## H^2 (total variability / sampling variability):  1.00
##
## Test for Heterogeneity:
## Q(df = 18) = 6.4037, p-val = 0.9943
##
## Model Results:
##
## estimate      se    zval    pval   ci.lb   ci.ub
##   0.1474  0.0295  5.0003  <.0001  0.0896  0.2052  ***
##
## ---
## Signif. codes:  0 '***' 0.001 '**' 0.01 '*' 0.05 '.' 0.1 ' ' 1
```

Knowledge of reading strategies in hard tasks

Total time on task

```r
rma(main.dif.tottime.neg.l[,1],main.dif.tottime.neg.l[,2]^2)
```

```
##
## Random-Effects Model (k = 19; tau^2 estimator: REML)
##
## tau^2 (estimated amount of total heterogeneity): 0.0005 (SE = 0.0002)
## tau (square root of estimated tau^2 value):      0.0214
## I^2 (total heterogeneity / total variability):   71.96%
## H^2 (total variability / sampling variability):  3.57
##
## Test for Heterogeneity:
## Q(df = 18) = 65.0788, p-val < .0001
##
## Model Results:
##
## estimate      se     zval    pval   ci.lb   ci.ub
##   0.0677  0.0058  11.5840  <.0001  0.0562  0.0792  ***
##
## ---
## Signif. codes:  0 '***' 0.001 '**' 0.01 '*' 0.05 '.' 0.1 ' ' 1
```

Average time on relevant pages

```r
rma(main.dif.reltime.neg.l[,1],main.dif.reltime.neg.l[,2]^2)
```

```
##
## Random-Effects Model (k = 19; tau^2 estimator: REML)
##
## tau^2 (estimated amount of total heterogeneity): 0.0003 (SE = 0.0002)
## tau (square root of estimated tau^2 value):      0.0178
## I^2 (total heterogeneity / total variability):   70.60%
## H^2 (total variability / sampling variability):  3.40
##
## Test for Heterogeneity:
## Q(df = 18) = 61.1346, p-val < .0001
##
## Model Results:
##
## estimate      se     zval    pval   ci.lb   ci.ub
##   0.0623  0.0049  12.7487  <.0001  0.0528  0.0719  ***
##
## ---
## Signif. codes:  0 '***' 0.001 '**' 0.01 '*' 0.05 '.' 0.1 ' ' 1
```

Knowledge of reading strategies in easy tasks

Total time on task

```r
rma(main.dif.tottime.pos.l[,1],main.dif.tottime.pos.l[,2]^2)
```

```
##
## Random-Effects Model (k = 19; tau^2 estimator: REML)
##
## tau^2 (estimated amount of total heterogeneity): 0.0002 (SE = 0.0001)
## tau (square root of estimated tau^2 value):      0.0135
## I^2 (total heterogeneity / total variability):   50.74%
## H^2 (total variability / sampling variability):  2.03
##
## Test for Heterogeneity:
## Q(df = 18) = 36.7344, p-val = 0.0057
##
## Model Results:
##
## estimate      se     zval    pval    ci.lb    ci.ub
##  -0.0156  0.0044  -3.5419  0.0004  -0.0243  -0.0070  ***
##
## ---
## Signif. codes:  0 '***' 0.001 '**' 0.01 '*' 0.05 '.' 0.1 ' ' 1
```

Average time on relevant pages

```r
rma(main.dif.reltime.pos.l[,1],main.dif.reltime.pos.l[,2]^2)
```

```
##
## Random-Effects Model (k = 19; tau^2 estimator: REML)
##
## tau^2 (estimated amount of total heterogeneity): 0.0001 (SE = 0.0001)
## tau (square root of estimated tau^2 value):      0.0112
## I^2 (total heterogeneity / total variability):   48.75%
## H^2 (total variability / sampling variability):  1.95
##
## Test for Heterogeneity:
## Q(df = 18) = 35.3998, p-val = 0.0084
##
## Model Results:
##
## estimate      se     zval    pval    ci.lb    ci.ub
##  -0.0106  0.0037  -2.8471  0.0044  -0.0178  -0.0033  **
##
## ---
## Signif. codes:  0 '***' 0.001 '**' 0.01 '*' 0.05 '.' 0.1 ' ' 1
```

Difficulty in high reading enjoyment students

Total time on task

```r
rma(main.dif.tottime.neg.joy[,1],main.dif.tottime.neg.joy[,2]^2)
```

```
##
## Random-Effects Model (k = 19; tau^2 estimator: REML)
##
## tau^2 (estimated amount of total heterogeneity): 0.0014 (SE = 0.0025)
## tau (square root of estimated tau^2 value):      0.0377
## I^2 (total heterogeneity / total variability):   18.50%
## H^2 (total variability / sampling variability):  1.23
##
## Test for Heterogeneity:
## Q(df = 18) = 20.8851, p-val = 0.2852
##
## Model Results:
##
## estimate      se     zval    pval   ci.lb   ci.ub
##   0.4299  0.0202  21.3061  <.0001  0.3904  0.4695  ***
##
## ---
## Signif. codes:  0 '***' 0.001 '**' 0.01 '*' 0.05 '.' 0.1 ' ' 1
```

Average time on relevant pages

```r
rma(main.dif.reltime.neg.joy[,1],main.dif.reltime.neg.joy[,2]^2)
```

```
##
## Random-Effects Model (k = 19; tau^2 estimator: REML)
##
## tau^2 (estimated amount of total heterogeneity): 0 (SE = 0.0055)
## tau (square root of estimated tau^2 value):      0
## I^2 (total heterogeneity / total variability):   0.00%
## H^2 (total variability / sampling variability):  1.00
##
## Test for Heterogeneity:
## Q(df = 18) = 5.8429, p-val = 0.9968
##
## Model Results:
##
## estimate      se    zval    pval   ci.lb   ci.ub
##   0.2155  0.0295  7.3099  <.0001  0.1577  0.2732  ***
##
## ---
## Signif. codes:  0 '***' 0.001 '**' 0.01 '*' 0.05 '.' 0.1 ' ' 1
```

Difficulty in low reading enjoyment students

Total time on task

```r
rma(main.dif.tottime.pos.joy[,1],main.dif.tottime.pos.joy[,2]^2)
```

```
##
## Random-Effects Model (k = 19; tau^2 estimator: REML)
##
## tau^2 (estimated amount of total heterogeneity): 0.0015 (SE = 0.0026)
## tau (square root of estimated tau^2 value):      0.0388
## I^2 (total heterogeneity / total variability):   19.30%
## H^2 (total variability / sampling variability):  1.24
##
## Test for Heterogeneity:
## Q(df = 18) = 22.5377, p-val = 0.2090
##
## Model Results:
##
## estimate      se     zval    pval   ci.lb   ci.ub
##   0.3580  0.0203  17.6186  <.0001  0.3182  0.3979  ***
##
## ---
## Signif. codes:  0 '***' 0.001 '**' 0.01 '*' 0.05 '.' 0.1 ' ' 1
```

Average time on relevant pages

```r
rma(main.dif.reltime.pos.joy[,1],main.dif.reltime.pos.joy[,2]^2)
```

```
##
## Random-Effects Model (k = 19; tau^2 estimator: REML)
##
## tau^2 (estimated amount of total heterogeneity): 0 (SE = 0.0055)
## tau (square root of estimated tau^2 value):      0
## I^2 (total heterogeneity / total variability):   0.00%
## H^2 (total variability / sampling variability):  1.00
##
## Test for Heterogeneity:
## Q(df = 18) = 7.1681, p-val = 0.9886
##
## Model Results:
##
## estimate      se    zval    pval   ci.lb   ci.ub
##   0.1527  0.0295  5.1781  <.0001  0.0949  0.2105  ***
##
## ---
## Signif. codes:  0 '***' 0.001 '**' 0.01 '*' 0.05 '.' 0.1 ' ' 1
```

Reading enjoyment in hard tasks

Total time on task

```r
rma(main.dif.tottime.neg.l[,1],main.dif.tottime.neg.l[,2]^2)
```

```
##
## Random-Effects Model (k = 19; tau^2 estimator: REML)
##
## tau^2 (estimated amount of total heterogeneity): 0.0007 (SE = 0.0003)
## tau (square root of estimated tau^2 value):      0.0261
## I^2 (total heterogeneity / total variability):   78.68%
## H^2 (total variability / sampling variability):  4.69
##
## Test for Heterogeneity:
## Q(df = 18) = 84.1148, p-val < .0001
##
## Model Results:
##
## estimate      se    zval    pval   ci.lb   ci.ub
##   0.0548  0.0068  8.0551  <.0001  0.0415  0.0681  ***
##
## ---
## Signif. codes:  0 '***' 0.001 '**' 0.01 '*' 0.05 '.' 0.1 ' ' 1
```

Average time on relevant pages

```r
rma(main.dif.reltime.neg.l[,1],main.dif.reltime.neg.l[,2]^2)
```

```
##
## Random-Effects Model (k = 19; tau^2 estimator: REML)
##
## tau^2 (estimated amount of total heterogeneity): 0.0005 (SE = 0.0002)
## tau (square root of estimated tau^2 value):      0.0223
## I^2 (total heterogeneity / total variability):   78.55%
## H^2 (total variability / sampling variability):  4.66
##
## Test for Heterogeneity:
## Q(df = 18) = 84.3241, p-val < .0001
##
## Model Results:
##
## estimate      se    zval    pval   ci.lb   ci.ub
##   0.0479  0.0058  8.2453  <.0001  0.0365  0.0593  ***
##
## ---
## Signif. codes:  0 '***' 0.001 '**' 0.01 '*' 0.05 '.' 0.1 ' ' 1
```

Reading enjoyment in easy tasks

Total time on task

```r
rma(main.dif.tottime.pos.l[,1],main.dif.tottime.pos.l[,2]^2)
```

```
##
## Random-Effects Model (k = 19; tau^2 estimator: REML)
##
## tau^2 (estimated amount of total heterogeneity): 0.0002 (SE = 0.0001)
## tau (square root of estimated tau^2 value):      0.0152
## I^2 (total heterogeneity / total variability):   55.53%
## H^2 (total variability / sampling variability):  2.25
##
## Test for Heterogeneity:
## Q(df = 18) = 40.0766, p-val = 0.0020
##
## Model Results:
##
## estimate      se     zval    pval    ci.lb    ci.ub
##  -0.0176  0.0047  -3.7289  0.0002  -0.0269  -0.0084  ***
##
## ---
## Signif. codes:  0 '***' 0.001 '**' 0.01 '*' 0.05 '.' 0.1 ' ' 1
```

Average time on relevant pages

```r
rma(main.dif.reltime.pos.l[,1],main.dif.reltime.pos.l[,2]^2)
```

```
##
## Random-Effects Model (k = 19; tau^2 estimator: REML)
##
## tau^2 (estimated amount of total heterogeneity): 0.0001 (SE = 0.0001)
## tau (square root of estimated tau^2 value):      0.0118
## I^2 (total heterogeneity / total variability):   50.58%
## H^2 (total variability / sampling variability):  2.02
##
## Test for Heterogeneity:
## Q(df = 18) = 36.3576, p-val = 0.0063
##
## Model Results:
##
## estimate      se     zval    pval    ci.lb    ci.ub
##  -0.0145  0.0038  -3.7763  0.0002  -0.0220  -0.0070  ***
##
## ---
## Signif. codes:  0 '***' 0.001 '**' 0.01 '*' 0.05 '.' 0.1 ' ' 1
```
